# Potential of Vitamin B6 Dioxime Analogues to Act as Cholinesterase Ligands

**DOI:** 10.3390/ijms232113388

**Published:** 2022-11-02

**Authors:** Dajana Gašo Sokač, Antonio Zandona, Sunčica Roca, Dražen Vikić-Topić, Gabriela Lihtar, Nikola Maraković, Valentina Bušić, Zrinka Kovarik, Maja Katalinić

**Affiliations:** 1 Faculty of Food Technology Osijek, Josip Juraj Strossmayer University of Osijek, F. Kuhača 18, HR-31000 Osijek, Croatia; 2 Institute for Medical Research and Occupational Health, Ksaverska c. 2, HR-10001 Zagreb, Croatia; 3 NMR Centre, Ruđer Bošković Institute, Bijenička 54, HR-10000 Zagreb, Croatia; 4 Department of Natural and Health Sciences, Juraj Dobrila University of Pula, Zagrebačka 30, HR-52100 Pula, Croatia

**Keywords:** pyridoxal oxime, dioxime, AChE, BChE, reversible inhibition, reactivation, molecular docking, cytotoxicity

## Abstract

Seven pyridoxal dioxime quaternary salts (**1**–**7**) were synthesized with the aim of studying their interactions with human acetylcholinesterase (AChE) and butyrylcholinesterase (BChE). The synthesis was achieved by the quaternization of pyridoxal monooxime with substituted 2-bromoacetophenone oximes (phenacyl bromide oximes). All compounds, prepared in good yields (43–76%) and characterized by 1D and 2D NMR spectroscopy, were evaluated as reversible inhibitors of cholinesterase and/or reactivators of enzymes inhibited by toxic organophosphorus compounds. Their potency was compared with that of their monooxime analogues and medically approved oxime HI-6. The obtained pyridoxal dioximes were relatively weak inhibitors for both enzymes (*K*_i_ = 100–400 µM). The second oxime group in the structure did not improve the binding compared to the monooxime analogues. The same was observed for reactivation of VX-, tabun-, and paraoxon-inhibited AChE and BChE, where no significant efficiency burst was noted. In silico analysis and molecular docking studies connected the kinetic data to the structural features of the tested compound, showing that the low binding affinity and reactivation efficacy may be a consequence of a bulk structure hindering important reactive groups. The tested dioximes were non-toxic to human neuroblastoma cells (SH-SY5Y) and human embryonal kidney cells (HEK293).

## 1. Introduction

**Scheme 1:**
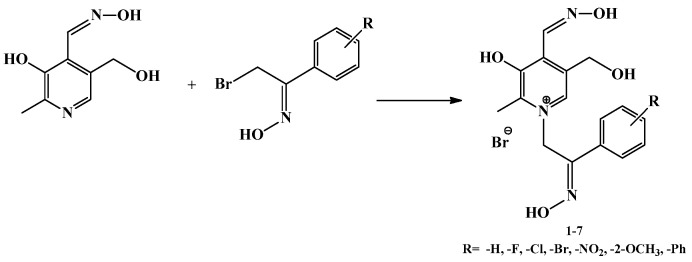
A general schematic representation of the preparation of pyridoxal dioximes.

**Figure 1:**
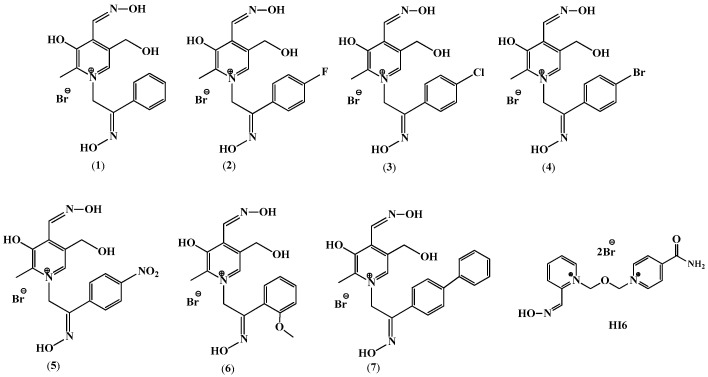
The chemical structures of the tested pyridoxal dioximes **1**–**7** and medically approved oxime HI-6.

Cholinesterases are ubiquitous enzymes involved in the regulation of neurotransmission and other physiological well-being processes. Acetylcholinesterase (AChE, EC 3.1.1.7) is an essential enzyme controlling neurotransmission in the cholinergic synapses of the central and peripheral nervous system by hydrolyzing the neurotransmitter acetylcholine (ACh) [1]. The exact role of butyrylcholinesterase (BChE, EC 3.1.1.8) has not yet been fully discovered. BChE is not as physiologically essential as AChE, but it is known that it participates in the detoxification of xenobiotics and bioactivation of several drugs [2]. In addition, BChE can hydrolyze the neurotransmitter ACh, and in the case of AChE inhibition or deficit, it can take over its role to an extent [3]. Accordingly, cholinesterases are the main targets in neurodegenerative disorders or diseases such as Alzheimer’s, Parkinson’s, myasthenia gravis, etc. Therefore, many different compounds are being investigated as cholinesterase inhibitors and potential neurotherapeutics, including oximes [4,5,6]. Oximes represent an important class in medicinal chemistry, well-known for their widespread applications as organophosphorus compound (OP) antidotes, drugs, and intermediates for the synthesis of several pharmacological derivatives [7]. In the past few decades, a whole series of aliphatic, aromatic, and heterocyclic oximes have been synthesized and characterized as cholinesterase ligands and antidotes in OP poisoning. The ability of oximes to bind to cholinesterases is linked to the quaternary nitrogen of the pyridine ring. On the other hand, the ability of oximes to reactivate OP-inhibited enzymes is a property of the oxime group(s) itself, which makes the nucleophilic displacement of the OP moiety from the enzyme’s active site possible [8]. Onwards, compounds based on vitamin B6 are described in the literature as being potentially biologically active against several targets such as cytochrome P450s, purinoreceptors, antibacterial proteins, and HIV [9,10]. Some vitamin B6 derivatives are even approved for use as pharmaceuticals such as pyritinol [11], pirisudanol [12], and cycletanin [13] or have reached the stage of clinical trials such as barucainide [14], EMD-2167 [15] and pyridoxal isonicotinoyl hydrazine [16,17]. They are used in the treatment of the nervous system (neuritis, radiculitis), cardiac and cerebrovascular insufficiency, depression, cerebral atherosclerosis, migraine, mild cognitive impairment and fatigue syndrome, diabetic nephropathy, diseases associated with an excessive presence of iron in organs and tissues, and hypertension [9]. Hydrazones and oximes possess intrinsic hydrolitiy stability resulting from the delocalization of electrons. Delocalization increases the negative charge on the carbon in the carbon–nitrogen bond, which reduces carbon electrophilicity and contributes to the increased hydrolytic stability [18]. In our previous study, we synthesized and characterized a series of vitamin B6 monooxime derivatives as inhibitors and reactivators of human AChE and BChE [19,20]. As the addition of a second oxime group in the structure may improve interactions with the enzyme [21,22,23], seven new pyridoxal-based dioximes were synthesized (Scheme 1, Figure 1) and their evaluation is described in the study presented here.

In addition, we performed an in silico analysis and molecular docking studies to connect the kinetic results to the structural features of the tested compounds. Additionally, we performed an in vitro cell viability screening on human neuroblastoma (SH-SY5Y) and human embryonal kidney cells (HEK293) to examine their possible cytotoxicity, which would prevent them from being candidates for further drug development.

## 2. Results

### 2.1. Pyridoxal Dioximes’ Synthesis

**Figure 2:**
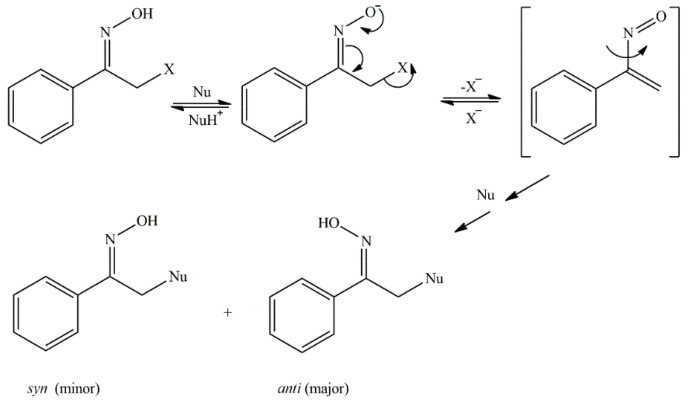
Schematic presentation of the S*_N_*1 mechanism occurring in pyridoxal dioxime synthesis.

New dioxime derivatives of vitamin B6, compounds **1**–**7**, were prepared in two steps, but the synthesis was attempted in two ways. First, a pyridoxal monooxime was quaternized with 2-bromoacetophenones, and then an attempt was made to convert the carbonyl group between the two aromatic rings into an oxime group. The reactions were not successful, and it was not possible to obtain a dioxime probably due to the steric hindrances of the two aromatic rings located near the carbonyl group. For this reason, instead of the sequence that includes the quaternization of pyridoxal monooxime and then the oximation of the resulting product, first, oximes of 2-bromoacetophenone were prepared, and then the quaternization reaction of pyridoxal oximes was carried out. The synthesis of oximes of 2-bromoacetophenone followed the method by Korten and Scholl, which is the reaction of the corresponding bromoacetophenones with the hydroxylamine hydrochloride in methanol [24], since predominantly *syn*-2-bromoacetophenone oximes are expected to be formed as a product. Oximes generally exist in the form of interconvertible *syn*- and *anti*-isomers depending on the orientation of groups around the C=N bond [25]. The explanation for the *anti*-configuration being more visible here can be found in research published earlier [26], which shows that the initial *syn-* configuration of the oxime (2-bromoacetophenone-oxime) in the reaction with a nucleophile changes dominantly to the thermodynamically less stable *anti-* configuration, regardless of the nature of the solvent or nucleophile. Furthermore, the reaction of 2-bromoacetophenone-oxime with pyridine in acetone gave a mixture of *syn-* and *anti-* products in a ratio of 10–30:90–70 [27]. The authors concluded that the reaction of nucleophilic substitution of acetophenone-oxime derivatives can take place by the S*_N_*1 and S*_N_*2 mechanism, depending on the nature of the nucleophile, the leaving group, the solvent, and the reaction conditions themselves. The S*_N_*1 mechanism is dominant with strongly basic nucleophiles and those containing nitrogen, and they are capable of removing a proton from the oxime group, and in this case, the *anti*-isomer is predominantly formed. The results confirmed that, regardless of the starting configuration of the oxime or the nature of the nucleophile, the S*_N_*1 mechanism always gives *anti*-oximes as the main product, and *α*-nitrosostyrene is an intermediate in S*_N_*1 reactions (Figure 2).

**Figure 3:**
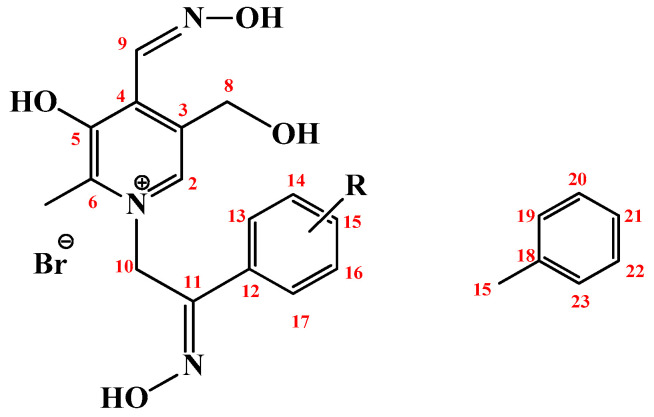
Enumeration scheme for the assignment of the NMR spectra when R=H, F, Cl, Br, NO_2_, OCH_3_, or phenyl.

Pyridoxal dioximes **1**–**7** were successfully prepared in the reaction of 2-bromoacetophenone oxime with pyridoxal monooximes while the reaction yields ranged between 43 (compound **2**) and 76% (compound **7**); Scheme 1, Table 1. The formation of the product was monitored by TLC chromatography and the structure of all the prepared dioximes was confirmed by 1D and 2D NMR spectroscopy. An enumeration scheme for the assignment of NMR spectra is given in Figure 3.

**Figure 4:**
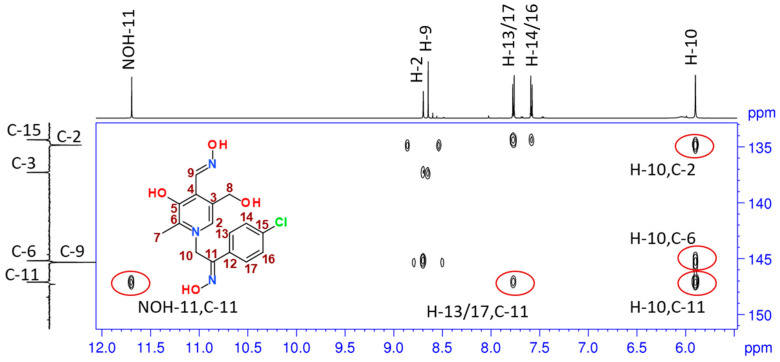
Part of the ^1^H-^13^C HMBC spectra (600 MHz, DMSO-d_6_) of **3**. The ovals indicate cross peaks, important for the assignment and confirmation of the synthesized compound. The part of the ^1^H NMR spectrum is shown at the top and part of the ^13^C NMR spectrum is shown at the left edge.

The NMR spectra obtained in DMSO-d_6_ confirmed the structure for all seven of the synthesized compounds (**1**–**7**). The most important information was found in the ^1^H-^13^C HMBC spectra (Figure 4). The cross peaks between the C-11 and NOH-11, H-13/17, and H-10 signals were very helpful in assigning the molecules. The cross peaks between the H-10 and C-2 and C-6 and C-11 atom signals confirmed a successful synthetic pathway.

The ^1^H and ^13^C spectra of compounds **3**, **4**, **6**, and **7** showed only one set of signals. Two isomeric forms, labeled (a) and (b) in Section 4, were found in the spectra of **1**, **2**, and **5**. The integral ratio between the isomers was 30:70 for **1** and 20:80 for **2** and **5** (Table 1). Significant differences in the chemical shifts between the observed isomer signals, corresponding to the equation: Δ*δ* = *δ*_isomer (**a**)_ − *δ*_isomer (**b**)_/ppm, were observed in the ^13^C spectra for the C-2 (Δ*δ* = 1.3 ppm), C-10 (Δ*δ* ≈ 6.8 ppm), C-11 (Δ*δ* ≈ −1.3 ppm), and C-12 (Δ*δ* ≈ −2.5 ppm) atom signals. Based on the position of the atoms in the molecule where changes were observed, the isomerism can be attributed to the *syn*/*anti* configurations of the NOH-11 group, which were also confirmed in their crystal structures [28]. The ratios of the isomers found in the crystal and in the solution were in agreement, and we assume that the *anti*-isomer is predominant. No differences in the cross peaks between the isomers were found in the recorded NOESY NMR spectra. The 1D and 2D NMR spectra are presented in the Supplementary Information (Figures S1–S36).

In addition to the successfully prepared dioximes **1**–**7**, an attempt was made to synthesize dioximes that have methyl and methoxy groups in position 4 on the benzene ring, but the products were formed in traces and could not be isolated from the reaction mixture.

### 2.2. Reversible Inhibition of AChE and BChE by Pyridoxal Dioximes

The results obtained for the reversible inhibition of cholinesterases by the tested compounds are summarized in Table 2. All of the tested pyridoxal dioximes **1**–**7** reversibly inhibited the activity of AChE and BChE. *K*_i_ was in the micromolar range, 90–400 µM. Not a single tested compound stood out as a strong inhibitor of AChE compared to the medically approved oxime HI-6 [8,29]. Interestingly, the addition of substituents on the phenyl ring decreased the AChE inhibition potency of pyridoxal dioximes. Although in the case of BChE oximes **1** and **7** were more potent inhibitors than HI-6, all of the compounds were rather poor inhibitors of BChE. Several compounds showed a modest selectivity in binding to AChE and BChE. The highest selectivity for binding to AChE was observed for chloro pyridoxal dioxime **3**, which preferred an up to 3-fold AChE over BChE (Table 2). The highest BChE preference, approximately 3-fold, was observed for the binding of phenyl pyridoxal dioxime **7**.

### 2.3. In Silico Analysis and Molecular Docking Studies

**Figure 5:**
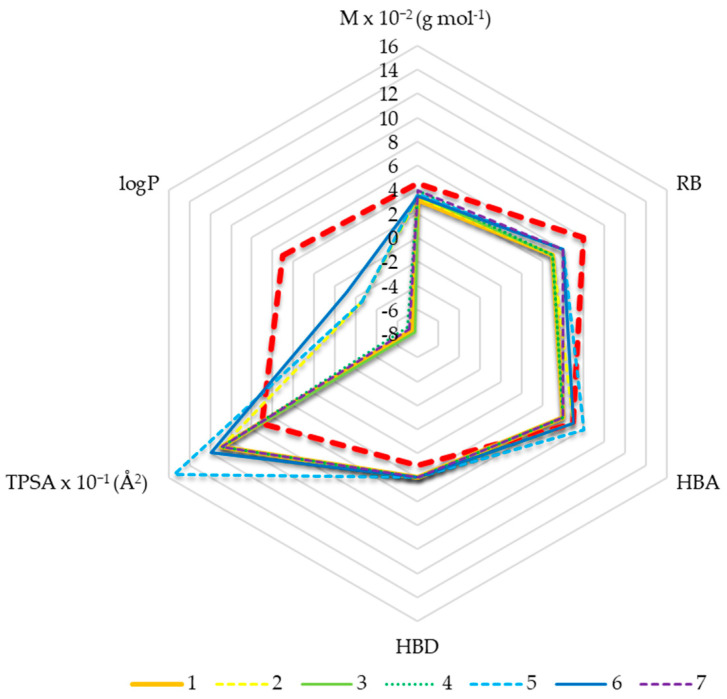
Physicochemical characteristics (molecular weight (*M*), number of hydrogen bond donors and acceptors (HBDs and HBAs), number of rotating bonds (RBs), lipophilicity (log*P*), and topological polar surface area (TPSA)) of pyridoxal dioximes in relation to the reference literature values given by the red dashed line [30].

Although it is known that charged compounds have a weak potential to passively cross the highly selective blood–brain barrier (BBB), it is evident that this also contributes to the poor biodistribution of these compounds. As pyridoxal dioximes were synthesized with the aim of studying their interactions with cholinesterases, especially AChE in the synapses of the central nervous system (CNS), we evaluated whether such compounds have the potential to pass BBB to act on the target of interest. The basic physicochemical properties were determined in silico and are presented in the form of the diagram in Figure 5. According to the results, dioximes possess characteristics such as the molecular weight (M), number of hydrogen bond acceptors (HBA), and number of rotating bonds (RB), which are within the recommended values for CNS active compounds [30]. Contrary, they exceed the limit of the number of hydrogen bond donors (HBD) and the topological polar surface area (TPSA). In addition, all compounds have a negative log*P* value, indicating low lipophilicity and therefore a low potential to be passively transported across BBB.

**Figure 6:**
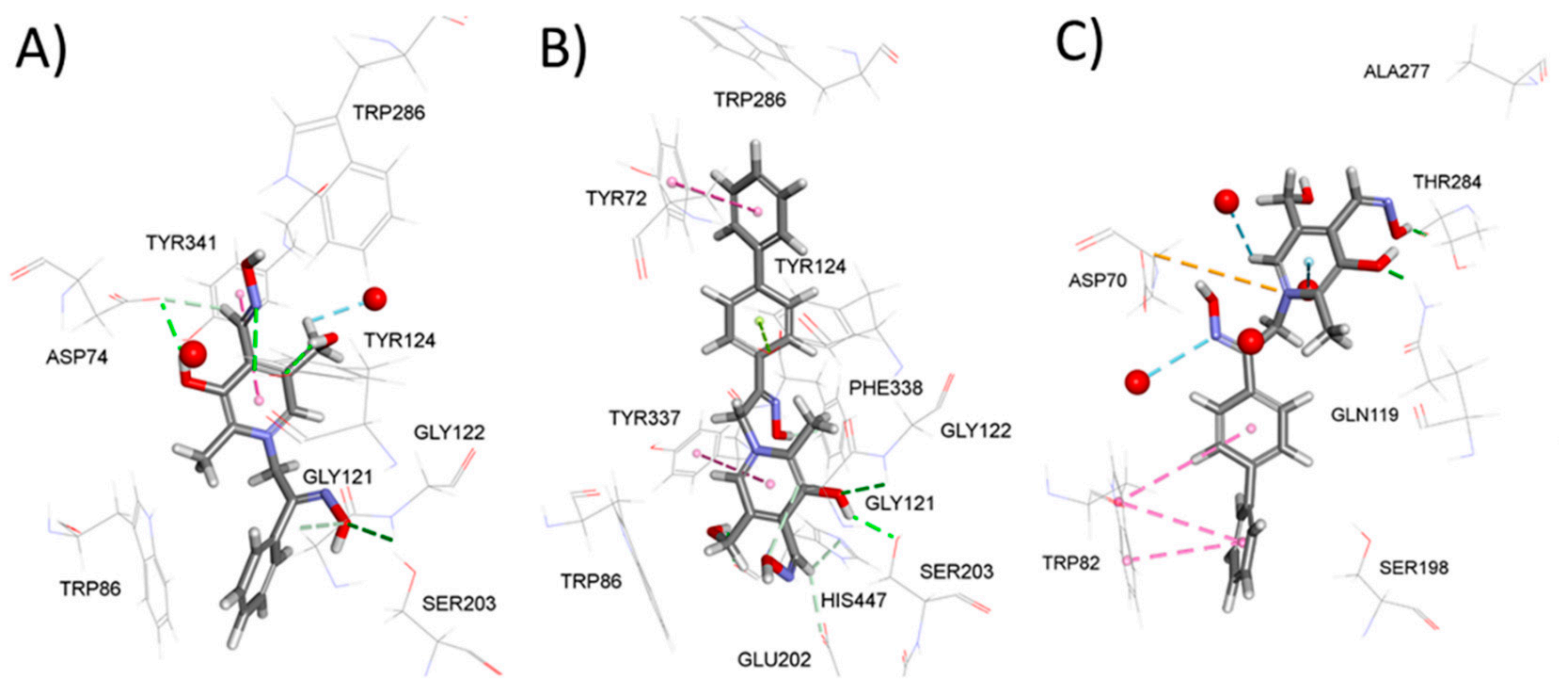
Close-up of the active site of the model complexes between AChE and compound **1** (**A**) or **7** (**B**) and BChE and compound **7** (**C**). Dashed lines represent different types of non-bonding interactions. Red spheres represent conserved water molecules. For clarity, only water molecules predicted to be engaged in non-bonding interactions are shown.

The molecular docking study was run to identify structural differences in the AChE/BChE-dioxime complexes governing the experimentally observed variation in the binding affinity among the selected dioximes. Figure 6A gives a close-up of the active site of the model complex between AChE and its most potent inhibitor from series **1**. Unsurprisingly, the pyridinium ring is placed in the vicinity of Trp86 from the choline binding site; however, aromatic interaction with its indole ring is not indicated, possibly because two aromatic rings were not perfectly parallel. The oxime group adjacent to the pyridinium ring forms two hydrogen bonds with Gly121 and Gly122 from the oxyanion hole. The pyridoxal ring is placed in the upper part of the active site gorge, with its oxime group and adjacent hydroxyl and hydroxymethyl group oriented towards the gorge opening. In doing so, it is engaged in conventional carbon hydrogen bonds with two neighboring water molecules and PAS residues Asp74 and Tyr124 together with *π–π* stacking interaction with PAS residue Tyr341. Taken together, compound **1** is predicted to establish 10 non-bonding interactions, 6 of which belong to strong conventional hydrogen bonds (the full list of non-bonding interactions for the docked compounds in this study is available in the Supplementary Material, Tables S1–S3). On the other hand, the least potent AChE inhibitor from the series, pyridoxal dioxime **7**, is predicted to bind in the opposite orientation with the diphenyl substituent in the upper part of the gorge and pyridoxal ring in the bottom of the gorge (Figure 6B), an orientation clearly driven by the voluminosity of the diphenyl group. In doing so, the diphenyl group is engaged in two weak non-bonding interactions with the peripheral anionic site (PAS) residues Tyr72 and Tyr124. The pyridoxal ring and the adjacent oxime group form multiple non-bonding interactions. However, only three of these interactions are conventional hydrogen bonds (with the residues Gly122, Ser203, and His447) while the rest are the weaker carbon and π-donor hydrogen bonds [19], explaining the 4-fold decrease in the inhibition potency compared with compound **1** despite the same number of non-bonding interactions (10). Interestingly, the same compound demonstrated the strongest inhibition potency towards BChE. The model complex between BChE and compound **7** predicts that **7** binds with the diphenyl substituent at the bottom of the gorge and the pyridoxal ring in the upper part of the gorge (Figure 6C), contrary to the orientation predicted in the case of BChE. This probably reflects a wider active site gorge in the case of BChE. Such an orientation of **7** allows multiple hydrophobic *π–π* interactions between the diphenyl moiety and Trp82 at the choline binding site, a well-known binding motif. On the other hand, the pyridoxal ring in the upper part of the gorge forms five hydrogen bonds with the neighboring residues and surrounding water molecules. However, the high inhibition potency of compound **7** is probably a reflection of the attractive electrostatic interaction between its *sp*^2^ nitrogen from the pyridoxal ring and the carboxylic group of Asp70, the strongest interaction predicted among all of the modeled complexes in this study.

### 2.4. Screening of Pyridoxal Dioximes as Reactivators of VX-, Tabun-, and Paraoxon-Inhibited Cholinesterases

We performed a basic screening of the reactivation potency of pyridoxal dioximes for AChE and BChE inhibited by VX, tabun, and paraoxon. The obtained results in terms of the observed first-order reactivation rate constant (*k*_obs_) achieved by 1 mM dioximes are presented in Table 3 for AChE and Table 4 for BChE. All seven of the tested pyridoxal dioximes were able to reactivate VX- and paraoxon-inhibited AChE and BChE in 24 h, reaching its maximal reactivation percentage between 25 and 95%; however, very low first-order reactivation rate constant *k*_obs_ was observed. For tabun, none of the pyridoxal dioximes showed a favorable result, reaching less than 20% of AChE and 10% of BChE reactivation within 24 h. The highest reactivation rates for AChE inhibited by paraoxon of 0.009 min^−1^ were observed in the case of compound **4**, with a reactivation maximum of 60% (Table 3). The highest reactivation rates for BChE inhibited by VX of 0.0025 min^−1^ were observed in the case of compound **7**, where the reactivation maximum of 90% was achieved within 24 h (Table 4). None of the tested pyridoxal dioximes were better reactivators of AChE or BChE than the medically approved oxime HI-6 [29].

### 2.5. Cytotoxicity

Since the tested compounds are analogues of vitamin B6, which has an important role in cells, they can cause disruption of the cell homeostasis. Therefore, we also checked whether these compounds induce cytotoxicity. The cytotoxic effect of pyridoxal dioximes was determined on the human neuronal SH-SY5Y and kidney HEK293 cells. The obtained results for 24 h cell exposure are summarized in Table 5. The tested compounds were not toxic in the tested concentration range. A modest cytotoxic effect, at the higher concentrations applied, was observed only for one of seven pyridoxal-derivatives, **3** (chloro pyridinium-dioxime), but only on HEK293 cells.

## 3. Discussion

The search for new, more effective inhibitors for cholinesterases is of great importance for the treatment of several neurodegenerative disorders and diseases [31], especially since over 55 million people suffer from such neurodegenerative disorders worldwide, and more than 10 million cases are registered every year [32]. With the aim of finding effective inhibitors for cholinesterases, involved in neurodegenerative pathologies, we synthesized a series of B6-based dioxime compounds. In our previous work, we showed that vitamin B6 (pyridoxal) monooximes act as cholinesterase inhibitors [20]. Additionally, based on previous results [21,22,33,34] showing that an additional oxime group can improve the binding properties up to 2-fold, we introduced the second oxime group into the structure instead of a carboxyl group in the linker. Moreover, compounds with the oxime group present an avenue for countermeasures in the case of organophosphate poisoning [32].

The target compounds **1**–**7** were prepared by the reaction of 2-bromoacetophenone oxime with a pyridoxal monooxime following the method by Korten and Scholl [24].

On the basis of earlier research, we concluded that the quaternization reaction in our case takes place by the S*_N_*1 mechanism and that two isomers can be formed that differ in the arrangement of the oxime group in the chain between the two aromatic rings. The formation of two isomers was confirmed by the NMR spectra in compounds **1**, **2**, and **5**. The NMR spectra of compounds **3**, **4**, **6**, and **7** show only one set of signals, which confirms the existence of only one form of the molecule.

To characterize the cholinesterase binding potential of compounds **1–7**, we first checked the affinity of free AChE and BChE for these compounds. As the results indicated, all tested analogues reversibly inhibited both AChE and BChE in the micromolar range. The highest inhibition potency was observed for compound **1** without additional motives such as halogens, nitro-, methoxy-, or phenyl-group. The obtained *K*_i_ constants for both enzymes place it in line with several currently investigated inhibitors [35,36,37,38,39,40]. Looking at each enzyme per se, pyridoxal dioximes were less effective inhibitors than their monooxime analogues [20], meaning that the addition of the second oxime group did not significantly improve the binding, as in our previous studies [21,22,34]. This result is probably due to the complexity of pyridoxal dioximes and the inability to position themselves equally well in the active site of the enzyme as pyridoxal monooximes analogues do [20]. The only opposite effect was observed for pyridoxal dioxime **1** and its equivalent monooxime. Furthermore, four of the pyridoxal dioximes showed a modest selectivity for binding to BChE, depending on their structure, while three preferred AChE. This is contrary to pyridoxal monooximes, which preferably bound to BChE [20]. Nevertheless, the additional oxime group reduced the selectivity of compounds **3**, **4**, and **5** towards BChE and increased it towards AChE while a reverse effect was seen for **6** and **7**.

As it is known that oxime compounds are used in medical practice as potential antidotes for OP poisoning, i.e., as reactivators of OP-inhibited cholinesterases [23,29,38,40,41], we evaluated their reactivation potency for AChE and BChE inhibited by VX, tabun, and paraoxon. The reactivation potential of the newly synthesized pyridoxal dioximes was low and they did not show any prominent reactivation compared with their monooxime analogues [20], other pyridinium-based oximes [21,23,39], or the medically approved oxime HI-6 currently used in the therapy of OP poisoning [8,25]. This means that the additional oxime group did not improve the reactivation potency and the pyridoxal dioxime scaffold is not recommended for further studies on OP antidote development. Furthermore, by in silico analysis, pyridioxal-dioximes are predicted not to have basic conditions for possible passive transport across the blood–brain barrier (BBB) [19]. It is assumed that this is certainly due to the positive charge in the structure and large TPSA. It must be emphasized that a permanent positive charge is an issue of pyridinium oximes since they do not cross the BBB at all or to a sufficient level [42]. To be more precise, removing the positive charge in some structure refinements of efficient oximes resulted in a better BBB passage [43,44,45]. However, having desirable properties for passage through BBB still do not necessarily mean that they will pass in a real in vivo system due to a complex brain protection molecular network [46,47].

Molecular docking studies identified the number and type of non-covalent interactions as the primary reason for the most pronounced differences observed in the reversible inhibition experiments, such as the more than 4-fold higher affinity of AChE towards compound **1** compared to **7** and the highest affinity of BChE toward the latter compared to the lowest affinity of AChE toward the same compound.

Since pyridoxal (vitamin B6) acts as a cofactor in different metabolic processes and therefore has important physiological roles [48], it can directly affect the dysregulation of cell processes and lead to cell decay. Thus, this additional essential information was assessed here by cytotoxicity screening studies, performed on two cell types, neuronal (as a main target site of oximes) and kidney (as a main organ that participates in the excretion of xenobiotics) [49,50]. As the results demonstrated, cytotoxicity was not observed for the tested compounds, indicating that they do not influence cell homeostasis or processes leading to cell death.

Based on the overall results, we can conclude that the additional oxime group for vitamin B6 dioximes did not increase the binding properties in terms of the observed inhibition and/or reactivation. Therefore, any further development of such compounds considered as cholinesterase ligands should undertake significant structure modifications of vitamin B6.

## 4. Materials and Methods

### 4.1. Synthesis and Structure Determination

2-Bromoacetophenone oximes were synthesized in the reaction of 2-bromoacetophenones with hydroxylamine hydrochloride in methanol [51]. To a hot solution of 2-bromoacetophenones (1 mmol) in MeOH, water was added dropwise until a slight turbidity was observed. Then, hydroxylamine hydrochloride (3 mmol) was added, and the resulting mixture was heated to boiling and allowed to stand for 24 h at room temperature. The precipitated product was filtered and washed with water and dried. Pyridoxal oxime (0.18 g; 1 mmol) was dissolved in acetone (50 mL) at 50 °C. The reaction mixture was cooled to room temperature and substituted 2-bromoacetophenone oxime was added (1 mmol) and allowed to stand at room temperature in the dark for 7 days (Scheme 1). The solution was then evaporated to a small volume and the crystalline crude product was collected by filtration under reduced pressure and recrystallized in acetone.

The NMR spectra were recorded using a Bruker AV600 spectrometer (Bruker BioSpin GmbH, Rheinstetten, Germany) at the Ruđer Bošković Institute and processed by the program TopSpin 4.1.2. A 5-mm broadband probe head with z-gradient coils operating at 600.130 MHz for ^1^H and 150.903 MHz for ^13^C was used. All spectra were measured from DMSO-d_6_ at 25 °C. The atom signal chemical shifts in the spectra were determined according to the solvent signal at 2.50 ppm in the ^1^H spectrum and at 39.51 ppm in the ^13^C spectrum, respectively. The individual resonances were assigned based on the recorded 2D NMR spectra: ^1^H-^1^H COSY (Correlation Spectroscopy), ^1^H-^13^C HMQC (Heteronuclear Multiple Quantum Coherence), and ^1^H-^13^C HMBC (Heteronuclear Multiple Bond Correlation).

The difference between the obtained isomers was assessed using the ^1^H-^1^H NOESY (Nuclear Overhauser Spectroscopy) spectrum.

The mass spectra were recorded on a low-resolution Agilent 6420 Triple Quadrupole mass spectrometer (Santa Clara, CA 95051, United States) with an electrospray ionization source in positive mode. The mass spectra are presented in the Supplementary Material (Figures S37–S43).

### 4.2. 3-Hydroxy-1-(2-hydroxyimino)-2-phenylethyl)-4-(hydroxyimino)methyl)-5-(hydroxymethyl)-2-methylpyridinium Bromide (***1***)

Yield 75%, mp 188–189.2 °C

Isomer **1a**. Presence in solution 70%.

^1^H (600 MHz, 25 °C, DMSO-*d*_6_): *δ* 13.09 (1H, br s, OH), 11.52 (1H, s, NOH-11), 8.68 (1H, s, H-2), 8.64 (1H, s, H-9), 7.70 (2H, dd, *J* = 8.44, 1.55 Hz, H-13 and H-17), 7.52–7.47 (3H, m, H-14, H-15 and H-16), 5.87 (2H, s, H-10), 4.79 (2H, s, H-8), 2.70 (3H, s, CH_3_-7) ppm. ^13^C (150 MHz, 25 °C, DMSO-*d*_6_): *δ* 152.5 (1C, C-5), 148.1 (1C, C-11), 145.4 (1C, C-9), 145.1 (1C, C-6), 137.2 (1C, C-3), 135.1 (1C, C-2), 130.5 (1C, C-12), 129.7 (1C, C-15), 128.4 (2C, C-14 and C-16), 128.2 (1C, C-4), 128.1 (2C, C-13 and C-17), 59.7 (1C, C-10), 58.5 (1C, C-8), 13.1 (1C, C-7) ppm. Molecular weight Calc. for [C_16_H_18_N_3_O_4_]^+^ = 316.1. Found ESI^+^-MS (MeOH) *m*/*z*: 316.2.

Isomer **1b**. Presence in solution 30%.

^1^H (600 MHz, 25 °C, DMSO-*d*_6_): *δ* 12.48 (1H, s, NOH-11), 8.54 (1H, s, H-9), 8.50 (1H, s, H-2), 7.64–7.61 (2H, m, H-13 and H-17), 7.41–7.38 (3H, m, H-14, H-15 and H-16), 5.98 (2H, s, H-10), 4.72 (2H, s, H-8), 2.63 (3H, s, H-7) ppm. ^13^C (150 MHz, 25 °C, DMSO-*d*_6_): *δ* 152.51 (1C, C-5), 149.3 (1C, C-11), 145.2 (1C, C-9), 144.6 (1C, C-6), 137.3 (1C, C-3), 133.7 (1C, C-2), 132.9 (1C, C-12), 129.1 (1C, C-15),128.7 (2C, C-14 and C-16) 127.8 (1C, C-4), 126.5 (2C, C-13 and C-17), 58.3 (1C, C-8), 53.0 (1C, C-10), 13.3 (1C, C-7) ppm. Molecular weight Calc. for [C_16_H_18_N_3_O_4_]^+^ = 316.1. Found ESI^+^-MS (MeOH) *m*/*z*: 316.2.

### 4.3. 1-(2-(4-Fluorophenyl)-2-(hydroxyimino)ethyl)-3hydroxy-4-((hydroxyimino)methyl)-2-methylpiridinium Bromide (***2***)

Yield 43%, mp 178–179.5 °C

Isomer **2a**. Presence in solution 80%.

^1^H (600 MHz, 25 °C, DMSO-*d*_6_): *δ* 13.2 (1H, br s, OH), 12.08 (1H, br s, OH), 11.64 (1H, s, N–OH-11), 8.73 (1H, s, H-2), 8.65 (1H, s, H-9), 7.82 (2H, dd, *J*_H,H_ = 9.04 Hz, *J*_H,F_ = 6.03 Hz, H-13 and H-17), 7.35 (2H, t, *J*_H,H_ = *J*_H,F_ = 8.61 Hz, H-14 and H-16), 5.92 (2H, s, H-10), 4.78 (2H, s, H-8), 2.71 (3H, s, CH_3_-7) ppm. ^13^C (150 MHz, 25 °C, DMSO-*d*_6_): δ 162.4 (1C, *J*_C,F_ = 248 Hz, C-15), 152.3 (1C, C-5), 147.1 (1C, C-11), 145.3 (1C, C-6), 145.2 (1C, C-9), 137.3 (1C, C-3), 135.2 (1C, C-2), 130.9 (2C, *J*_C,F_ = 8 Hz, C-13 and C-17), 128.5 (1C, C-4), 126.8 (1C, *J*_C,F_ = 3 Hz, C-12), 115.3 (2C, d, *J*_C,F_ = 22 Hz, C-14 and C-16), 59.6 (1C, C-10), 58.5 (1C, C-8), 13.3 (1C, C-7) ppm. Molecular weight *Calc.* for [C_16_H_17_FN_3_O_4_]^+^ = 334.1. Found ESI^+^-MS (MeOH) *m*/*z*: 334.1.

Isomer **2b**. Presence in solution 20%.

^1^H (600 MHz, 25 °C, DMSO-*d*_6_): *δ* 12.56 (1H, s, N–OH-11), 8.56 (1H, s, H-9), 8.55 (1H, s, H-2), 7.70 (2H, dd, *J*_H,H_ = 9.04 Hz, J_H,F_ = 5.60 Hz, H-13 and H-17), 7.23 (2H, t, *J*_H,H_ = *J*_H,F_ = 8.61 Hz, H-14 and H-16), 6.01 (2H, s, H-10), 4.71 (2H, s, H-8), 2.63 (3H, s, CH_3_-7) ppm. ^13^C (150 MHz, 25 °C, DMSO-*d*_6_): δ 162.8 (1C, *J*_C,F_ = 248 Hz, C-15), 152.5 (1C, C-5), 148.5 (1C, C-11), 145.1 (1C, C-9), 144.7 (1C, C-6), 137.3 (1C, C-3), 133.9 (1C, C-2), 130.9 (2C, *J*_C,F_ = 8 Hz, C-13 and C-17), 129.4 (1C, C-12), 128.0 (1C, C-4), 115.8 (2C, d, *J*_C,F_ = 22 Hz, C-14 and C-16), 58.3 (1C, C-8), 52.9 (1C, C-10), 13.3 (1C, C-7) ppm. Molecular weight Calc. for [C_16_H_17_FN_3_O_4_]^+^ = 334.1. Found ESI^+^-MS (MeOH) *m*/*z*: 334.1.

### 4.4. 1-(2-(4-Chlorophenyl)-2-(hydroxyimino)ethyl)-3-hydroxy-4-((hydroxyimino)methyl)-2-methylpiridinium Bromide (***3***)

Yield 68%, mp 198.2–199.0 °C. Presence in solution 100%.

^1^H (600 MHz, 25 °C, DMSO-*d*_6_): *δ* 11.69 (1H, s, NOH-11), 8.69 (1H, s, H-2), 8.65 (1H, s, H-9), 7.77 (2H, d, *J* = 8.67 Hz, H-13 and H-17), 7.58 (2H, d, *J* = 8.25 Hz, H-14 and H-16), 5.90 (2H, s, H-10), 4.78 (2H, s, H-8), 2.70 (3H, s, H-7) ppm. ^13^C (150 MHz, 25 °C, DMSO-*d*_6_): *δ* 152.8 (1C, C-5), 147.1 (1C, C-11), 145.3 (1C, C-6), 145.2 (1C, C-9), 137.3 (1C, C-3), 134.8 (1C, C-2), 134.4 (1C, C-15), 130.3 (2C, C-13 and C-17), 129.1 (1C, C-12), 128.44 (2C, C-14 and C-16), 128.38 (1C, C-4), 59.5 (1C, C-10), 58.5 (1C, C-8), 13.2 (1C, C-7) ppm. Molecular weight Calc. for [C_16_H_17_ClN_3_O_4_]^+^ = 350.1. Found ESI^+^-MS (MeOH) *m*/*z*: 350.1.

### 4.5. 1-(2-(4-Bromorophenyl)-2-(hydroxyimino)ethyl)-3-hydroxy-4-((hydroxyimino)methyl)-2-methylpiridinium Bromide (***4***)

Yield 71%, mp 197.5–198.0 °C. Presence in solution 100%.

^1^H (600 MHz, 25 °C, DMSO-*d*_6_): *δ* 11.63 (1H, s, NOH-11), 8.48 (1H, s, H-9), 7.66 (2H, d, *J* = 8.47 Hz, H-14 and H-16), 7.58 (2H, d, *J* = 8.47 Hz, H-13 and H-17), 7.47 (1H, s, H-2), 5.50 (2H, s, H-10), 4.57 (2H, s, H-8), 2.40 (3H, s, H-7) ppm. ^13^C (150 MHz, 25 °C, DMSO-*d*_6_): *δ* 165.6 (1C, C-5), 147.9 (1C, C-11), 146.9 (1C, C-9), 144.6 (1C, C-6), 136.5 (1C, C-3), 131.3 (2C, C-14 and C-16), 130.3 (2C, C-13 and C-17), 130.0 (1C, C-12), 125.6 (1C, C-4), 122.7 (1C, C-15), 119.4 (1C, C-2), 60.3 (1C, C-8), 59.4 (1C, C-10), 12.7 (1C, C-7) ppm Molecular weight Calc. for [C_16_H_17_BrN_3_O_4_]^+^ = 394.0. Found ESI^+^-MS (MeOH) *m*/*z*: 396.0.

### 4.6. 3-Hydroxy-1-(2-hydroxyimino)-2-(4-nitrophenyl)ethyl)-4-(hydroxyimino)methyl)-5-(hydroxymethyl)-2-methylpyridinium Bromide (***5***)

Yield 54%, mp 191.0–194.3 °C

Isomer **5a**. Presence in solution 80%.

^1^H (600 MHz, 25 °C, DMSO-*d*_6_): *δ* 13.15 (1H, br s, OH), 11.93 (1H, br s, NOH-11), 8.73 (1H, s, H-2), 8.65 (1H, s, H-9), 8.36 (2H, d, *J* = 8.65 Hz, H-14 and H-16), 8.00 (2H, d, *J* = 9.08 Hz, H-13 and H-17), 5.95 (2H, s, H-10), 4.79 (2H, s, H-8), 2.72 (3H, s, CH_3_-7) ppm. ^13^C (150 MHz, 25 °C, DMSO-*d*_6_): *δ* 152.56 (1C, C-5), 147.8 (1C, C-15), 147.0 (1C, C-11), 145.3 (2C, C-6 and C-9), 137.4 (1C, C-3), 136.7 (1C, C-12), 135.1 (1C, C-2), 129.9 (2C, C-13 and C-17), 128.5 (1C, C-4), 123.4 (2C, C-14 and C-16), 59.2 (1C, C-10), 58.5 (1C, C-8), 13.3 (1C, C-7) ppm. Molecular weight Calc. for [C_16_H_17_N_4_O_6_]^+^ = 361.1. Found ESI^+^-MS (MeOH) *m*/*z*: 361.2.

Isomer **5b**. Presence in solution 20%.

^1^H (600 MHz, 25 °C, DMSO-*d*_6_): *δ* 13.05 (1H, br s, OH), 8.57 (1H, s, H-9), 8.50 (1H, s, H-2), 8.25 (2H, d, *J* = 9.08 Hz, H-14 and H-16), 7.95 (2H, d, *J* = 9.08 Hz, H-13 and H-17), 6.05 (2H, s, H-10), 4.72 (2H, s, H-8), 2.65 (3H, s, CH_3_-7) ppm. ^13^C (150 MHz, 25 °C, DMSO-*d*_6_): *δ* 152.6 (1C, C-5), 148.0 (1C, C-11), 147.9 (1C, C-15), 145.2 (1C, C-9), 144.7 (1C, C-6), 139.2 (1C, C-12), 137.4 (1C, C-3), 133.5 (1C, C-2), 128.1 (1C, C-4), 127.8 (2C, C-13 and C-17), 123.9 (2C, C-14 and C-16), 58.3 (1C, C-8), 52.5 (1C, C-10), 13.4 (1C, C-7) ppm. Molecular weight Calc. for [C_16_H_17_N_4_O_6_]^+^ = 361.1. Found ESI^+^-MS (MeOH) *m*/*z*: 361.2.

### 4.7. 3-Hydroxy-1-(2-hydroxyimino)-2-(2-methoxyphenyl)ethyl)-4-(hydroxyimino)methyl)-5-(hydroxymethyl)-2-methylpyridinium Bromide (***6***)

Yield 53%, mp 156.0–157.5 °C. Presence in solution 100%.

^1^H (600 MHz, 25 °C, DMSO-*d*_6_): *δ* 13.02 (1H, br s, OH), 11.86 (1H, br s, OH), 11.30 (1H, s, NOH-11), 8.62 (1H, s, H-9), 8.58 (1H, s, H-2), 7.42 (1H, td, *J* = 7.71 Hz, 1.49 Hz, H-15), 7.29 (1H, dd, *J* = 7.71 Hz, 1.49 Hz, H-13), 7.11 (1H, d, *J* = 8.46 Hz, H-16), 7.03 (1H, t, *J* = 7.46 Hz, H-14), 5.94 (1H, br s, OH-8), 5.66 (2H, s, H-10), 4.78 (2H, s, H-8), 3.78 (3H, s, OCH_3_-17), 2.71 (3H, s, CH3-7) ppm. ^13^C (150 MHz, 25 °C, DMSO-*d*_6_): *δ* 155.8 (1C, C-17), 152.5 (1C, C-5), 148.2 (1C, C-11), 145.5 (1C, C-9), 145.1 (1C, C-6), 137.1 (1C, C-3), 135.1 (1C, C-2), 130.1 (1C, C-15), 129.5 (1C, C-13), 128.0 (1C, C-4), 120.4 (1C, C-14), 119.9 (1C, C-12), 111.5 (1C, C-16), 60.0 (1C, C-10), 58.4 (1C, C-8), 55.6 (1C, OCH_3_-17), 13.0 (1C, C-7) ppm. Molecular weight Calc. for [C_17_H_20_N_3_O_5_]^+^ = 346.1. Found ESI^+^-MS (MeOH) *m*/*z*: 346.2.

### 4.8. 3-Hydroxy-1-(2-hydroxyimino)-2-(4-phenylphenyl)ethyl)-4-(hydroxyimino)methyl)-5-(hydroxymethyl)-2-methylpyridinium Bromide (***7***)

Yield 76%, mp 220.2–220.5 °C. Presence in solution 100%.

^1^H (600 MHz, 25 °C, DMSO-*d*_6_): *δ* 12.85 (1H, br s, OH), 11.61 (1H, s, NOH-11), 8.74 (1H, s, H-2), 8.65 (1H, s, H-9), 7.86 (2H, d, *J* = 8.77 Hz, H-13 and H-17), 7.82 (2H, d, *J* = 8.77 Hz, H-14 and H-16), 7.73 (2H, d, *J* = 7.18 Hz, H-19 and H-23), 7.50 (2H, t, *J* = 7.58 Hz, H-20 and H-22), 7.41 (1H, t, *J* = 6.78 Hz, H-21), 5.96 (2H, s, H-10), 4.81 (2H, s, H-8), 2.72 (3H, s, H-7) ppm. ^13^C (150 MHz, 25 °C, DMSO-*d*_6_): *δ* 152.7 (1C, C-5), 147.6 (1C, C-11), 145.5 (1C, C-9), 145.1 (1C, C-6), 141.3 (1C, C-15), 139.2 (1C, C-18), 137.2 (1C, C-3), 135.0 (1C, C-2), 129.3 (1C, C-12), 129.0 (2C, C-20 and C-22), 128.9 (2C, C-13 and C-17), 128.1 (1C, C-4), 127.9 (1C, C-21), 126.8 (2C, C-19 and C-23), 126.5 (2C, C-14 and C-16), 59.6 (1C, C-10), 58.5 (1C, C-8), 13.2 (1C, C-7) ppm. Molecular weight Calc. for [C_22_H_22_N_3_O_4_]^+^ = 392.2 and for [C_22_H_22_N_3_O_3_]^+^ = 376.2. Found ESI^+^-MS (MeOH) *m*/*z*: 392.1 and 377.1.

### 4.9. Reagents and Enzymes

Cholinesterase substrate acetylthiocholine (ATCh) and the thiol reagent 5,5′-dithiobis(2-nitrobenzoic acid) (DTNB) for enzyme activity measurement were purchased from Sigma-Aldrich (St. Louis, MO, USA). MTS reagent [3-(4,5-dimethylthiazole-2-yl)-2,5-diphenyl tetrazolium bromide] for cytotoxicity assays was purchased from Promega (Madison, WI, USA).

Paraoxon-ethyl was purchased from Sigma-Aldrich (St. Louis, MO, USA) while tabun and VX were obtained from NC Laboratory (Spiez, Switzerland). Stock solutions of tabun (5000 µg mL^−1^) and VX (5000 µg mL^−1^) were prepared in isopropyl alcohol and further dilutions were made in water just before use. Paraoxon stock (10^−2^ M) was prepared in ethanol and further dilutions were made in water. OPs were used in accordance with the safe use and safe disposition of highly toxic compounds. All nerve warfare agent manipulations, disposal, and experiments were conducted at the Institute for Medical Research and Occupational Health, Zagreb, Croatia, in accordance with the Chemical Weapon Convention, the legislative, and with the approval of the Croatian national authorities and the Organization for the Prohibition of Chemical Weapons, ensuring safe handling and minimizing the risk of hazardous situations [1]. Oxime HI-6 (Sigma-Aldrich, Steinheim, Germany) stock solution was prepared in water and diluted in sodium phosphate buffer or medium just before use. HI-6 is FDA/EUA-approved as an antidote for OP poisonings and as such, it was used in our study as the standard control oxime in all experiments.

The sources of human AChE and BChE were erythrocytes and plasma, respectively, obtained from the blood of a healthy male volunteer and in accordance with the approval of the Ethics Committee of the Institute for Medical Research and Occupational Health. Erythrocytes were isolated from the whole blood and plasma prepared according to a previously publish procedure [52].

Compounds were dissolved in DMSO as 100 mM solutions. Further dilutions were made in water. The final DMSO concentration in the experiments did not exceed 0.8% and did not affect the enzyme activity measurements.

### 4.10. AChE and BChE Reversible Inhibition

The reversible inhibition experiments followed a previously published procedure [29]. The enzyme activity was measured in the presence of a wide range of compound concentrations ensuring 20–80% inhibition of the control enzyme activity. The activity was assayed by Ellman’s method [24]. The assay was performed in 96-well plates on an Infinite M200PRO plate reader (Tecan Austria GmbH, Salzburg, Austria). Each concentration was tested in triplicate or quadruplicate on each plate. The inhibition mixture contained 0.1 mM sodium phosphate buffer pH 7.4, enzyme (AChE or BChE), compound (0.05–3 mM final) and DTNB (0.3 mM final), and ATCh (from 0.05 to 1 mM final) to start the reaction. The measured activity in the presence of compounds was corrected for the non-enzymatic oximolysis of substrate ATCh, if detected [53]. The inhibition dissociation constants *K*_i_ were evaluated from the effect of the substrate concentration on the degree of inhibition according to the Hunter–Downs equation [54] as described previously [22] using the Prism9 software (GraphPad Software, San Diego, CA, USA).

### 4.11. In Silico Predictions

The SwissADME interface [55] was used to determine the in silico physicochemical parameters: molecular weight (M), number of hydrogen bond donors and acceptors (HBDs and HBAs), number of rotating bonds (RBs), lipophilicity (log*P*), and topological polar surface area (TPSA). For all these parameters, recommendations are given based on the existing drugs [30].

Ligands were docked into the enzyme receptors using the Flexible Docking protocol [56], with selected residues used to create side chain conformations. Prior to docking, ligands were created with ChemBio3D Ultra 13.0 (PerkinElmer, Inc., Waltham, MA, USA) and minimized using the CHARMm force field and Smart Minimizer minimization method of the Minimize Ligands protocol implemented in Biovia Discovery Studio Client v18.1. (Dassault Systèmes, Vélizy-Villacoublay, France). The enzyme receptors were prepared starting from the crystal structures of free AChE (PDB ID: 4EY4) [57] and free BChE (PDB ID: 1P0I) [58] with a network of conserved water molecules mapped onto them. The binding site within AChE and BChE was defined as a sphere surrounding the residues that outline the active site gorge, including those that were selected as flexible, thus allowing rotational freedom [59,60,61]. The representative pose of the selected enzyme-dioxime model complex was chosen based on the highest Consensus score calculated from the scoring functions used to estimate the binding affinity, followed by visual inspection to identify common binding motifs for the AChE/BChE ligands reported in the literature. The selected model complexes were analyzed in regards to the dioxime’s binding geometry inside the active site gorge and non-covalent interactions with neighboring amino acid residues. The parameters used for the in silico protocols were configured as reported in previously published work [62].

### 4.12. Reactivation of Phosphylated AChE and BChE with Dioximes 

The reactivation experiments followed a previously published procedure [29]. In general, in the enzyme reactivation assay, plasma as the source of BChE was diluted about 200-fold and erythrocytes as the source of AChE about 400-fold. The undiluted enzymes were incubated with OP for up to 1 h, achieving 95–100% inhibition. The inhibited enzymes were passed through a Strata C18-E column (Phenomenex, Torrance, CA, USA) to remove the excess unconjugated OP. After filtration, the enzyme was incubated with 1 mM dioximes to screen the reactivation potency. At the specified time intervals (up to 24 h), an aliquot was taken and diluted 40-fold in a buffer containing DTNB. The recovered enzyme activity was measured upon addition of the substrate ATCh (1 mM) by Ellman’s method [63]. An equivalent sample of the uninhibited enzyme was passed through a parallel column, diluted to the same extent as the inhibition mixture, and the control activity was also measured in the presence of the same oxime concentration as the reactivation mixture sample [64]. Both the activities of the control and the reactivation mixture were corrected for oximolysis of the substrate ATCh if detected [53]. No spontaneous reactivation occurred. Enzyme activity measurements were performed at 25 °C and 436 (for erythrocyte AChE) and 412 nm (for plasma BChE) on a CARY 300 spectrophotometer with a temperature controller (Varian Inc., Australia). The reactivation screening was performed at a given dioxime concentration wherefrom the observed first-order reactivation rate constant (*k*_obs_) and maximal reactivation percentage (*React*_max_) were determined by the previously described procedure and equation [22]. 

### 4.13. Cytotoxicity Assay

Human neuroblastoma SH-SY5Y (ECACC 94030304) and human embryo kidney HEK293 (ECACC 85120602) were used to evaluate the in vitro cytotoxicity and were obtained from The European Collection of Authenticated Cell Cultures (ECACC). SH-SY5Y were grown in DMEM F12 (Sigma-Aldrich, Steinheim, Germany) supplemented with 15% (*v/v*) fetal bovine serum (FBS, Sigma-Aldrich, Steinheim, Germany), 1% (*v/v*) non-essential amino acids (NEAAs, Sigma-Aldrich, Steinheim, Germany), and 1% (*v/v*) penicillin/streptomycin (PenStrep, Sigma-Aldrich, Steinheim, Germany) while HEK293 were grown in EMEM (Sigma-Aldrich, Steinheim, Germany) supplemented with 10% (*v/v*) FBS (Sigma-Aldrich, Steinheim, Germany), 1% (*v/v*) NEAA (Sigma-Aldrich, Steinheim, Germany), and 1% (*v/v*) PenStrep (Sigma-Aldrich, Steinheim, Germany).

After cultivation, cells were detached using 0.25% Trypsin/EDTA solution (Sigma-Aldrich, St. Louis, MO, USA), re-suspended, and seeded into 96-well plates at a density of 20,000 cells/well 1 day before the experiment. The initial number of 20,000 cells/well was seeded, according to previous experience and literature data [65]. Cells were exposed to the tested compounds for 24 h at a concentration range 6.25–800 µM. After incubation at 37 °C in a 5% CO_2_ atmosphere, cells were washed with phosphate buffer saline and the cytotoxicity profile was determined by measuring the succinate dehydrogenase mitochondrial activity of living cells by the MTS detection reagent assay [66]. The procedure followed a previously described protocol [66] and the manufacturer’s recommendations. The total percentage of DMSO in the cytotoxicity assay was 0.8% and did not influence the cell viability. Data was evaluated from at least two or three experiments (each in duplicate or triplicate) using the IC_50_ nonlinear fit equation predefined in Prism9 software and presented as the percentage of inhibition to control untreated cells.

## 5. Conclusions

In this study, seven new vitamin B6 analogues, pyridioxal dioximes, were synthesized and evaluated as inhibitors and reactivators of human cholinesterases inhibited by several OPs. The most potent inhibitor for AChE was the simplest plain derivative pyridoxal dioxime **1**. Pyridoxal dioxime **7**, with the additional phenyl group, with 3-fold selectivity for BChE, can perhaps be used as a scaffold for the design of new BChE inhibitors. Unfortunately, pyridoxal dioximes possess a limited nucleophilic reactivation potency. However, the tested dioximes were non-toxic to human neuroblastoma (SH-SY5Y) and human embryonal kidney cells (HEK293), indicating that such vitamin B6-based structures could be used for future studies on other systems due to well-known B6 benefits.

## Figures and Tables

**Table 1:** Yield and ratio of the obtained isomers per compound.

Compound	-R	Yield (%)	Ratio of Isomers *
**1**	-H	75	30:70
**2**	-F	43	20:80
**3**	-Cl	68	0:100
**4**	-Br	71	0:100
**5**	-NO_2_	54	20:80
**6**	-2-OCH_3_	53	0:100
**7**	-Ph	76	0:100

* The ratio of the products was determined by integrating the peaks in the proton NMR spectra.

**Table 2:** Reversible inhibition of AChE and BChE by pyridoxal dioximes and their binding selectivity to AChE or BChE (ratio *K*_i_ (AChE) over *K*_i_ (BChE)). Dissociation inhibition constants (*K*_i_ ± standard error) were determined from at least three experiments.

Compound	R-	*K*_i_ ± SE (µM)		*K*_i_ (AChE)/*K*_i_ (BChE)
		AChE	BChE	Selectivity
**1**	4-H	93 ± 13	123 ± 6	0.75
**2**	4-F	340 ± 27	244 ± 124	1.39
**3**	4-Cl	157 ± 34	420 ± 40	0.37
**4**	4-Br	258 ± 46	236 ± 54	1.09
**5**	4-NO_2_	200 ± 13	322 ± 46	0.62
**6**	2-OCH_3_	251 ± 35	150 ± 13	1.67
**7**	4-Ph	380 ± 35	112 ± 24	3.39
**HI-6 ^1^**	/	46 ± 4	420 ± 100	9.13

^1^ From [25].

**Table 3:** Reactivation screening of AChE inhibited by OPs with 1 mM dioximes: first-order reactivation rates, *k*_obs_, and maximal reactivation percentage, *React*_max_ obtained in 24 h. Constants presented as the mean of at least two experiments (standard error was less than 10%, and therefore not indicated).

OP	Compound	R-	*k*_obs_ (min^−1^)	*React*_max_ (%)
	**1**	4-H	0.0006	50
	**2**	4-F	0.0019	65
	**3**	4-Cl	0.0016	55
VX	**4**	4-Br	0.0008	45
	**5**	4-NO_2_	0.0004	25
	**6**	2-OCH_3_	0.0005	30
	**7**	4-Ph	0.0015	60
	**1**	4-H	0.0019	70
	**2**	4-F	0.0011	70
Paraoxon	**3**	4-Cl	0.0029	60
	**4**	4-Br	0.0090	60
	**5**	4-NO_2_	0.0008	20
	**6**	2-OCH_3_	0.0021	75
	**7**	4-Ph	0.0029	75

**Table 4:** Reactivation screening of BChE inhibited by OPs with 1 mM dioximes: first-order reactivation rates, *k*_obs_, and maximal reactivation percentage, *React*_max_ obtained in 24 h. Constants presented as the mean of at least two experiments (standard error was less than 10%, and therefore not indicated).

OP	Compound	R-	*k*_obs_ (min^−1^)	*React*_max_ (%)
	**1**	4-H	0.0022	70
	**2**	4-F	0.0025	70
	**3**	4-Cl	0.0026	50
VX	**4**	4-Br	0.0020	30
	**5**	4-NO_2_	0.0012	60
	**6**	2-OCH_3_	0.0013	65
	**7**	4-Ph	0.0025	90
	**1**	4-H	0.0006	50
	**2**	4-F	0.0011	60
Paraoxon	**3**	4-Cl	0.0010	45
	**4**	4-Br	0.0006	25
	**5**	4-NO_2_	0.0006	35
	**6**	2-OCH_3_	0.0007	50
	**7**	4-Ph	0.0011	70

**Table 5:** Cytotoxicity of the tested compounds on the SH-SY5Y and HEK293 cell lines expressed as IC_50_ values (μM). The experimental data is presented as the mean of at least three experiments.

Compound	R-	IC_50_ (μM)	
		SH-SY5Y	HEK293
**1**	4-H	≥800	≤800
**2**	4-F	≥800	≥800
**3**	4-Cl	≥800	758 ± 10
**4**	4-Br	≥800	≤800
**5**	4-NO_2_	≥800	≥800
**6**	2-OCH_3_	≥800	≥800
**7**	4-Ph	≥800	≥800
**HI-6**	/	≥800	≥800

## Data Availability

Not applicable.

## Supplementary Materials

The following supporting information can be downloaded at: https://www.mdpi.com/article/10.3390/ijms232113388/s1.
