# Supplementary material for: Potential of Vitamin B6 Dioxime Analogues to Act as Cholinesterase Ligands
Source: Int J Mol Sci. 2022 Nov 2;23(21):13388. doi: 10.3390/ijms232113388 (PMC9655973; doi:10.3390/ijms232113388)
Supplement: Supplementary file 1 [file ijms-23-13388-s001.zip › ijms-1919112-supplementary.pdf]

SUPPLEMENTARY INFORMATION

Potential of Vitamin B6 Dioxime Analogues to Act as Cholinesterase Ligands

Dajana Gašo Sokač[1,†], Antonio Zandona[2,†], Sunčica Roca[3], Dražen Vikić-Topić[3,4], Gabriela Lihtar[2], Nikola Maraković[2], Valentina Bušić[1], Zrinka Kovarik[2] and Maja Katalinić[2,*]

NMR spectra of prepared compounds (1 – 7) 2

MS spectra of prepared compounds (1 – 7) 38

Interactions between compound 1 and AChE, and compound 7 and AChE / BChE 45

NMR spectra of prepared compounds (1 – 7)

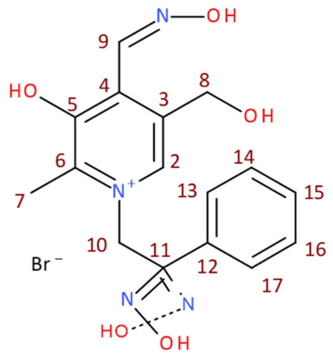

(**1a**) and (**1b**)

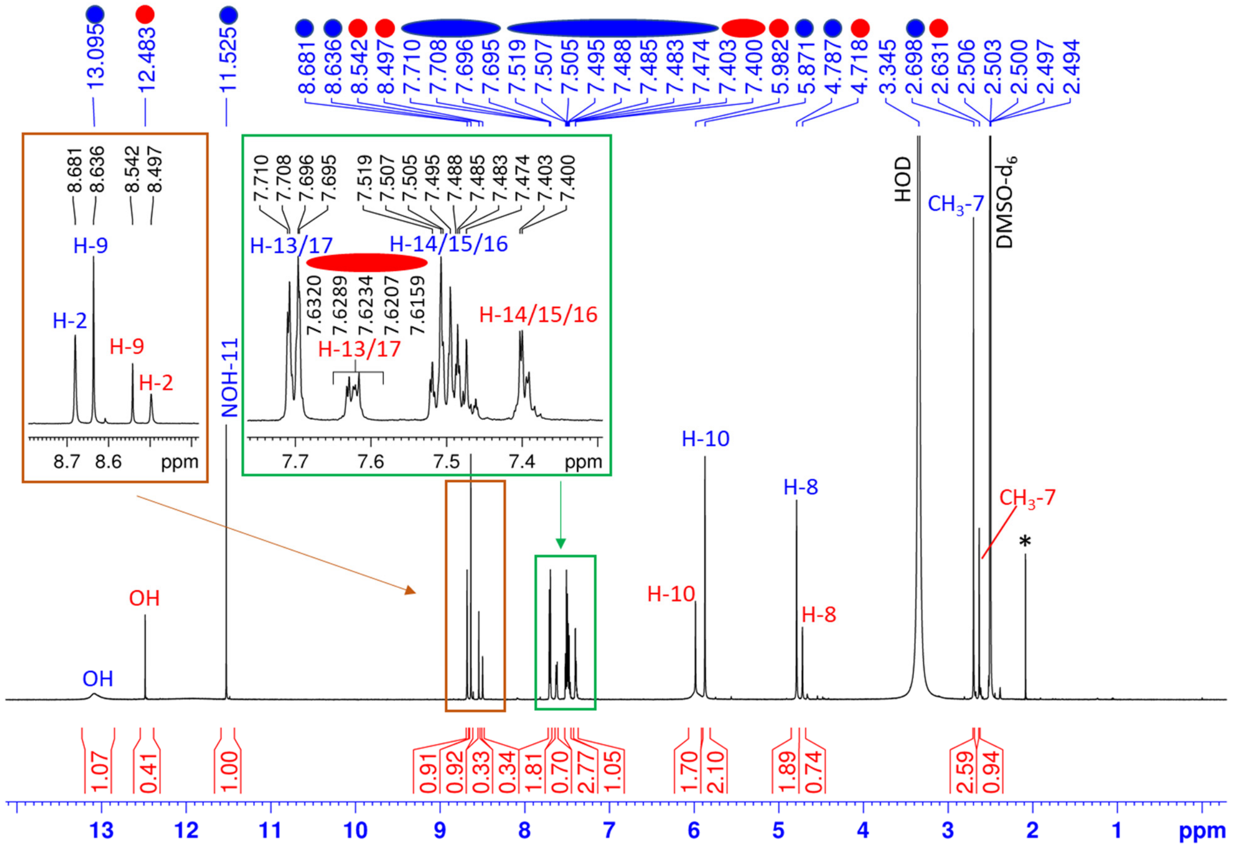

Figure S1. 600 MHz ^{1}H NMR spectrum of the isomer mixture (a and b) of compound **1** in DMSO-d_6. Acetone left after synthesis is marked with an asterisk.

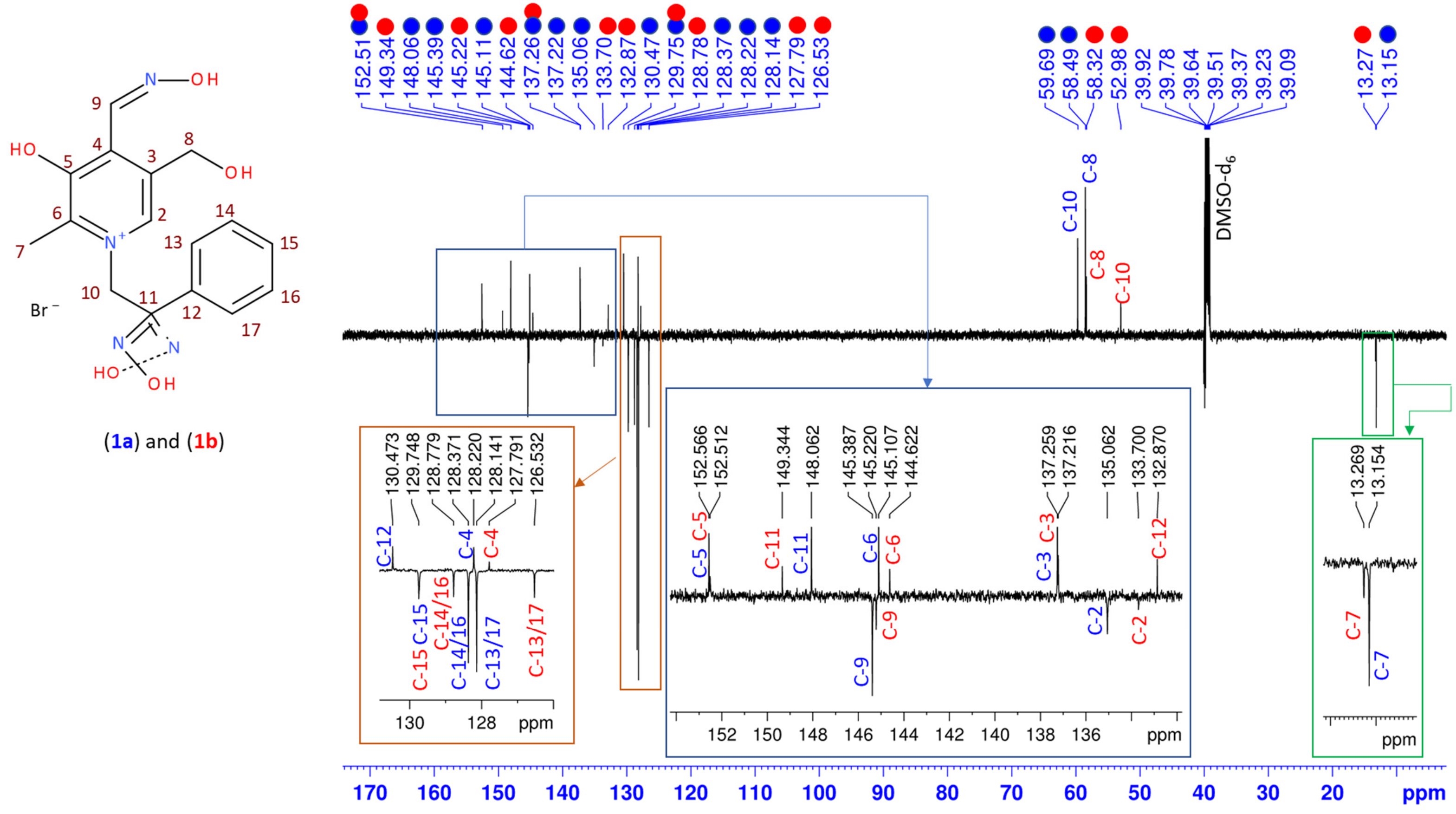

Figure S2. 150 MHz ^{13}C APT spectrum of the isomer mixture (a and b) of compound **1** in DMSO-d_6.

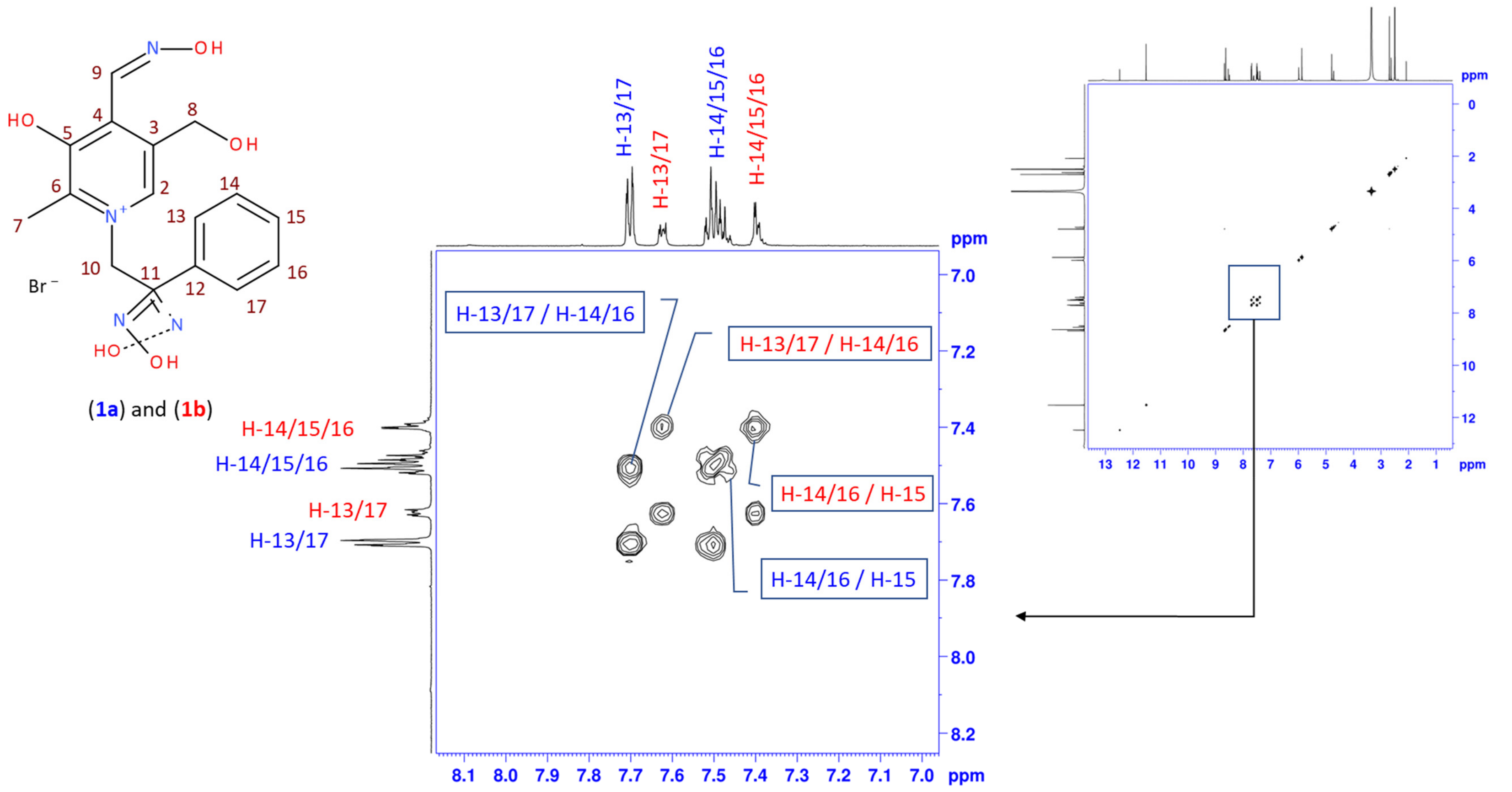

Figure S3. 600 MHz H,H-COSY NMR spectrum of the isomer mixture (a and b) of compound **1** in DMSO-d_6. The one-dimensional ^{1}H spectrum is shown at the top and on the left.

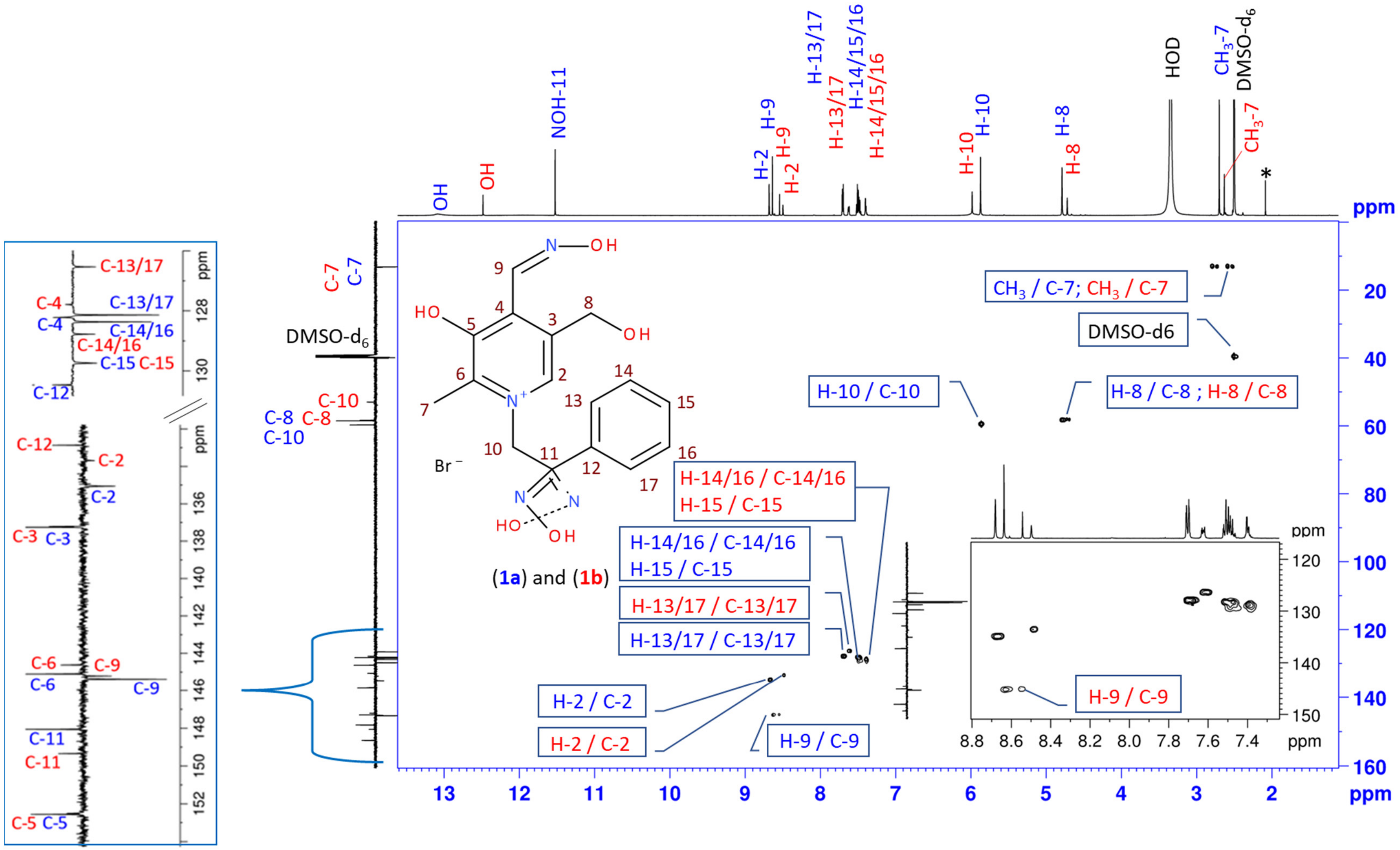

Figure S4. Two-dimensional H,C-correlated spectra of the isomer mixture (a and b) of compound **1** recorded by HMQC method in DMSO-d_6. The 600 MHz ^{1}H spectrum is shown at the top and 125 MHz 13C NMR spectrum at the left-hand edge.

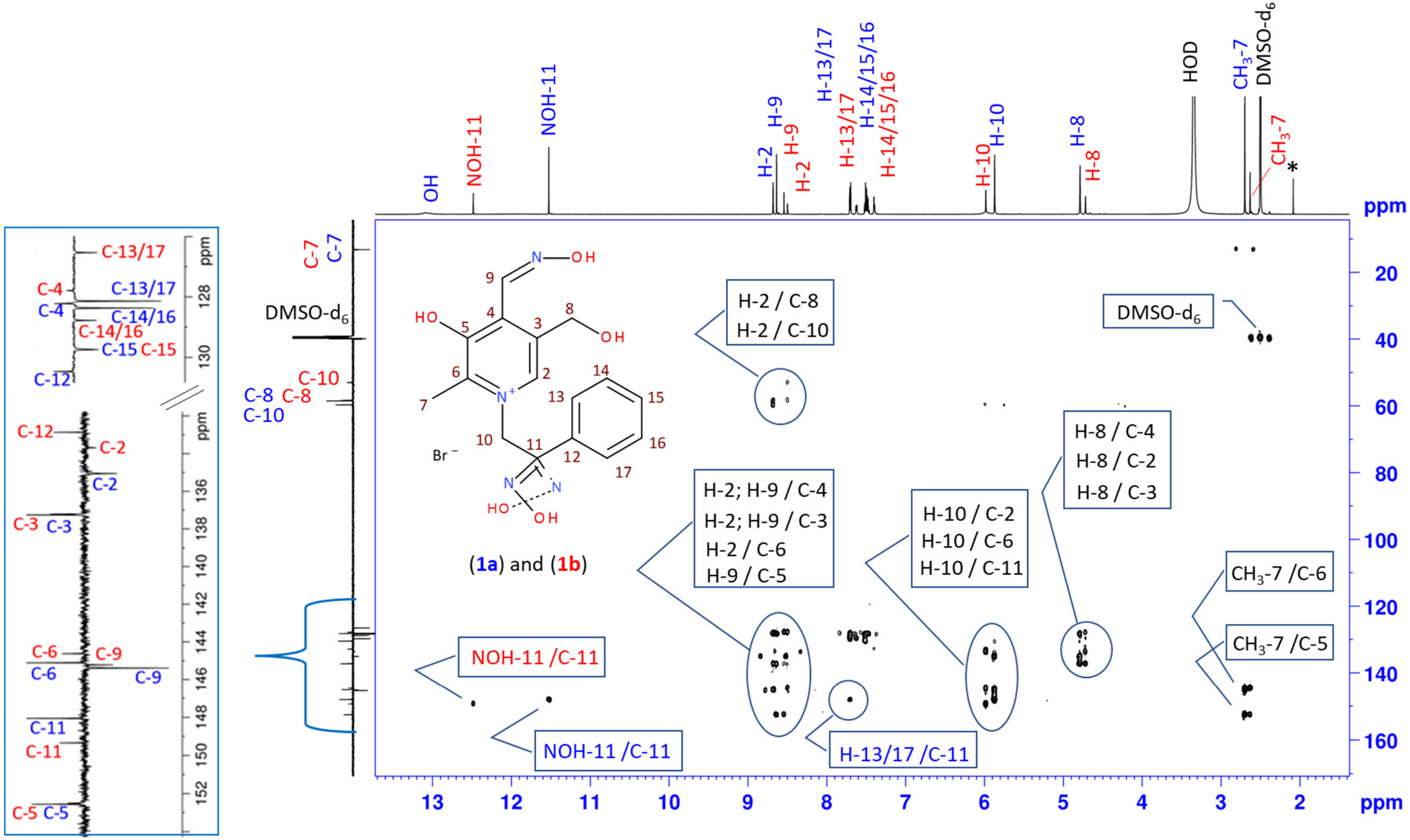

Figure S5. Two-dimensional H,C-correlated spectra of the isomer mixture (a and b) of compound **1** recorded by HMBC method in DMSO-d_6. The 600 MHz ^{1}H spectrum is shown at the top and 125 MHz 13C NMR spectrum at the left-hand edge. Correlation peaks found for both isomers are marked with black letters.

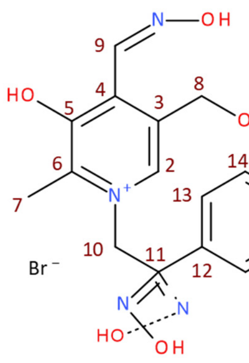

(**1a**) and (**1b**)

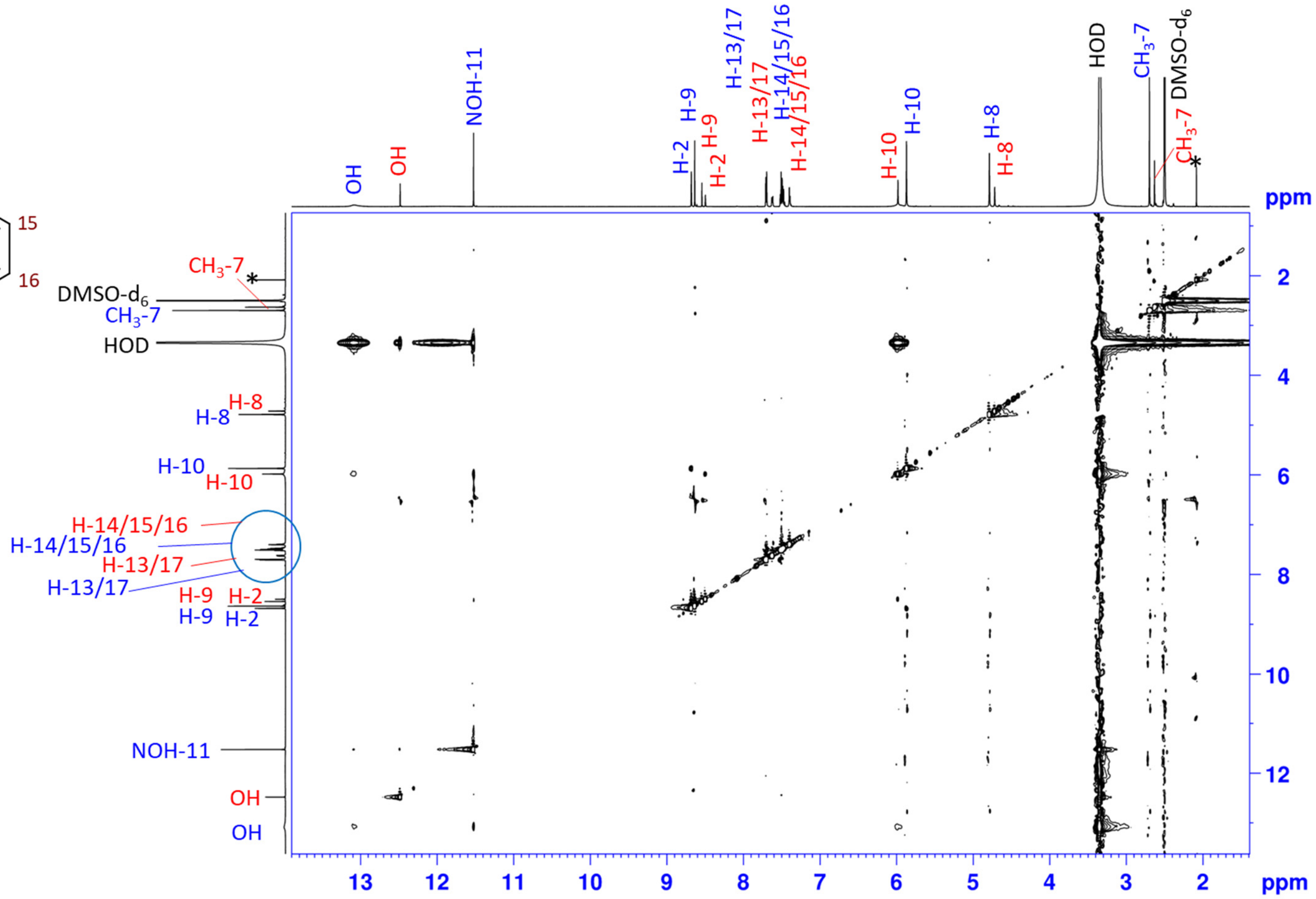

Figure S6. 600 MHz NOESY NMR spectrum of the isomer mixture (a and b) of compound **1** in DMSO-d_6. The one-dimensional ^{1}H spectrum is shown at the top and on the left.

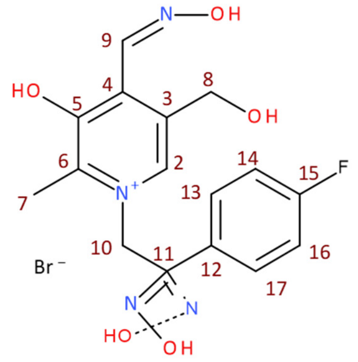

(**2a**) and (**2b**)

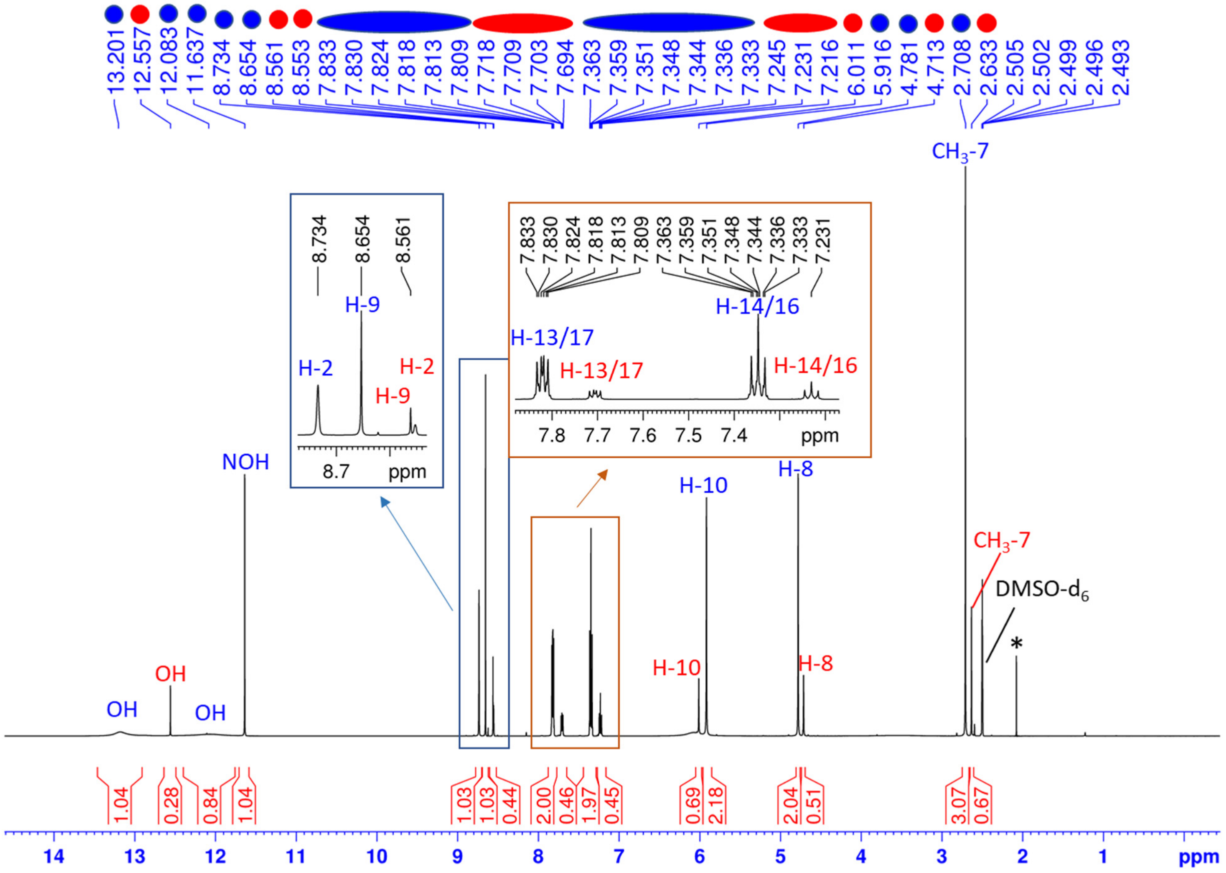

Figure S7. 600 MHz ^{1}H NMR spectrum of the isomer mixture (a and b) of compound **2** in DMSO-d_6. Acetone left after synthesis is marked with an asterisk.

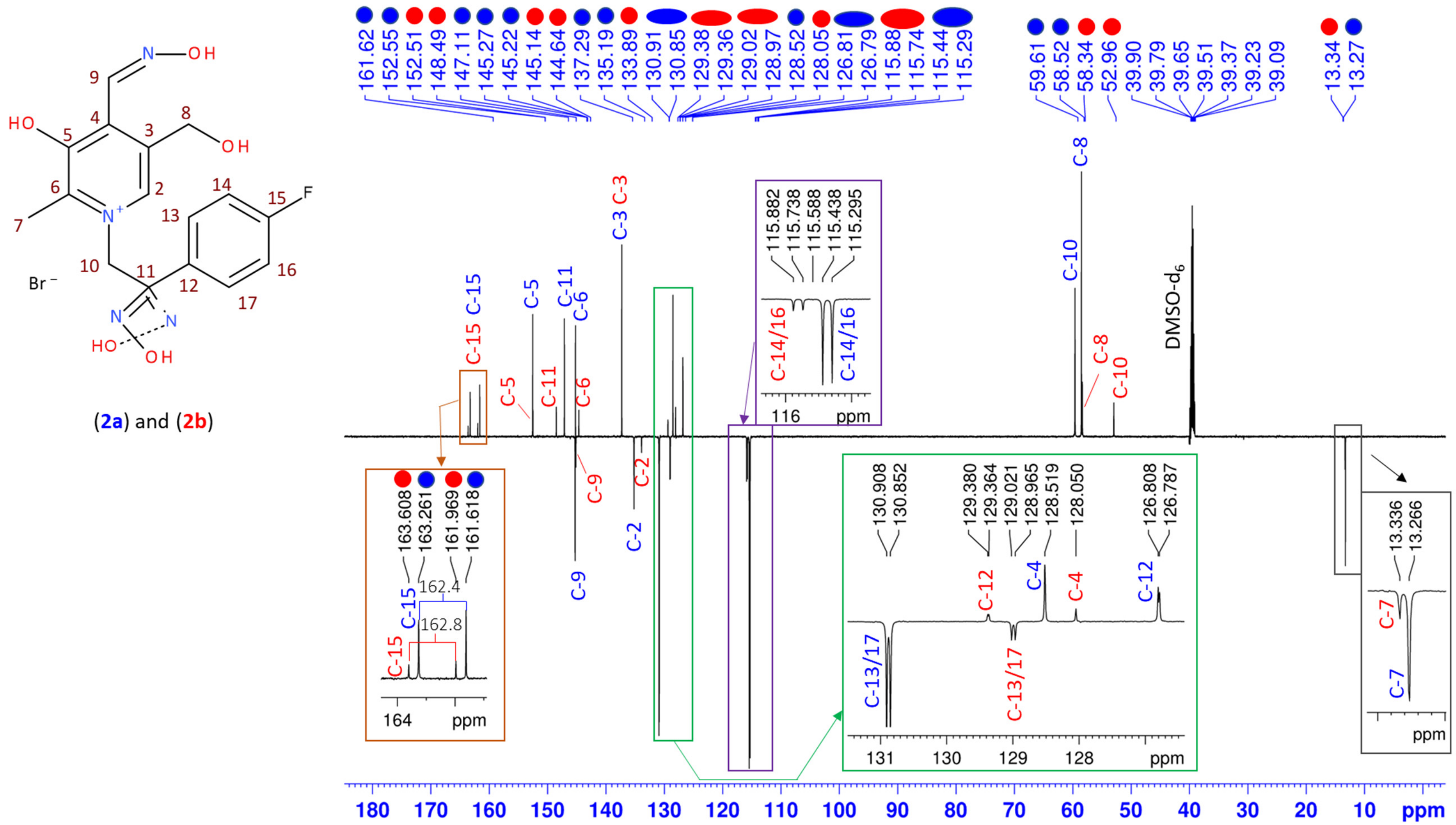

Figure S8. 150 MHz ^{13}C APT spectrum of the isomer mixture (a and b) of compound **2** in DMSO-d_6.

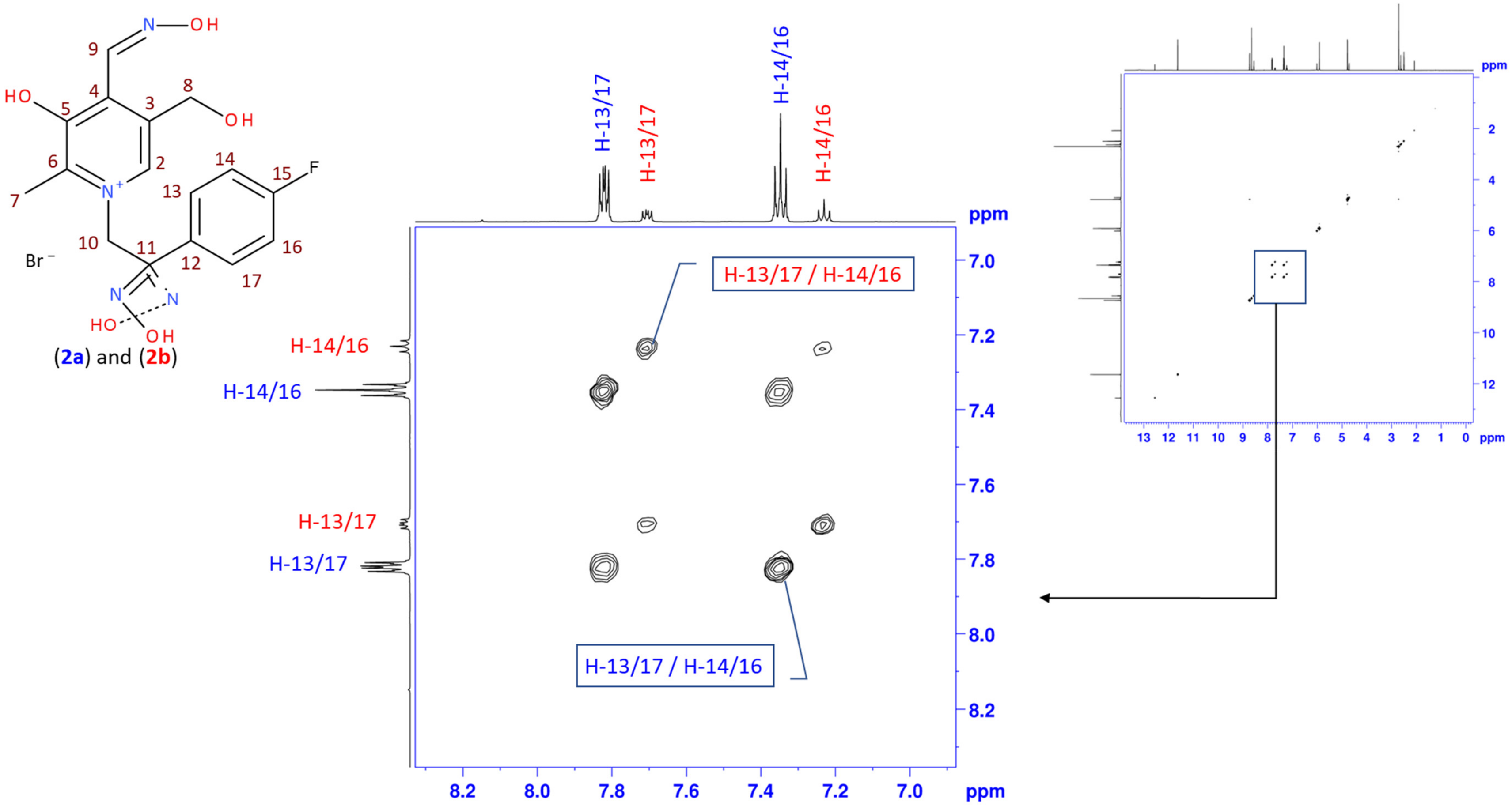

Figure S9. 600 MHz H,H-COSY NMR spectrum of the isomer mixture (a and b) of compound **2** in DMSO-d_6. The one-dimensional ^{1}H spectrum is shown at the top and on the left.

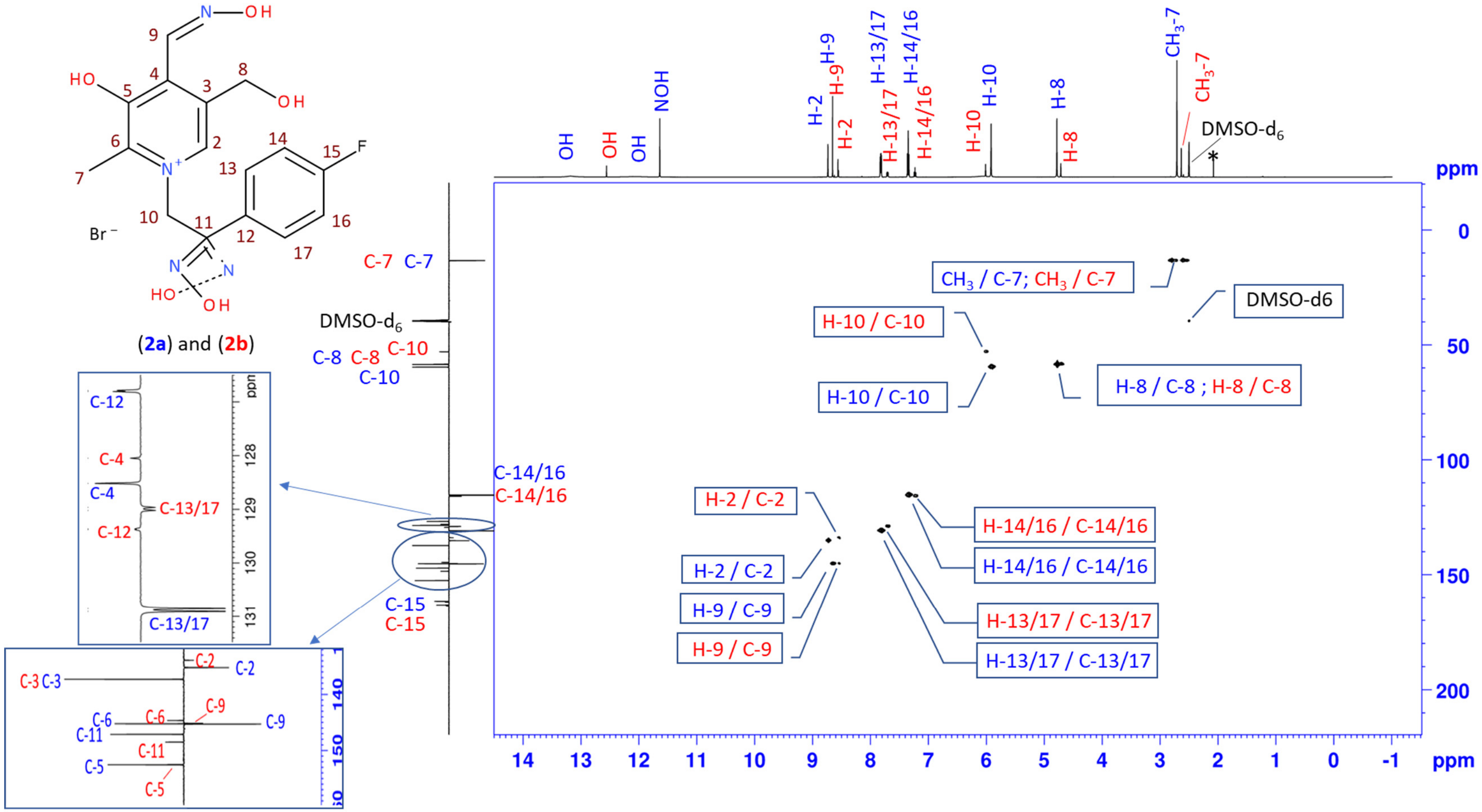

Figure S10. Two-dimensional H,C-correlated spectra of the isomer mixture (a and b) of compound **2** recorded by HMQC method in DMSO-d_6. The 600 MHz ^{1}H spectrum is shown at the top and 125 MHz 13C NMR spectrum at the left-hand edge.

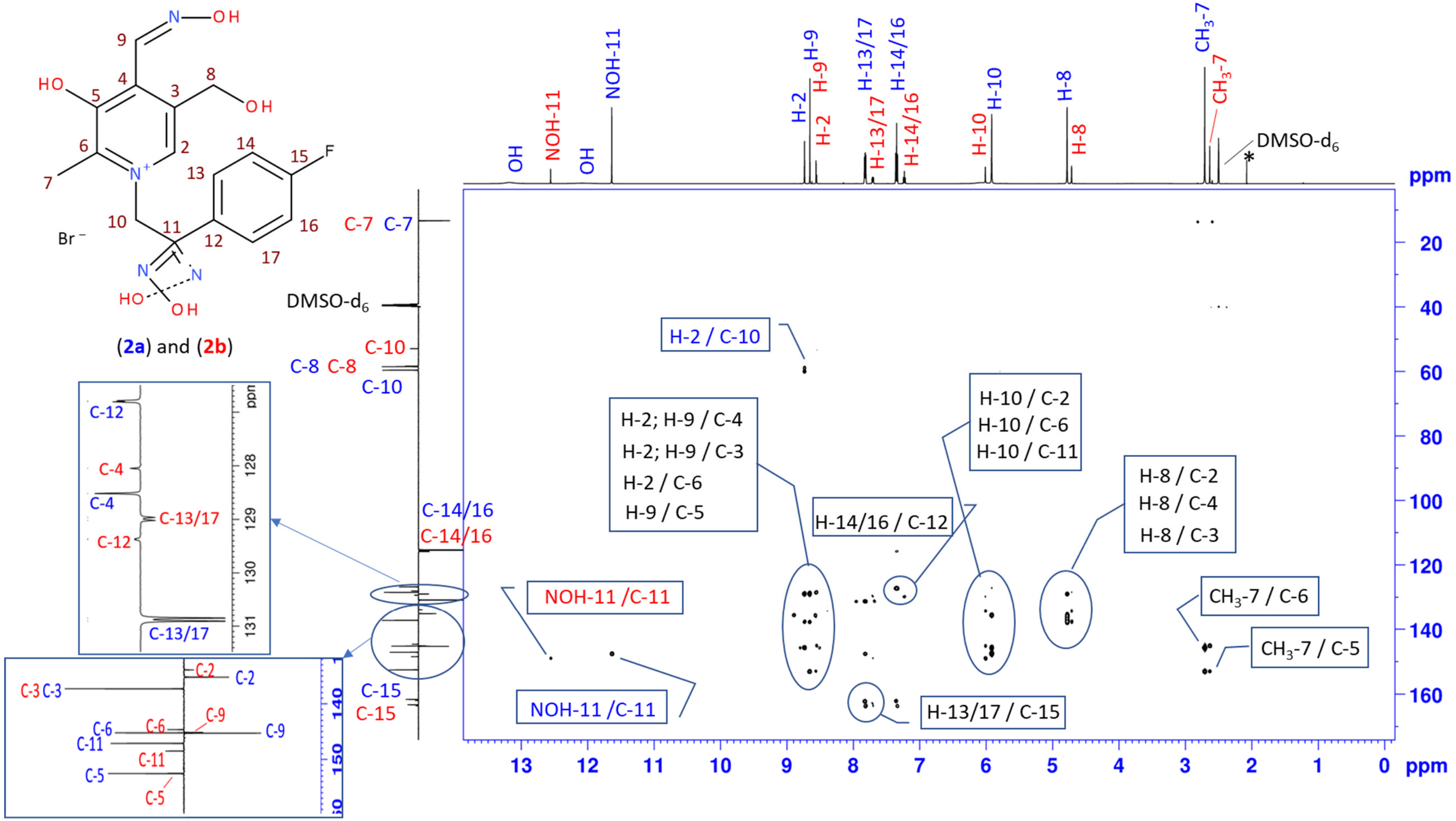

Figure S11. Two-dimensional H,C-correlated spectra of the isomer mixture (a and b) of compound **2** recorded by HMBC method in DMSO-d_6. The 600 MHz ^{1}H spectrum is shown at the top and 125 MHz 13C NMR spectrum at the left-hand edge. Correlation peaks found for both isomers are marked with black letters.

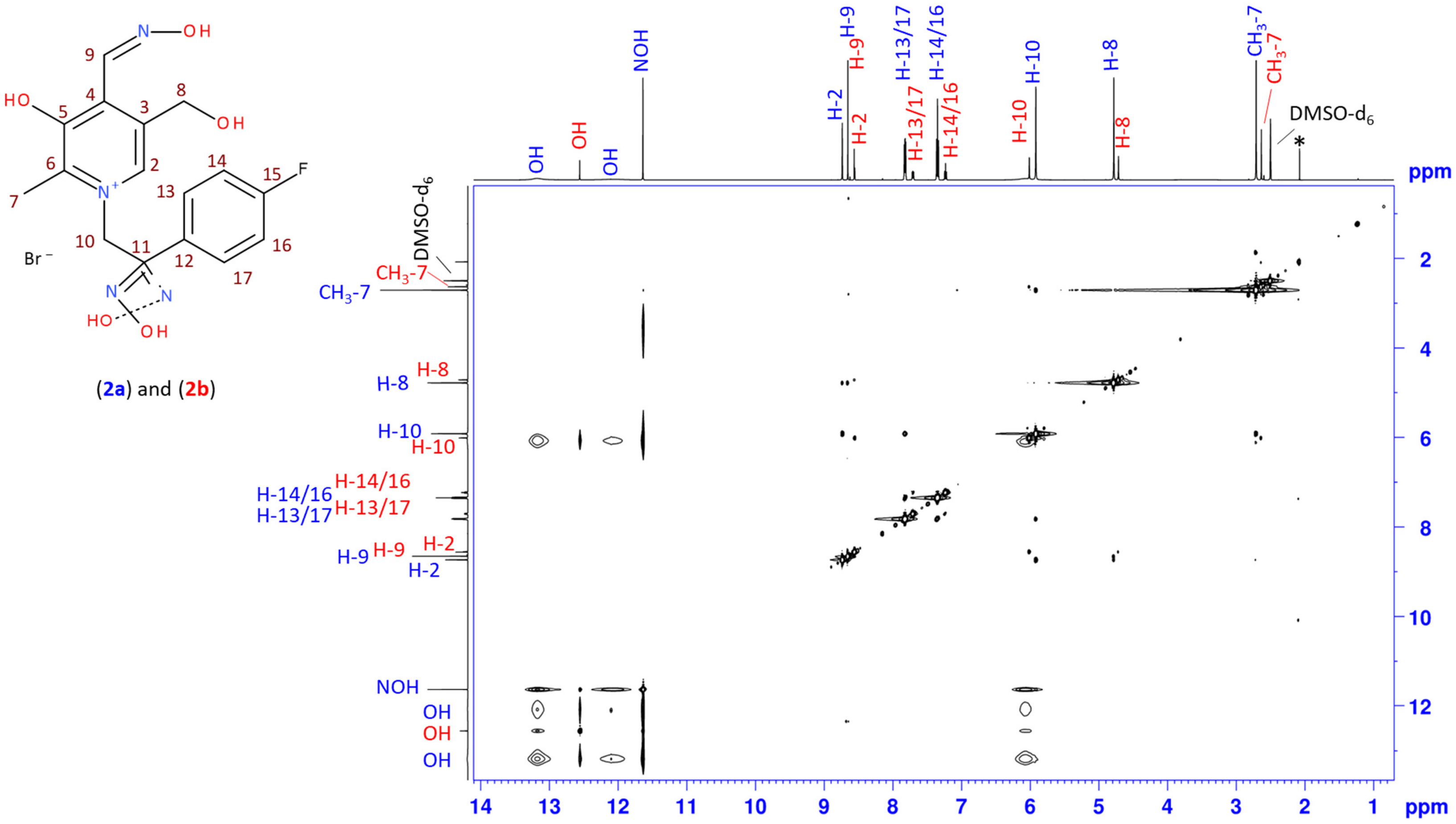

Figure S12. 600 MHz NOESY NMR spectrum of the isomer mixture (a and b) of compound **2** in DMSO-d_6. The one-dimensional ^1H spectrum is shown at the top and on the left.

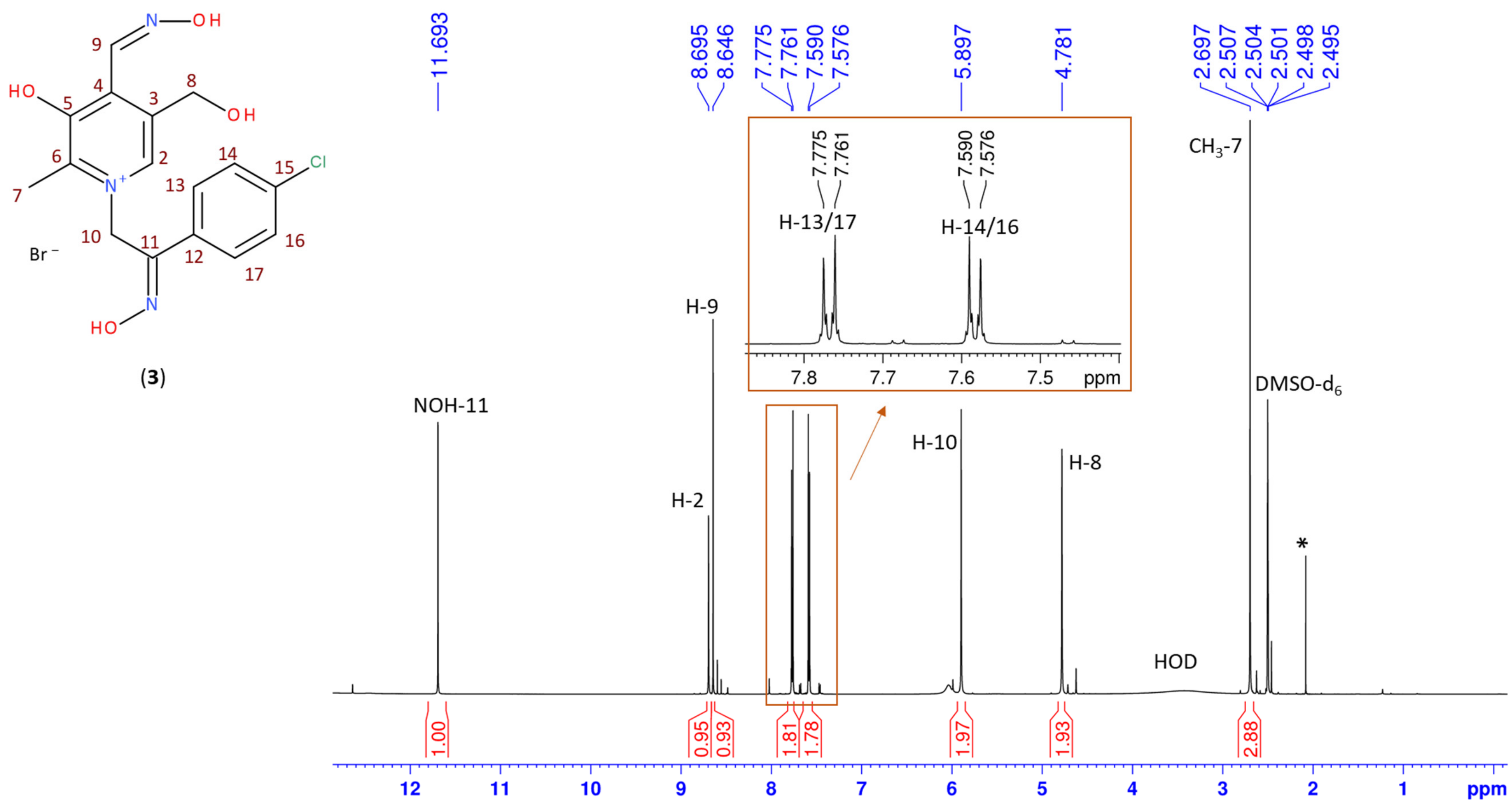

Figure S13. 600 MHz ^{1}H NMR spectrum of compound **3** in DMSO-d_6. Acetone left after synthesis is marked with an asterisk.

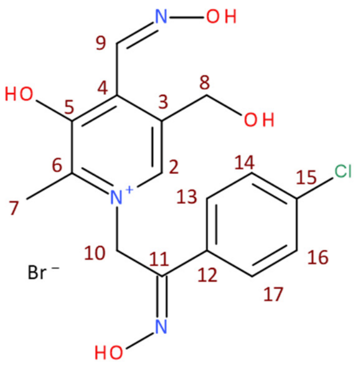

(**3**)

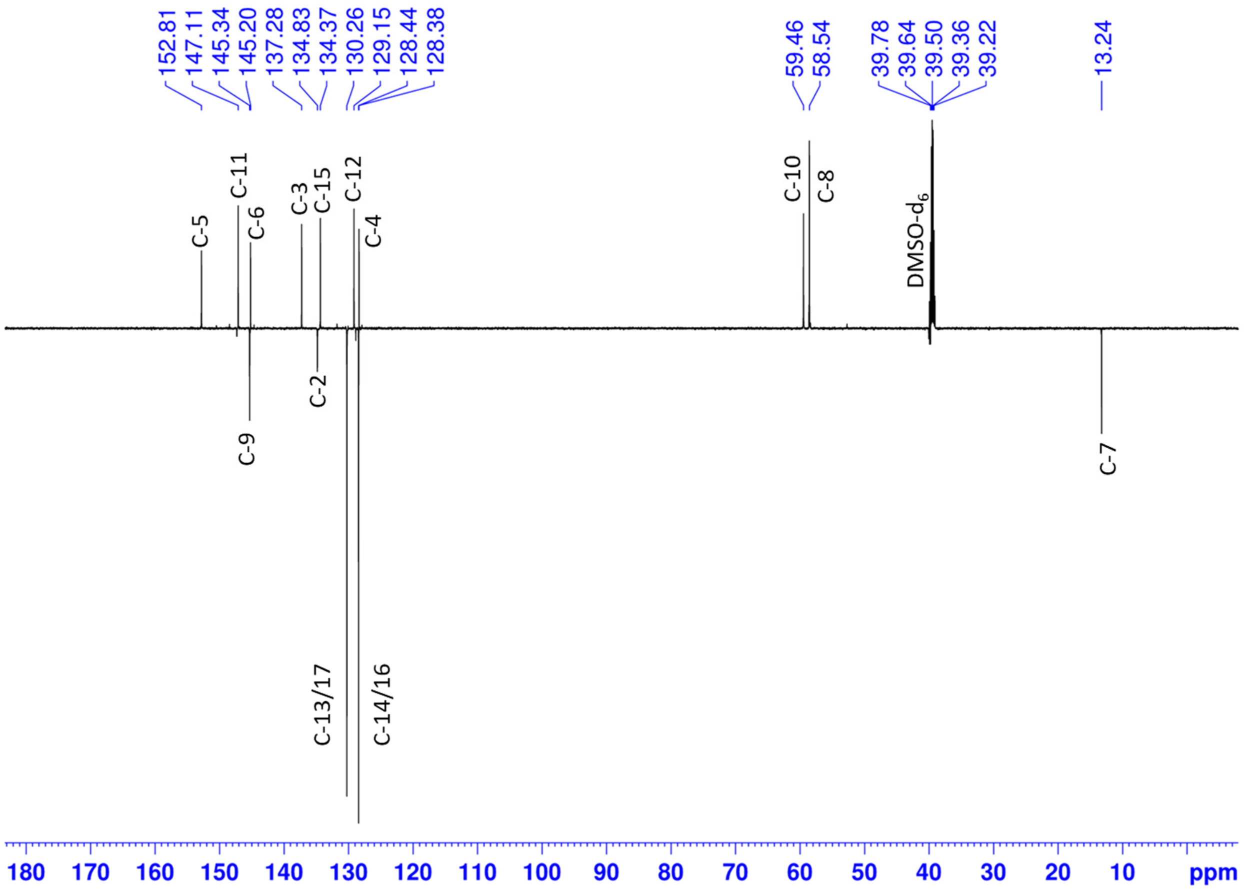

Figure S14. 150 MHz ^{13}C APT spectrum of compound **3** in DMSO-d_6.

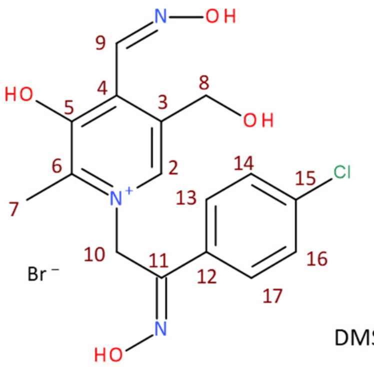

(**3**)

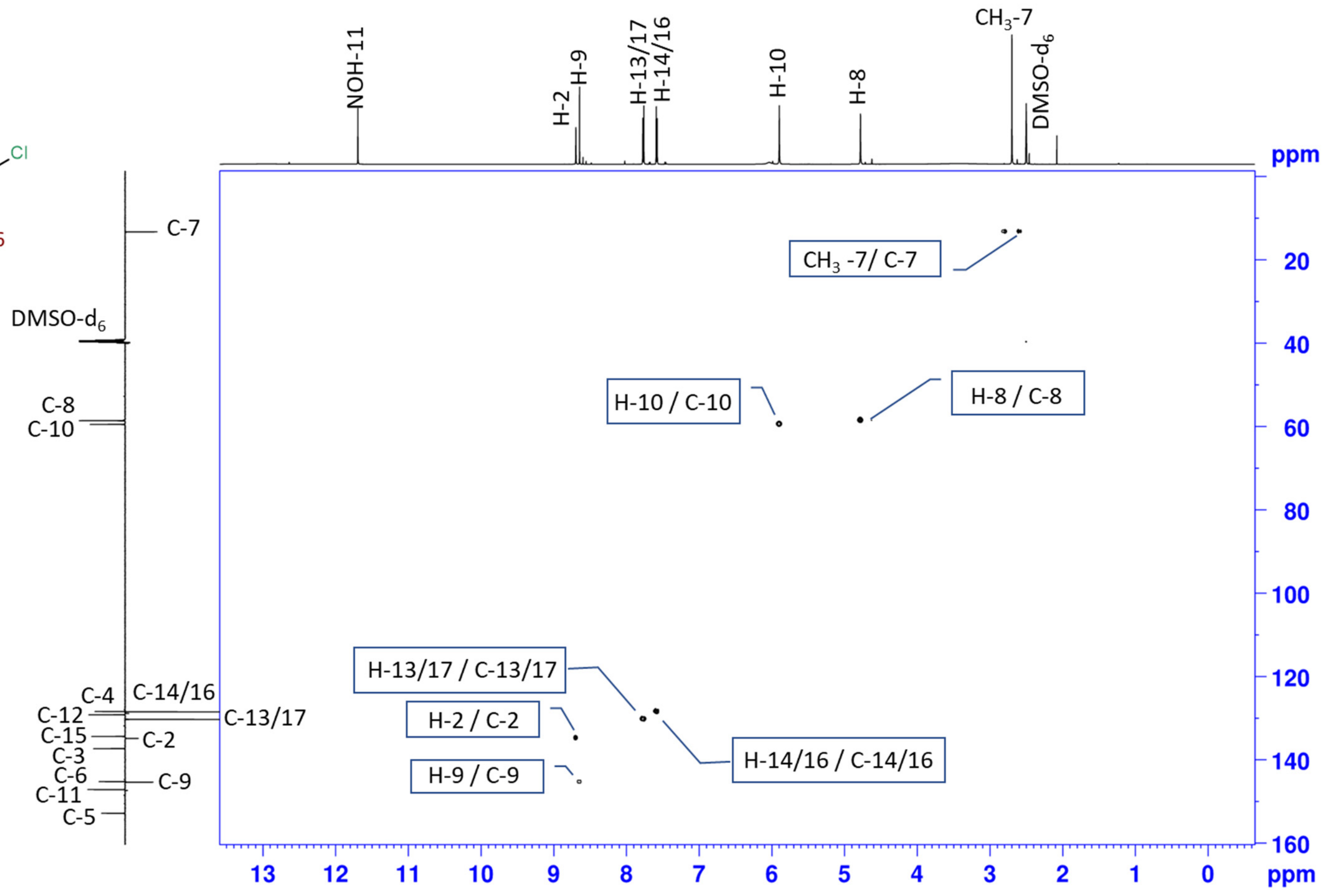

Figure S15. Two-dimensional H,C-correlated spectra of compound **3** recorded by HMQC method in DMSO-d_6. The 600 MHz ^{1}H spectrum is shown at the top and 125 MHz 13C NMR spectrum at the left-hand edge.

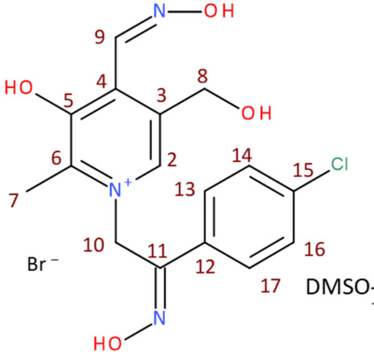

(**3**)

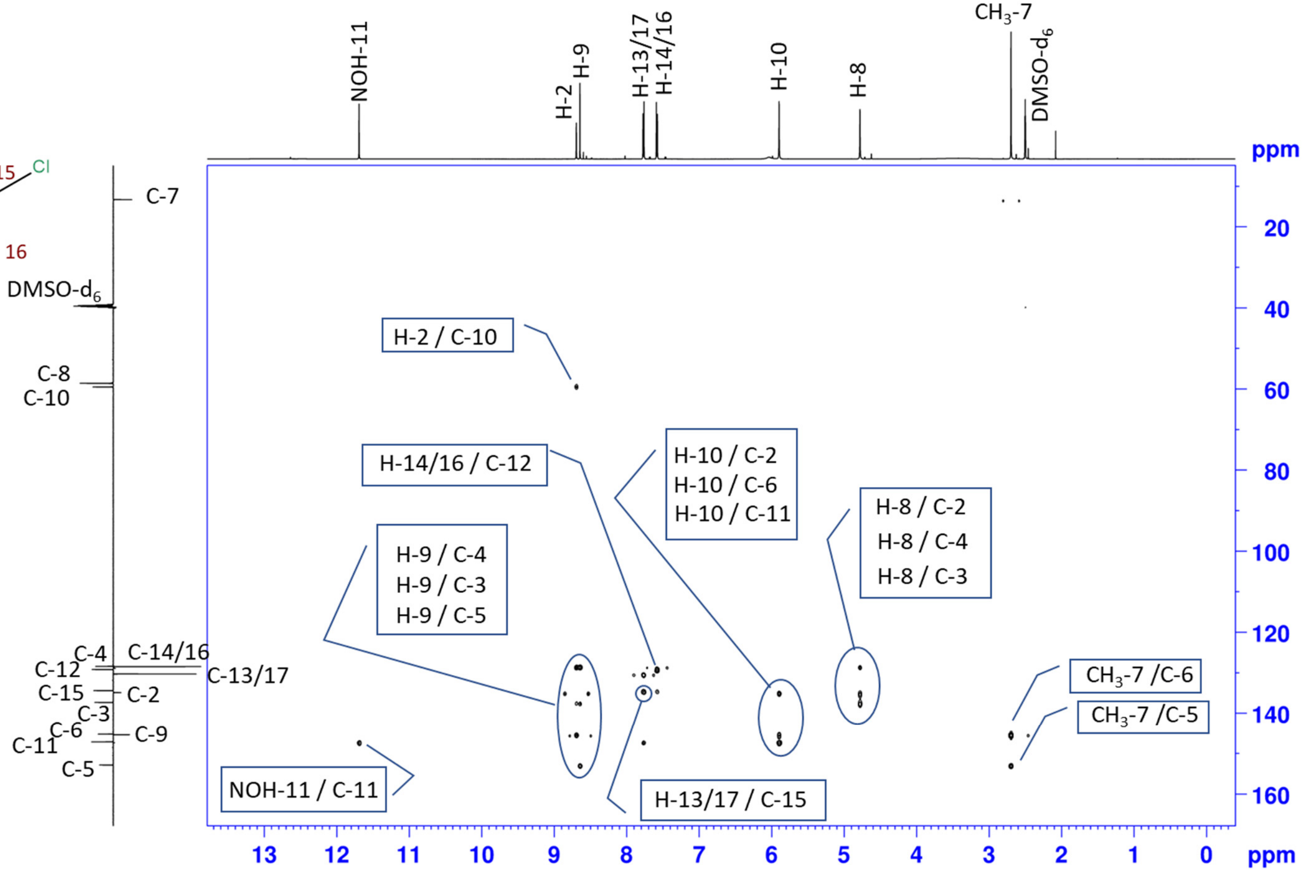

Figure S16. Two-dimensional H,C-correlated spectra of compound **3** recorded by HMBC method in DMSO-d_6. The 600 MHz ^{1}H spectrum is shown at the top and 125 MHz 13C NMR spectrum at the left-hand edge.

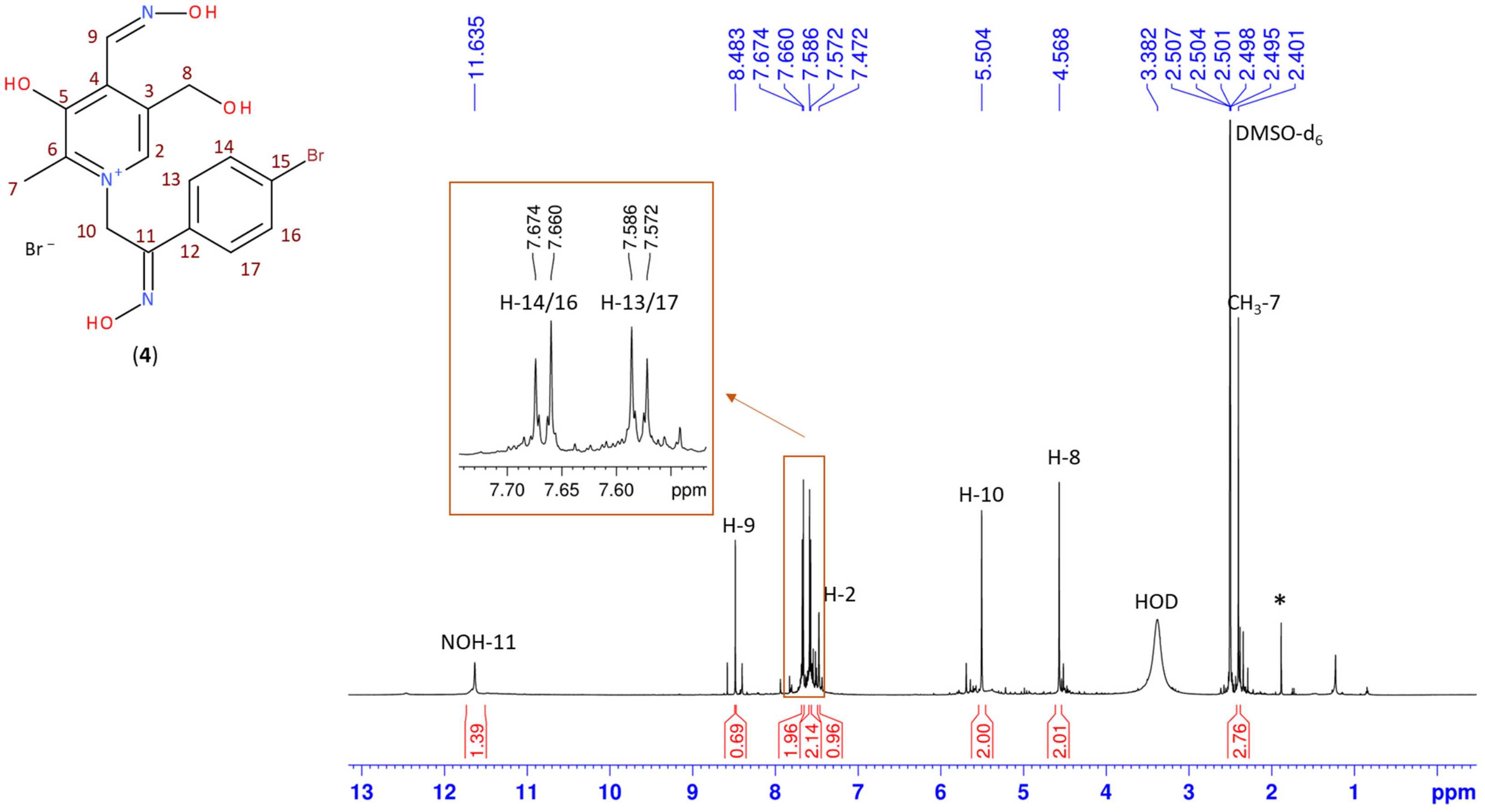

Figure S17. 600 MHz ^{1}H NMR spectrum of compound **4** in DMSO-d_6. Acetone left after synthesis is marked with an asterisk.

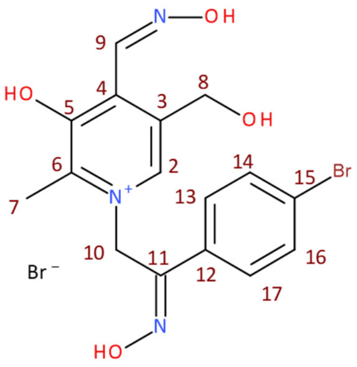

(**4**)

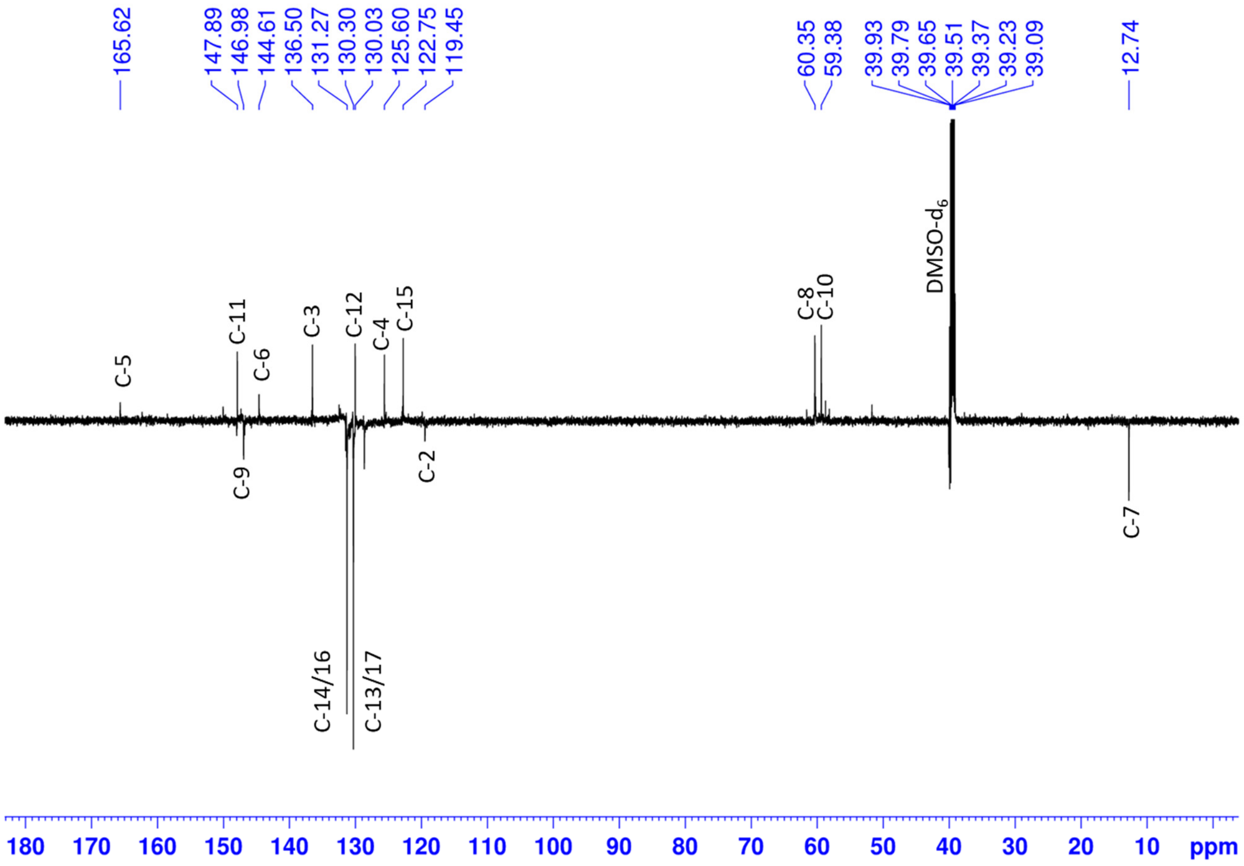

Figure S18. 150 MHz ^{13}C APT spectrum of compound **4** in DMSO-d$_6$.

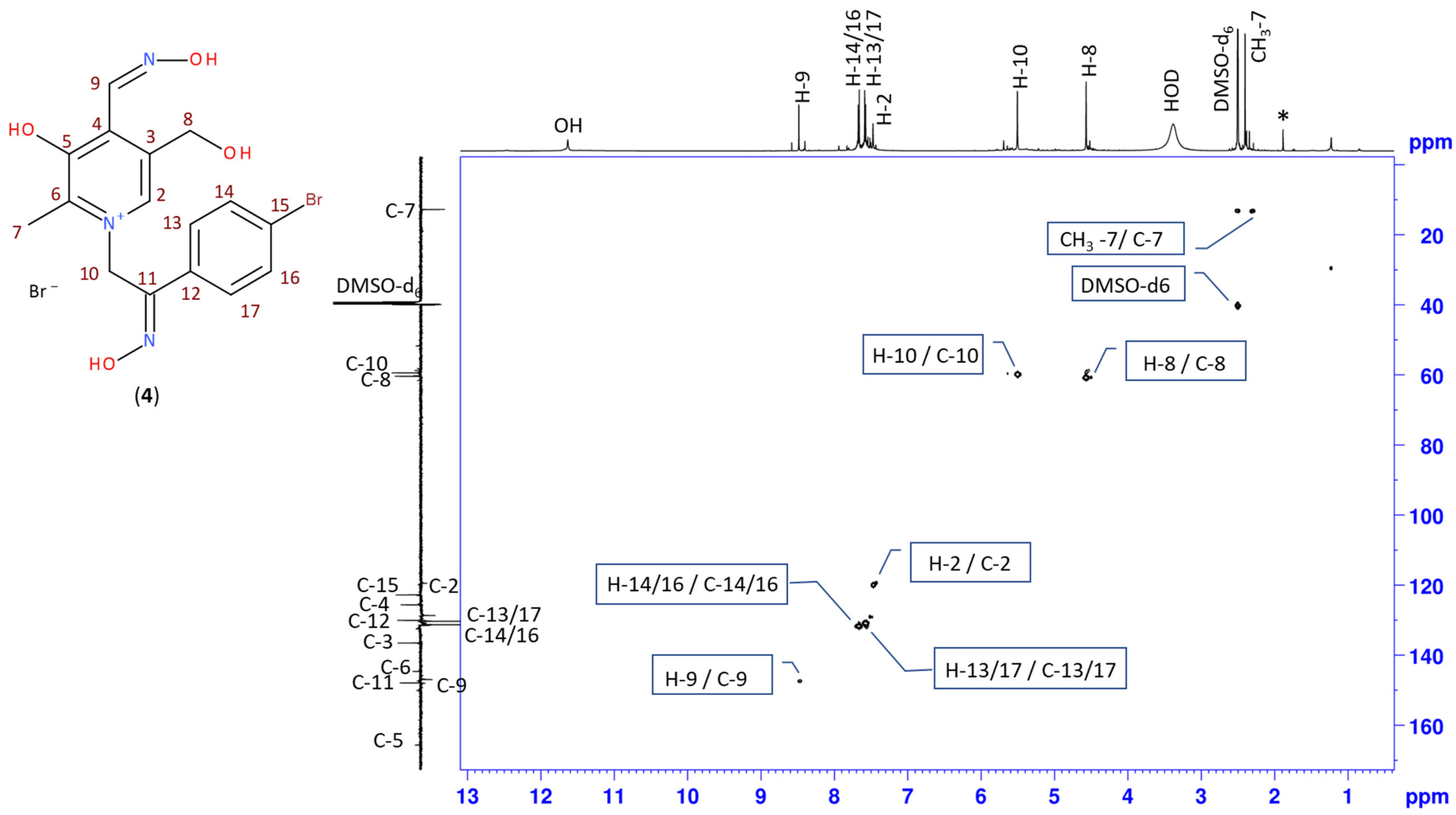

Figure S19. Two-dimensional H,C-correlated spectra of compound **4** recorded by HMQC method in DMSO-d_6. The 600 MHz ^{1}H spectrum is shown at the top and 125 MHz 13C NMR spectrum at the left-hand edge.

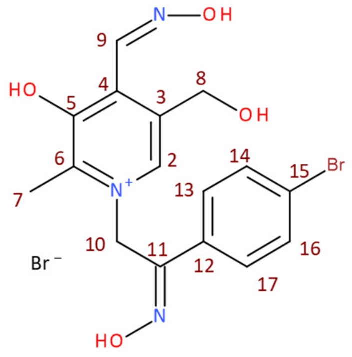

(**4**)

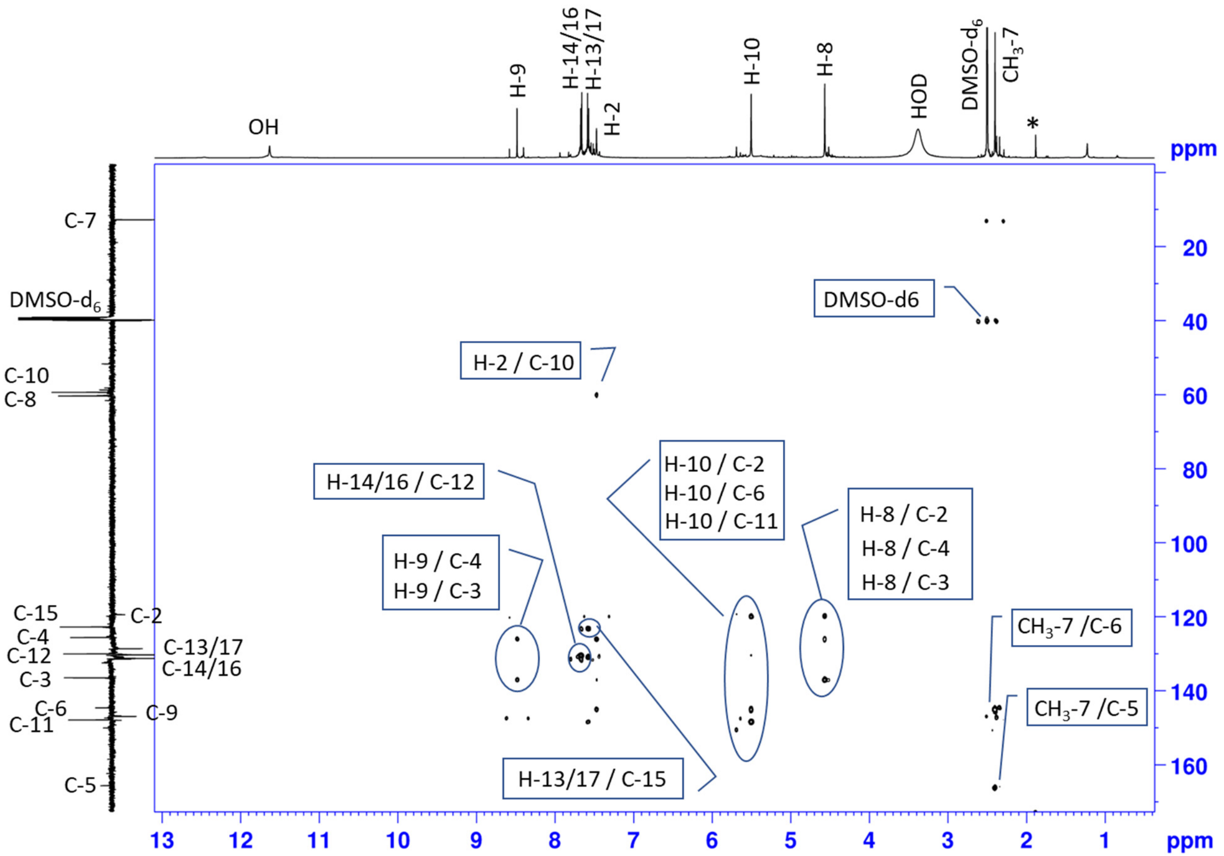

Figure S20. Two-dimensional H,C-correlated spectra of compound **4** recorded by HMBC method in DMSO-d_6. The 600 MHz ^{1}H spectrum is shown at the top and 125 MHz 13C NMR spectrum at the left-hand edge.

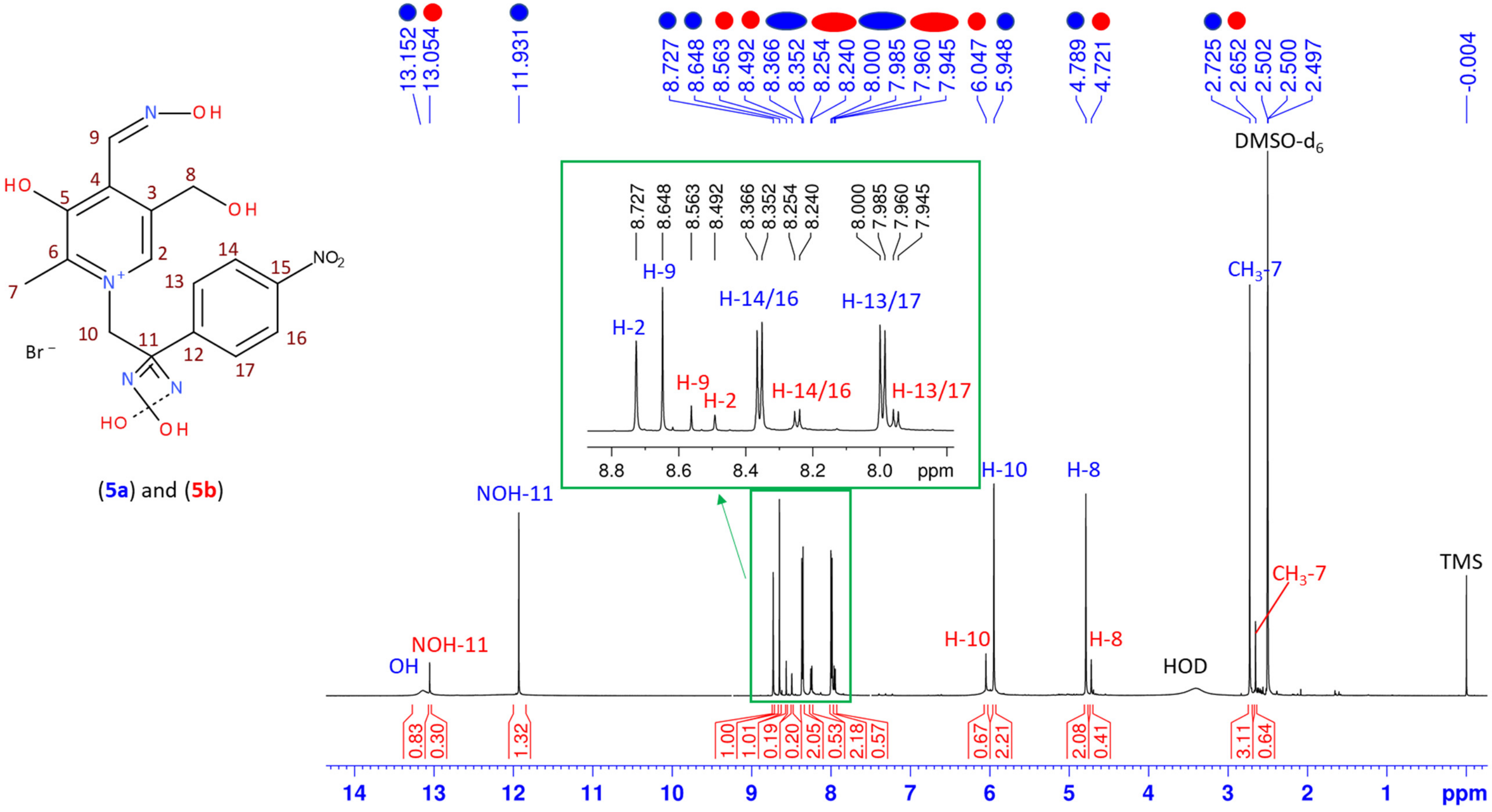

Figure S21. 600 MHz ^{1}H NMR spectrum of the isomer mixture (a and b) of compound **5** in DMSO-d_6. Acetone left after synthesis is marked with an asterisk.

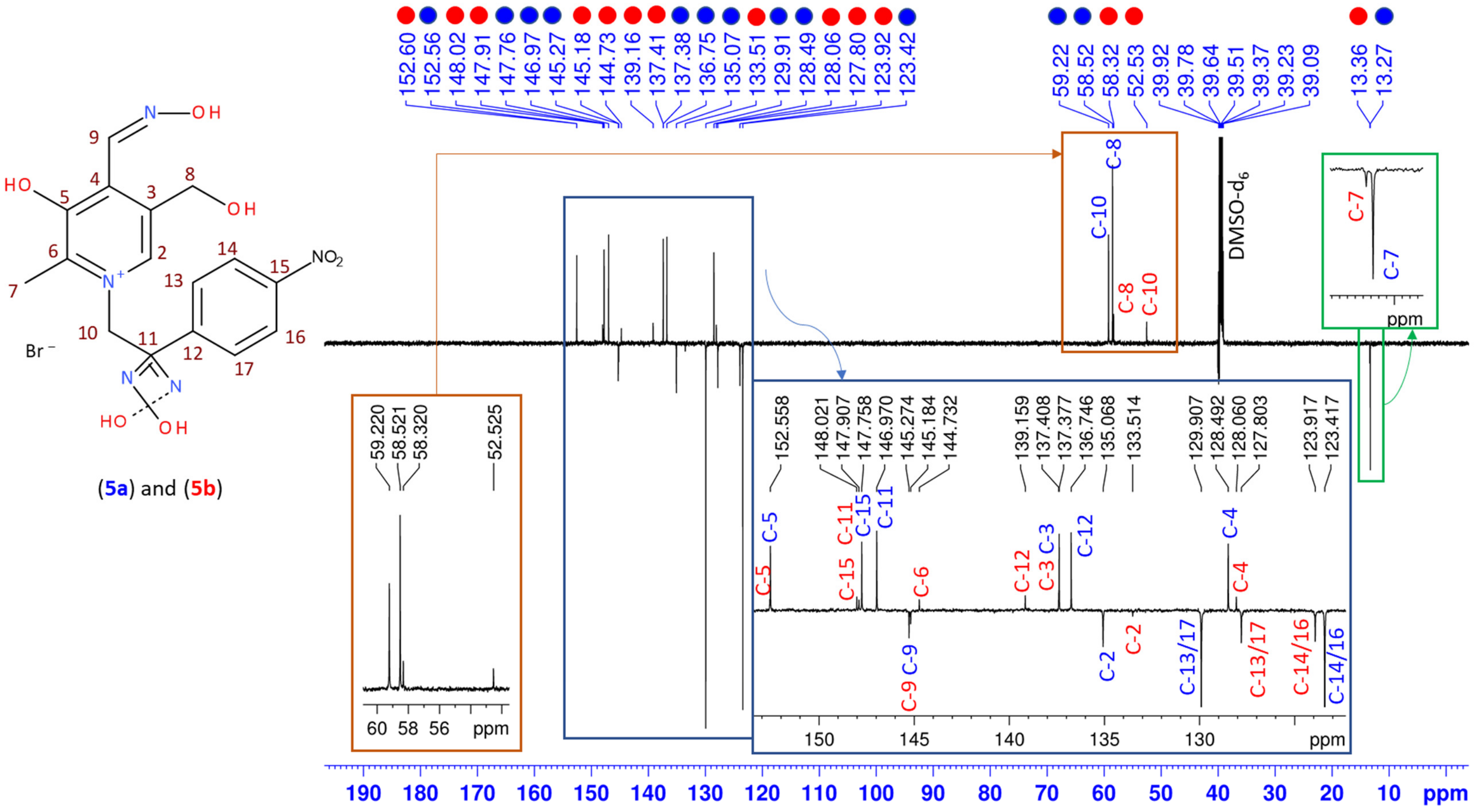

Figure S22. 150 MHz ^{13}C APT spectrum of the isomer mixture (a and b) of compound **5** in DMSO-d$_6$.

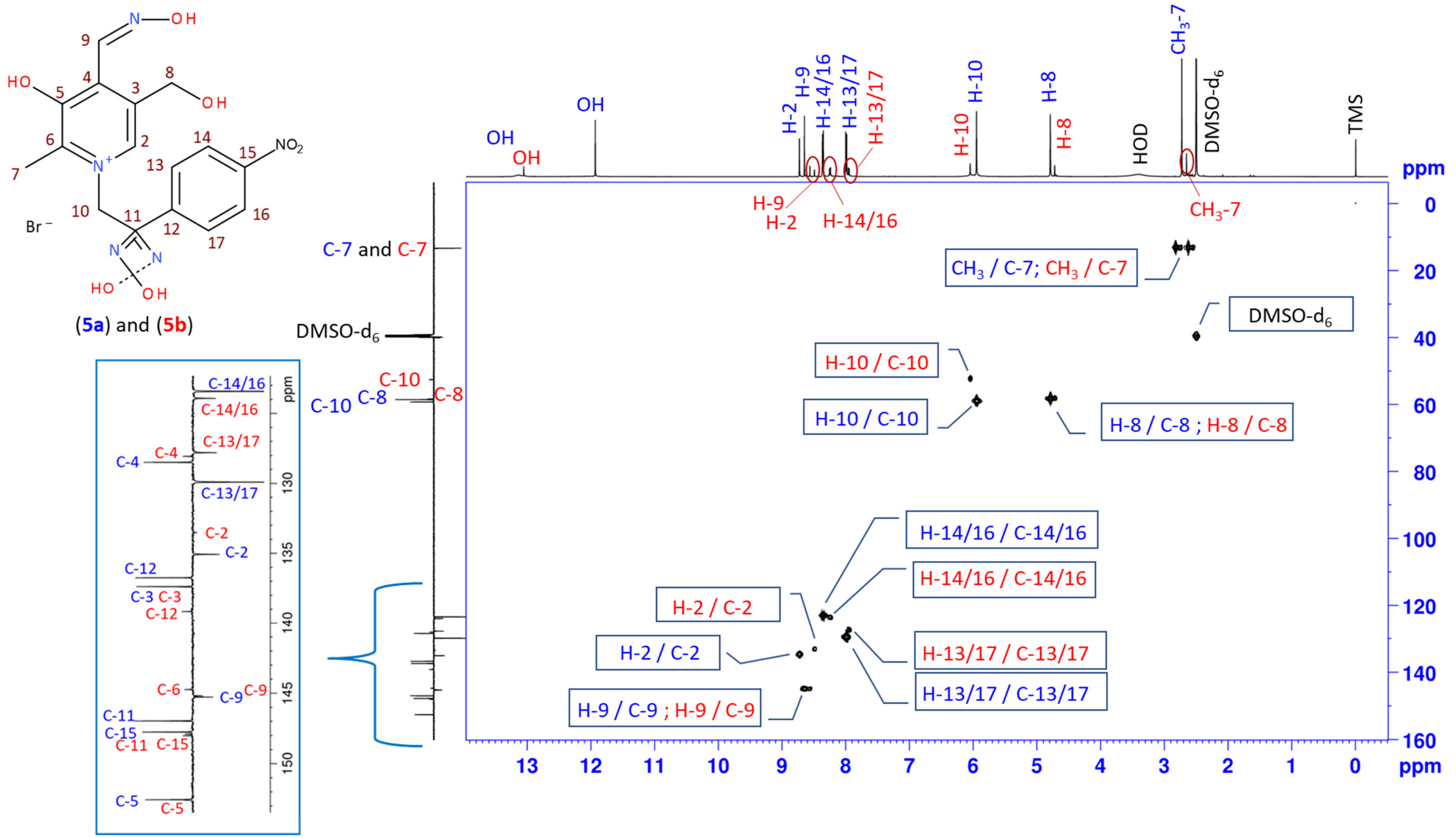

Figure S23. Two-dimensional H,C-correlated spectra of the isomer mixture (a and b) of compound **5** recorded by HMQC method in DMSO-d$_6$. The 600 MHz ^{1}H spectrum is shown at the top and 125 MHz 13C NMR spectrum at the left-hand edge.

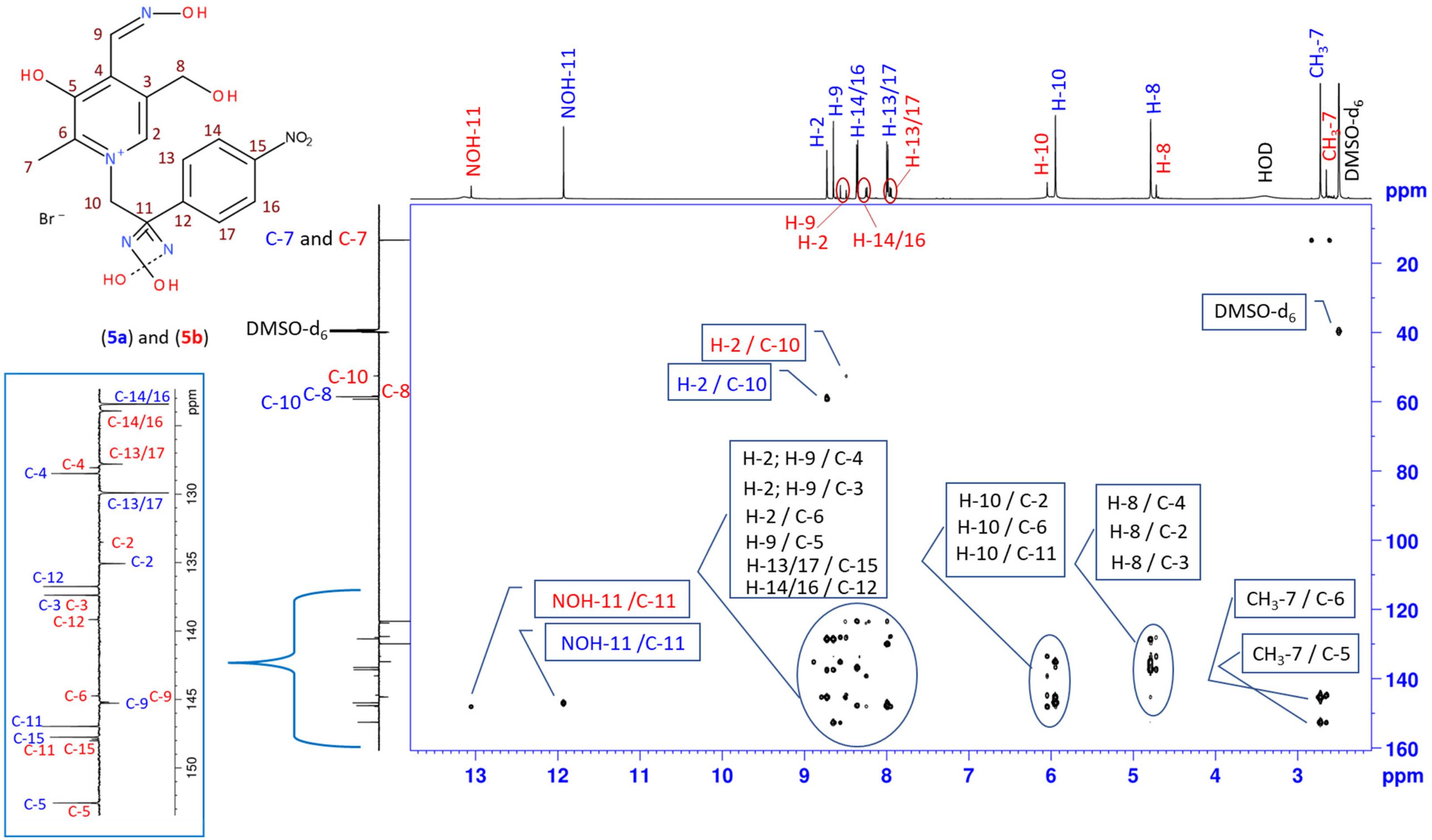

Figure S24. Two-dimensional H,C-correlated spectra of the isomer mixture (a and b) of compound **5** recorded by HMBC method in DMSO-d_6. The 600 MHz ^{1}H spectrum is shown at the top and 125 MHz 13C NMR spectrum at the left-hand edge.

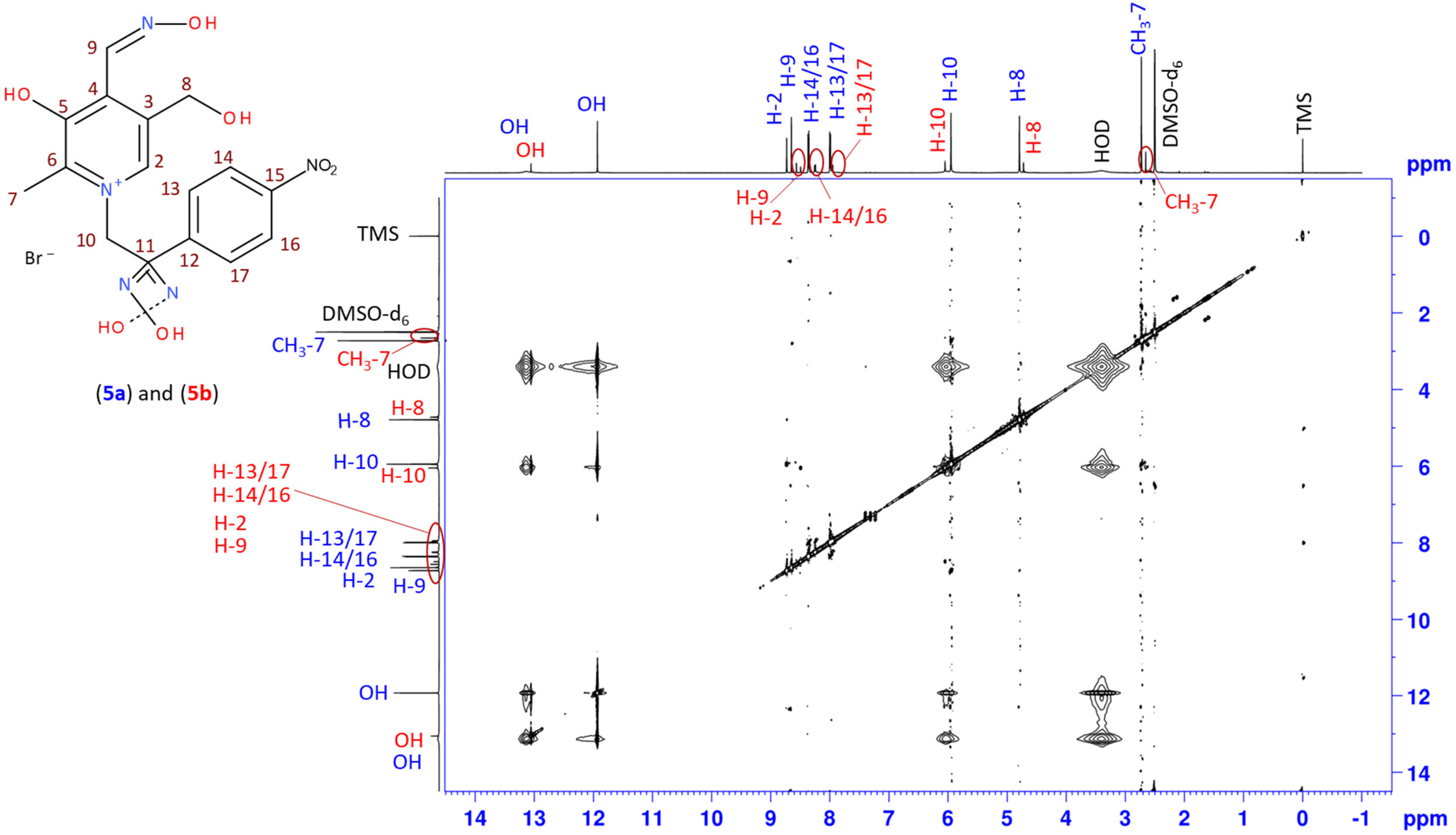

Figure S25. 600 MHz NOESY NMR spectrum of the isomer mixture (a and b) of compound **5** in DMSO-d_6. The one-dimensional ^{1}H spectrum is shown at the top and on the left.

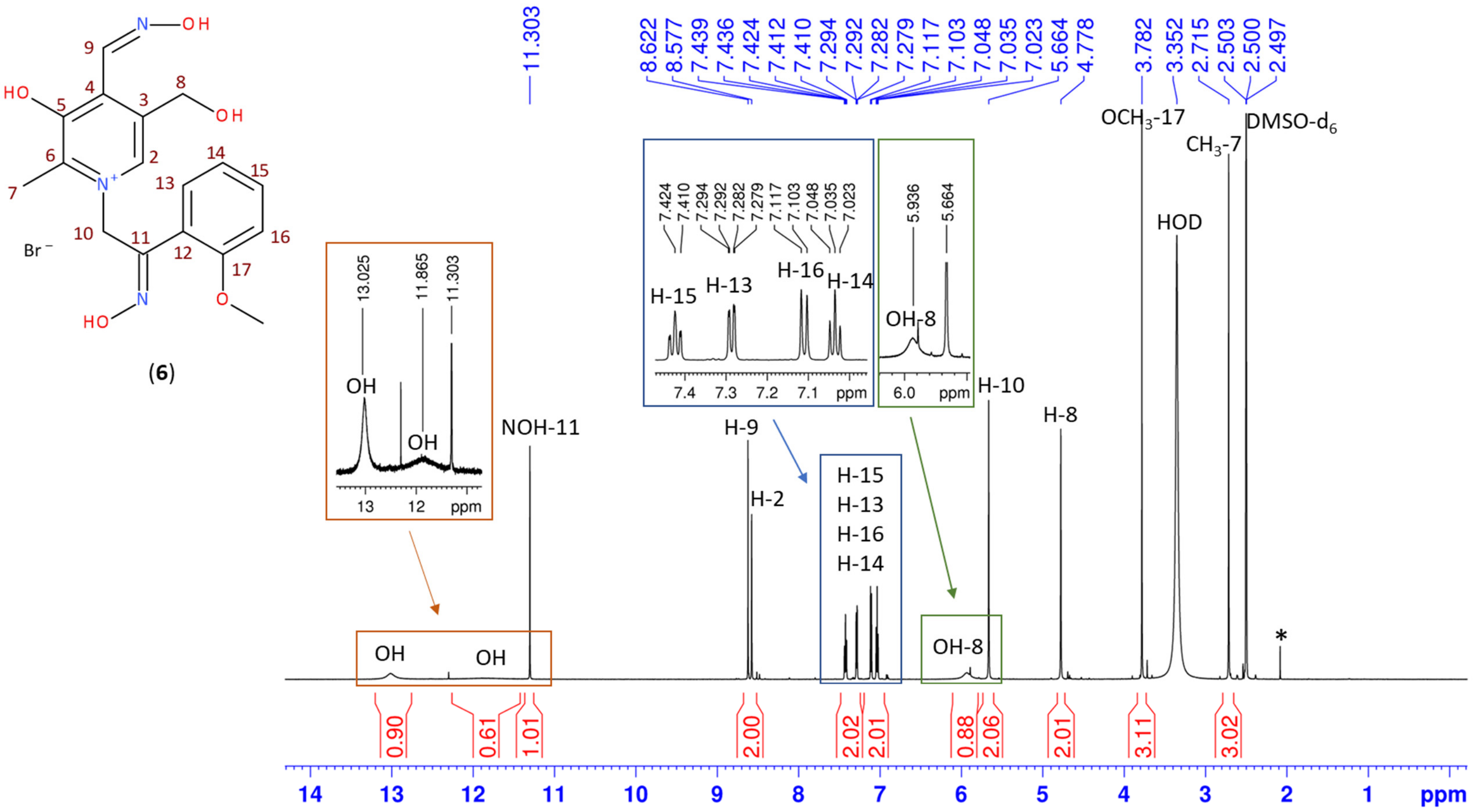

Figure S26. 600 MHz ^{1}H NMR spectrum of compound **6** in DMSO-d_6. Acetone left after synthesis is marked with an asterisk.

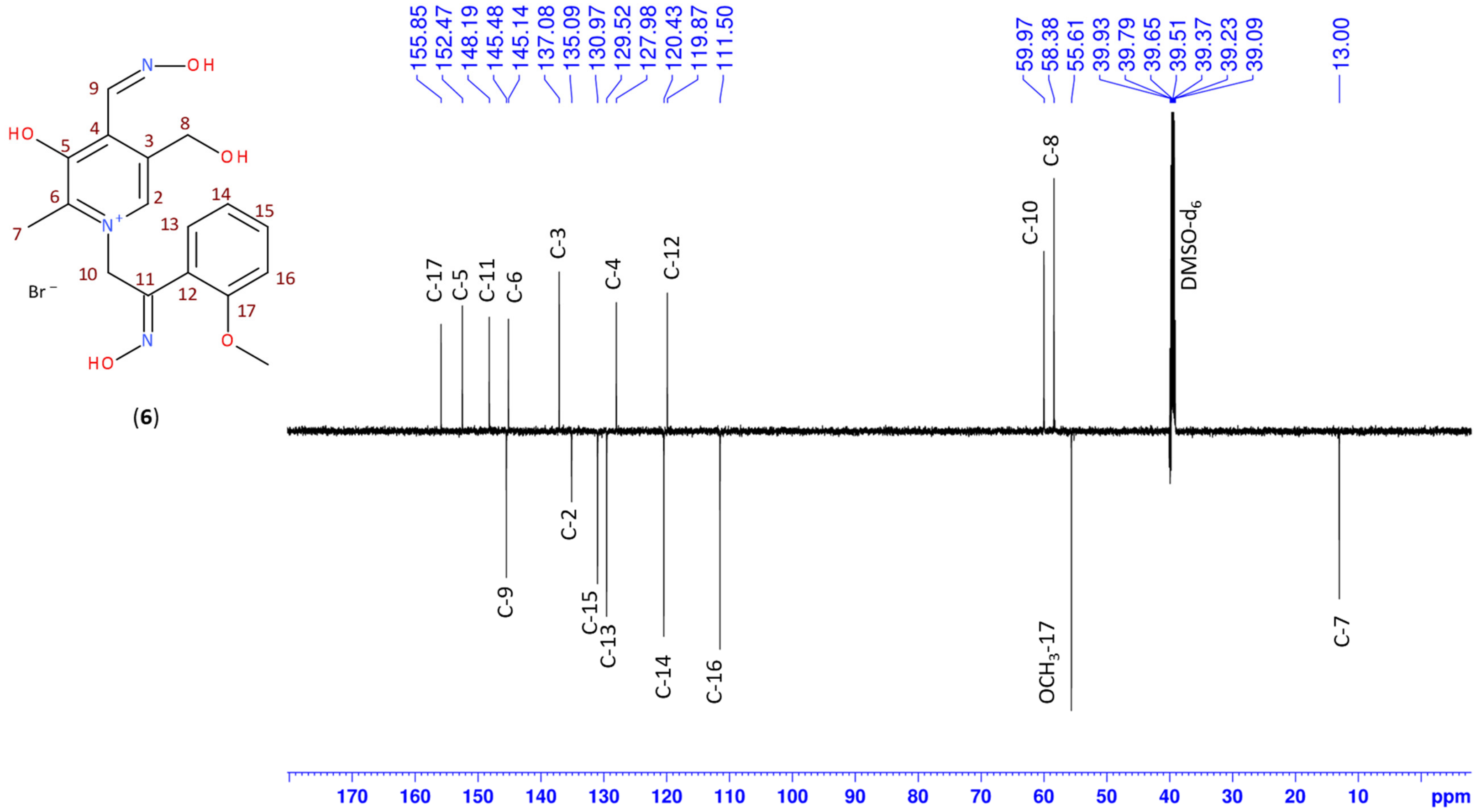

Figure S27. 150 MHz ^{13}C APT spectrum of compound **6** in DMSO-d_6.

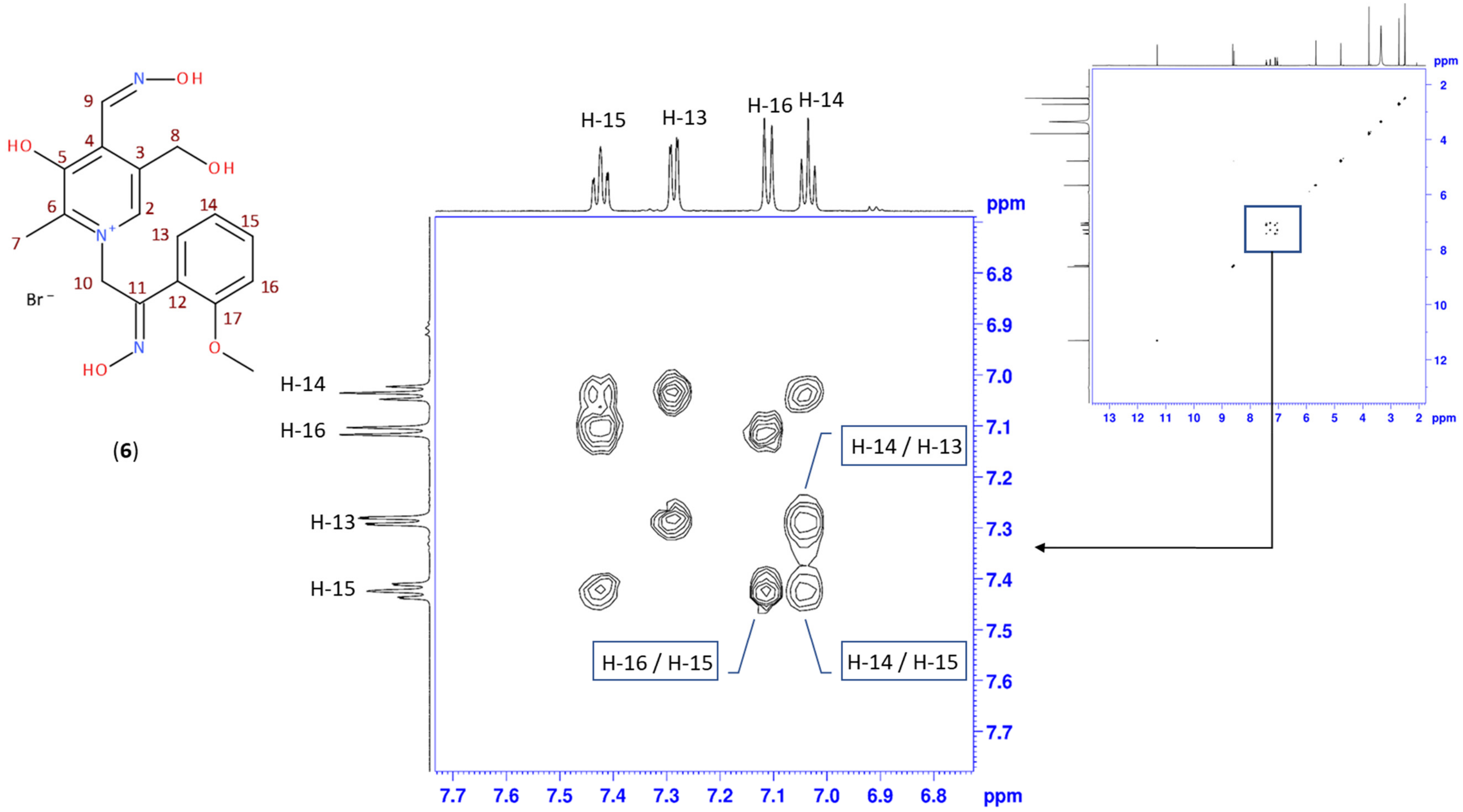

Figure S28. 600 MHz H,H-COSY NMR spectrum of the isomer mixture (a and b) of compound **6** in DMSO-d_6. The one-dimensional ^{1}H spectrum is shown at the top and on the left.

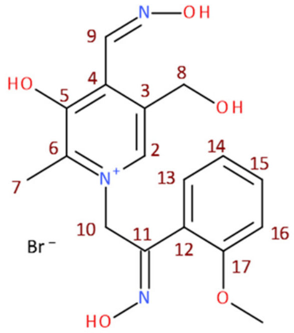

(**6**)

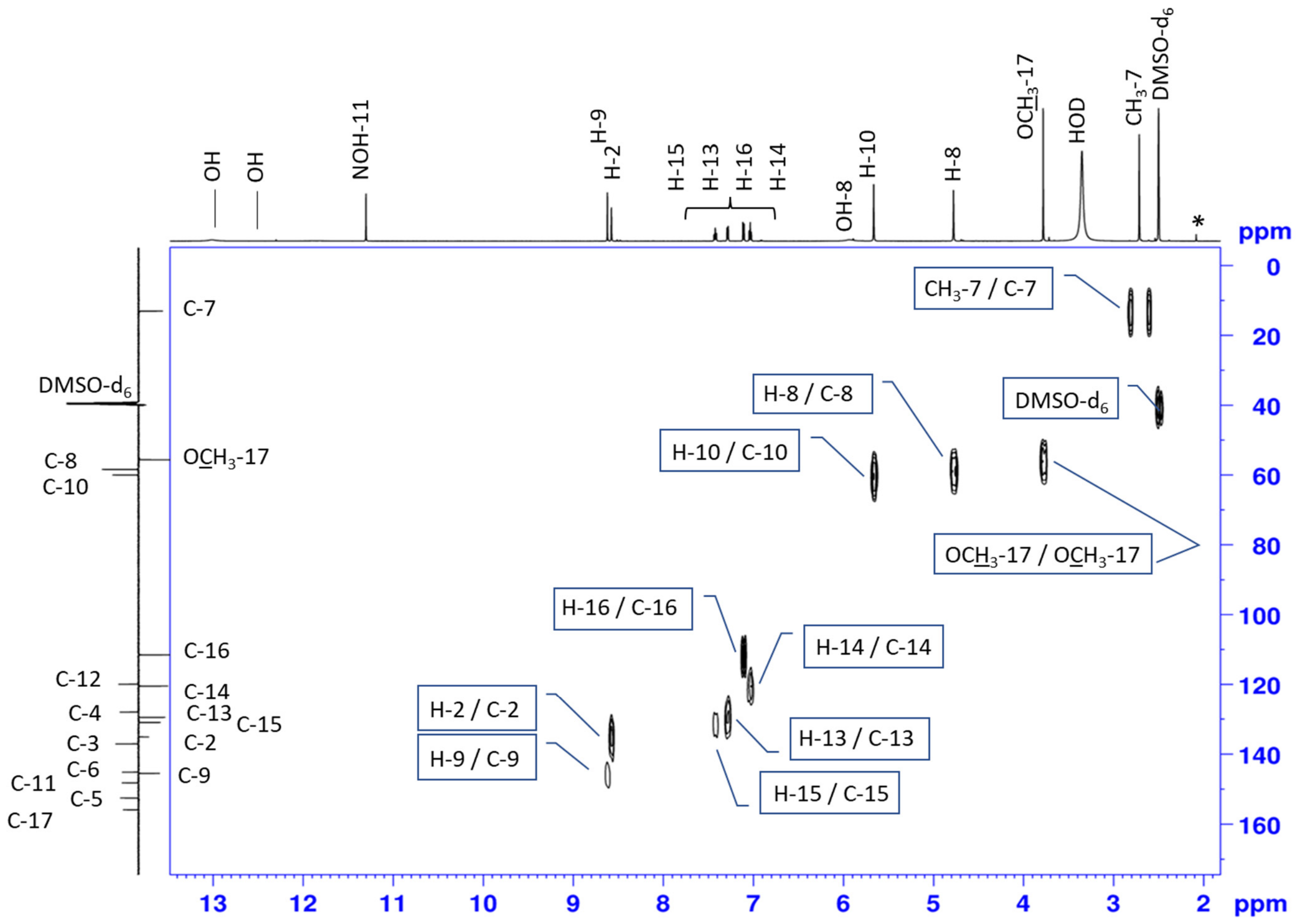

Figure S29. Two-dimensional H,C-correlated spectra of the isomer mixture (a and b) of compound **6** recorded by HMQC method in DMSO-d_6. The 600 MHz ^{1}H spectrum is shown at the top and 125 MHz 13C NMR spectrum at the left-hand edge.

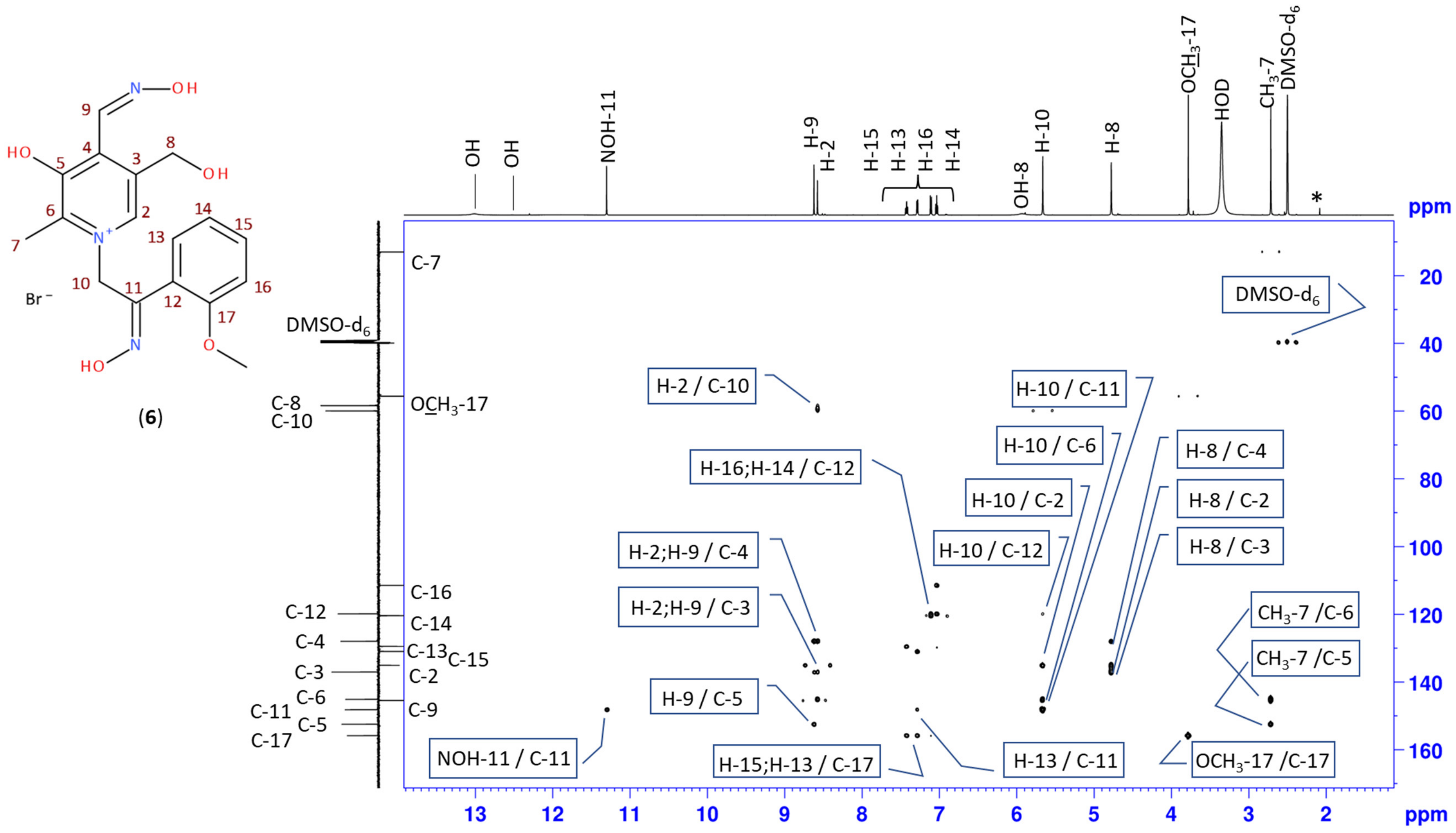

Figure S30. Two-dimensional H,C-correlated spectra of the isomer mixture (a and b) of compound **6** recorded by HMBC method in DMSO-d_6. The 600 MHz ^{1}H spectrum is shown at the top and 125 MHz 13C NMR spectrum at the left-hand edge.

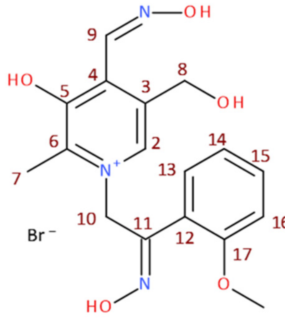

(**6**)

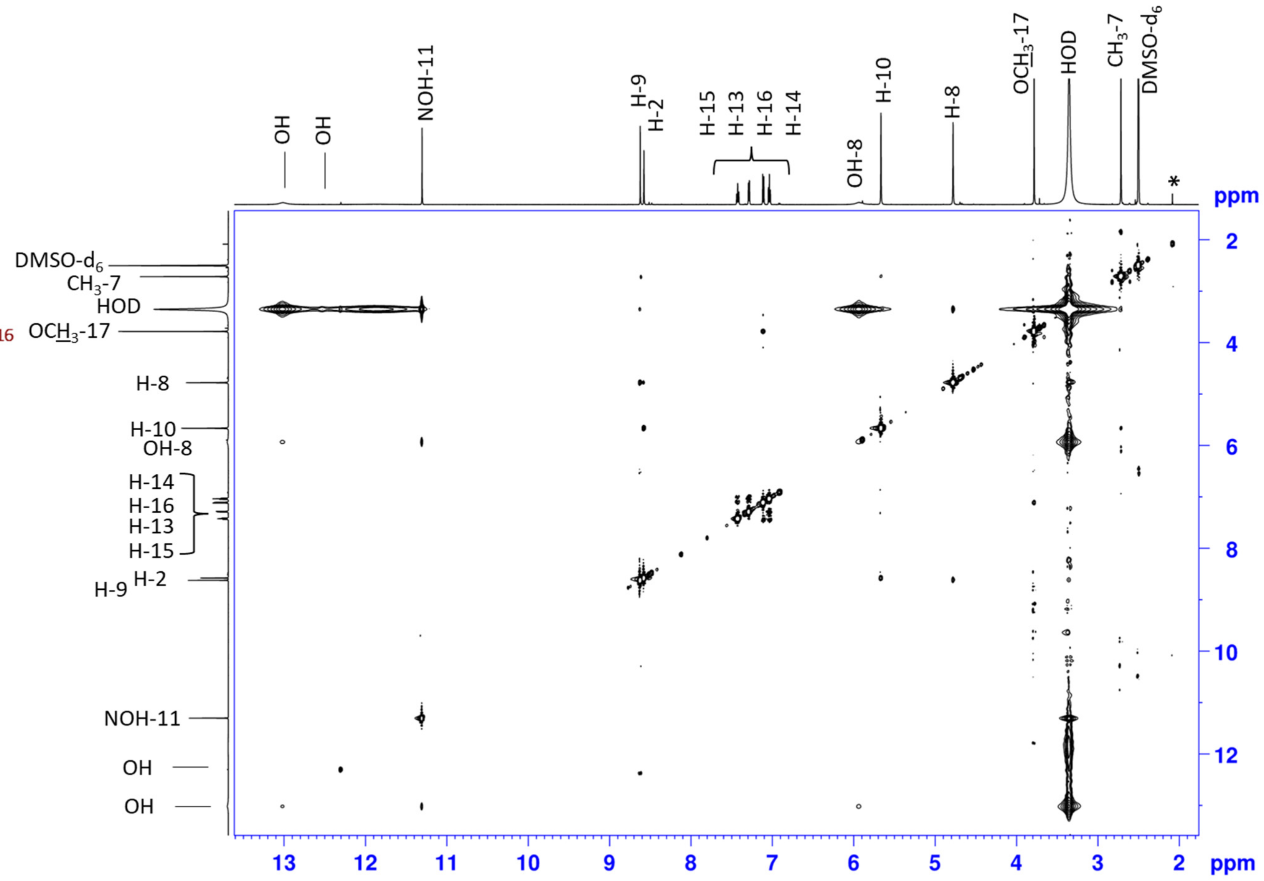

Figure S31. 600 MHz NOESY NMR spectrum of the isomer mixture (a and b) of compound **6** in DMSO-d_6. The one-dimensional ^{1}H spectrum is shown at the top and on the left.

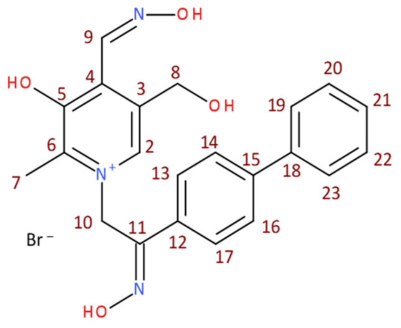

(**7**)

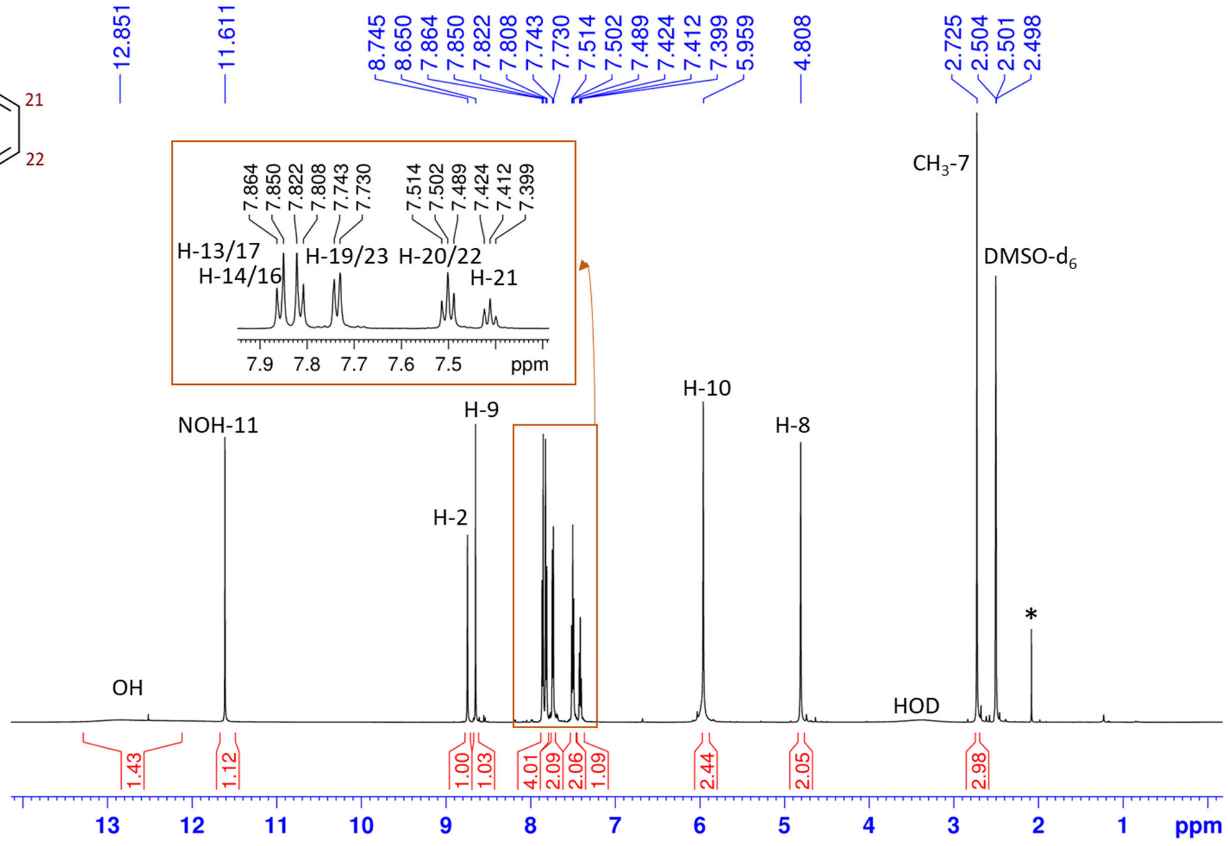

Figure S32. 600 MHz ^{1}H NMR spectrum of compound **7** in DMSO-d$_6$. Acetone left after synthesis is marked with an asterisk.

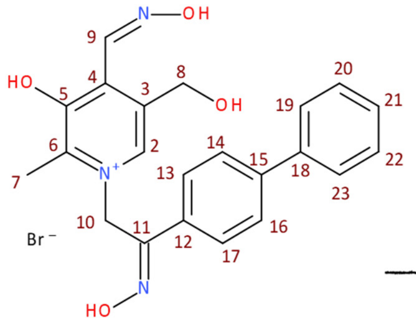

(**7**)

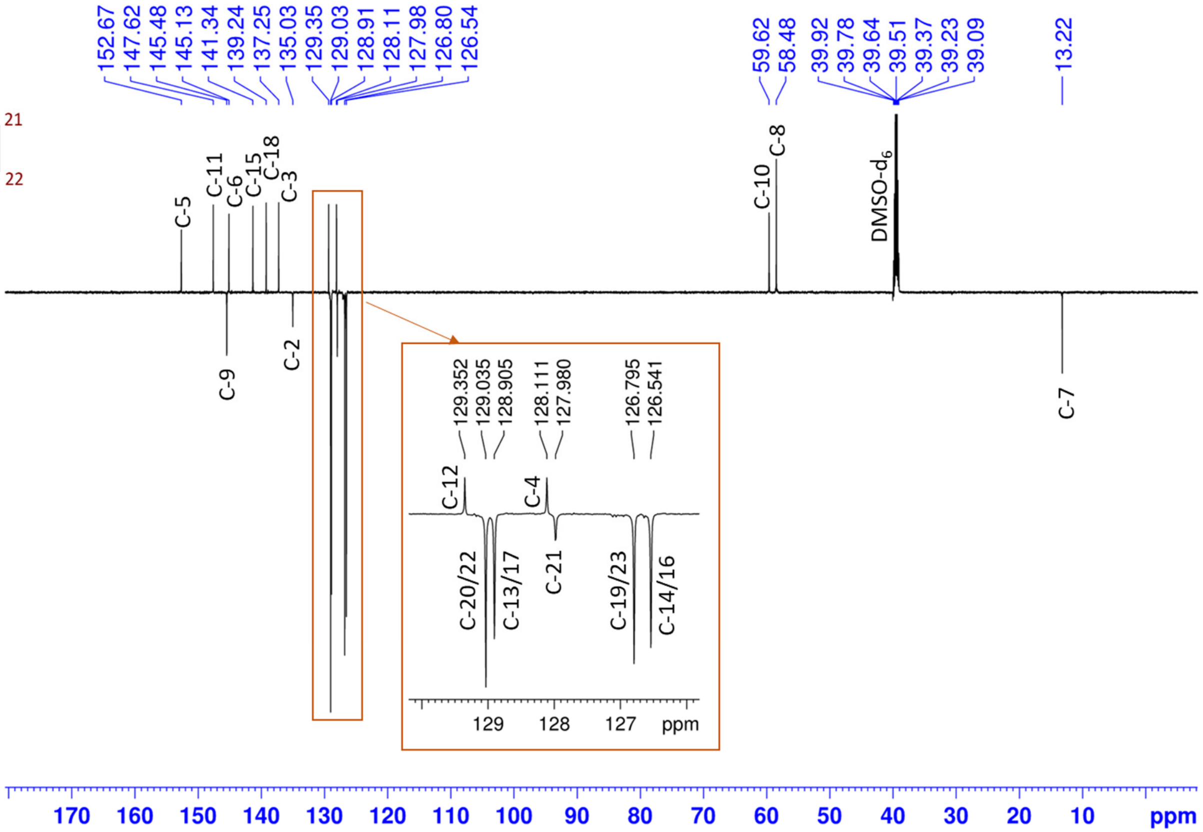

Figure S33. 150 MHz ^{13}C APT spectrum of compound **7** in DMSO-d_6.

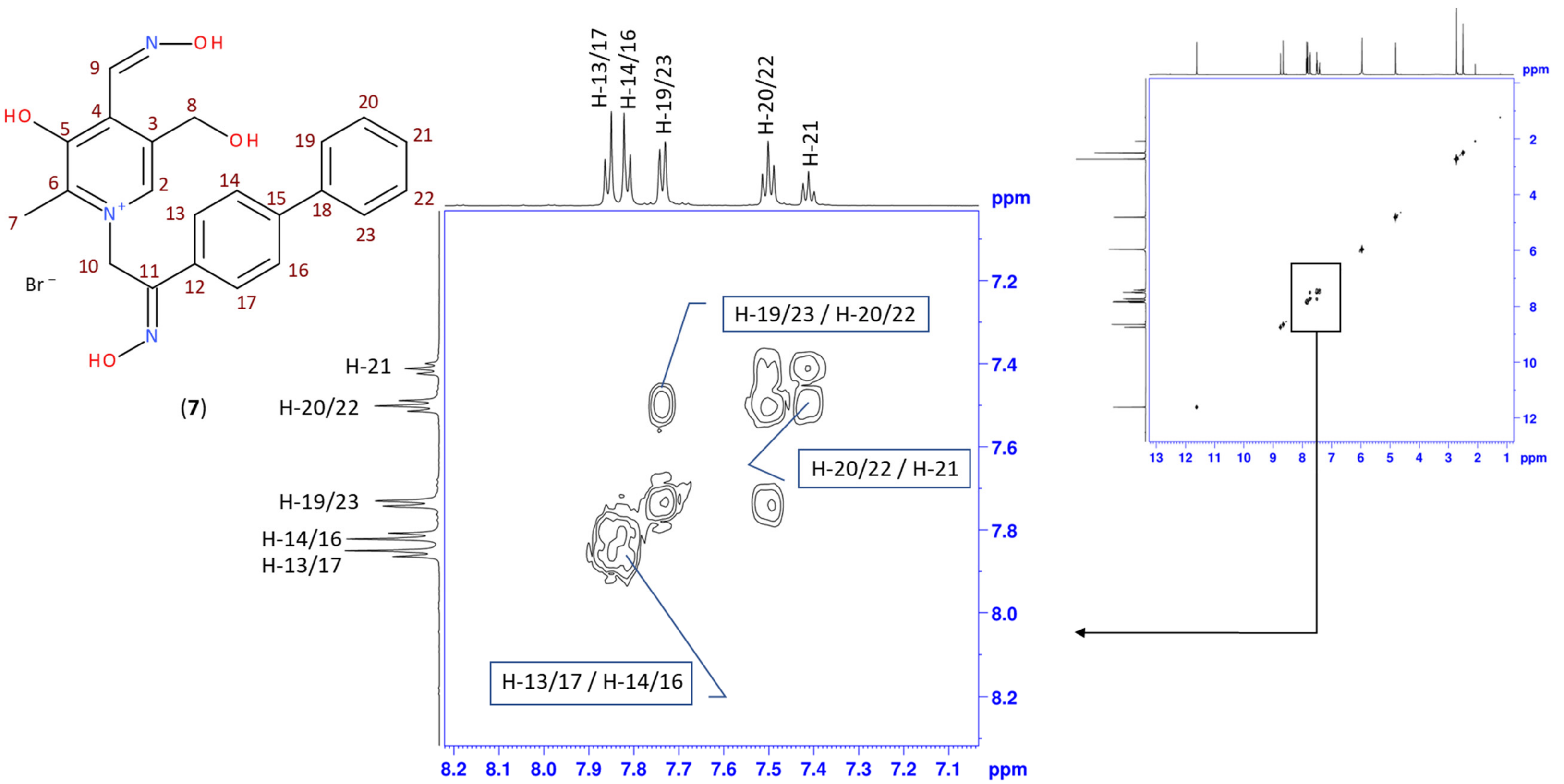

Figure S34. 600 MHz H,H-COSY NMR spectrum of the isomer mixture (a and b) of compound **7** in DMSO-d_6. The one-dimensional ^{1}H spectrum is shown at the top and on the left.

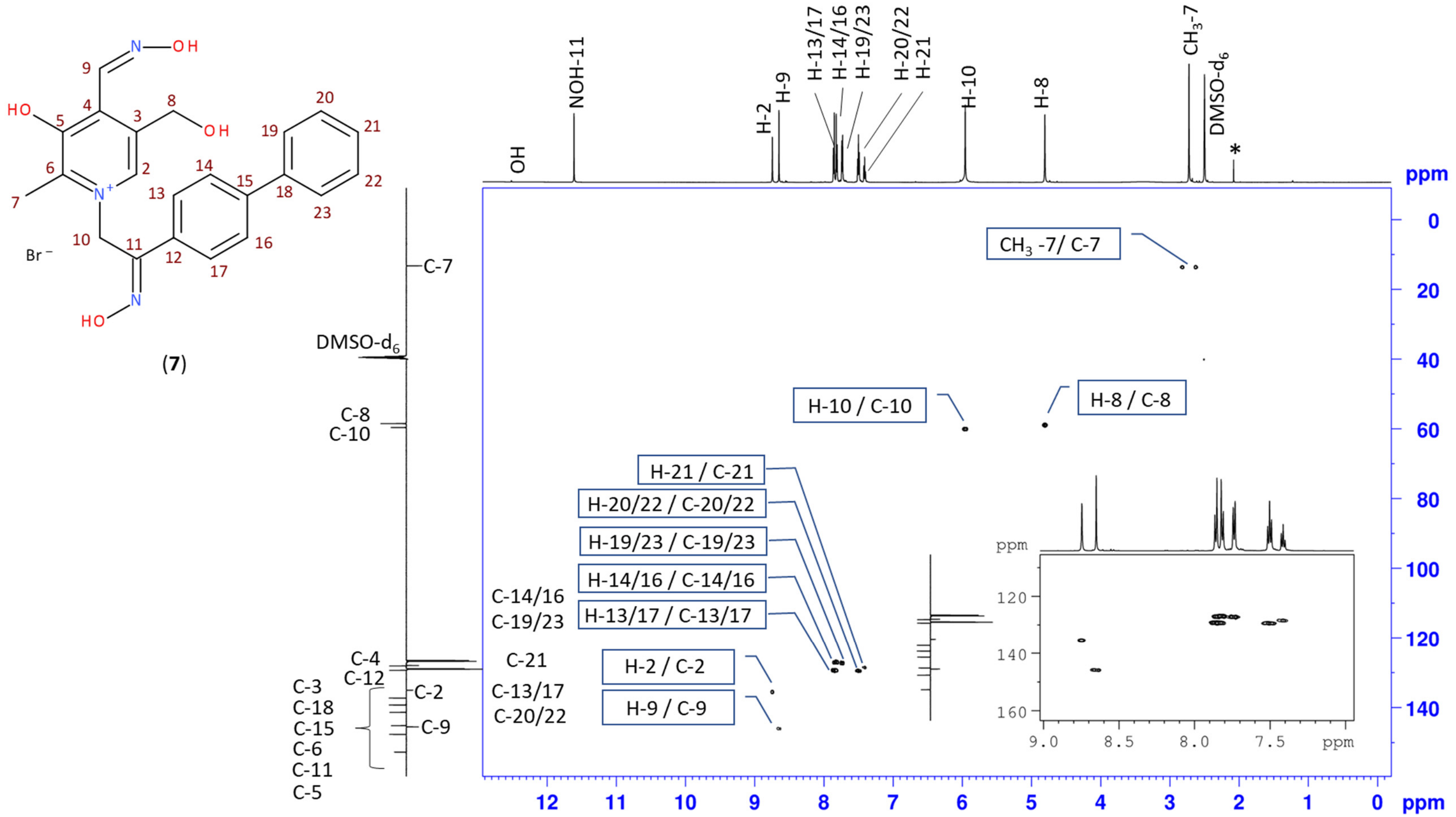

Figure S35. Two-dimensional H,C-correlated spectra of compound **7** recorded by HMQC method in DMSO-d_6. The 600 MHz ^{1}H spectrum is shown at the top and 125 MHz 13C NMR spectrum at the left-hand edge.

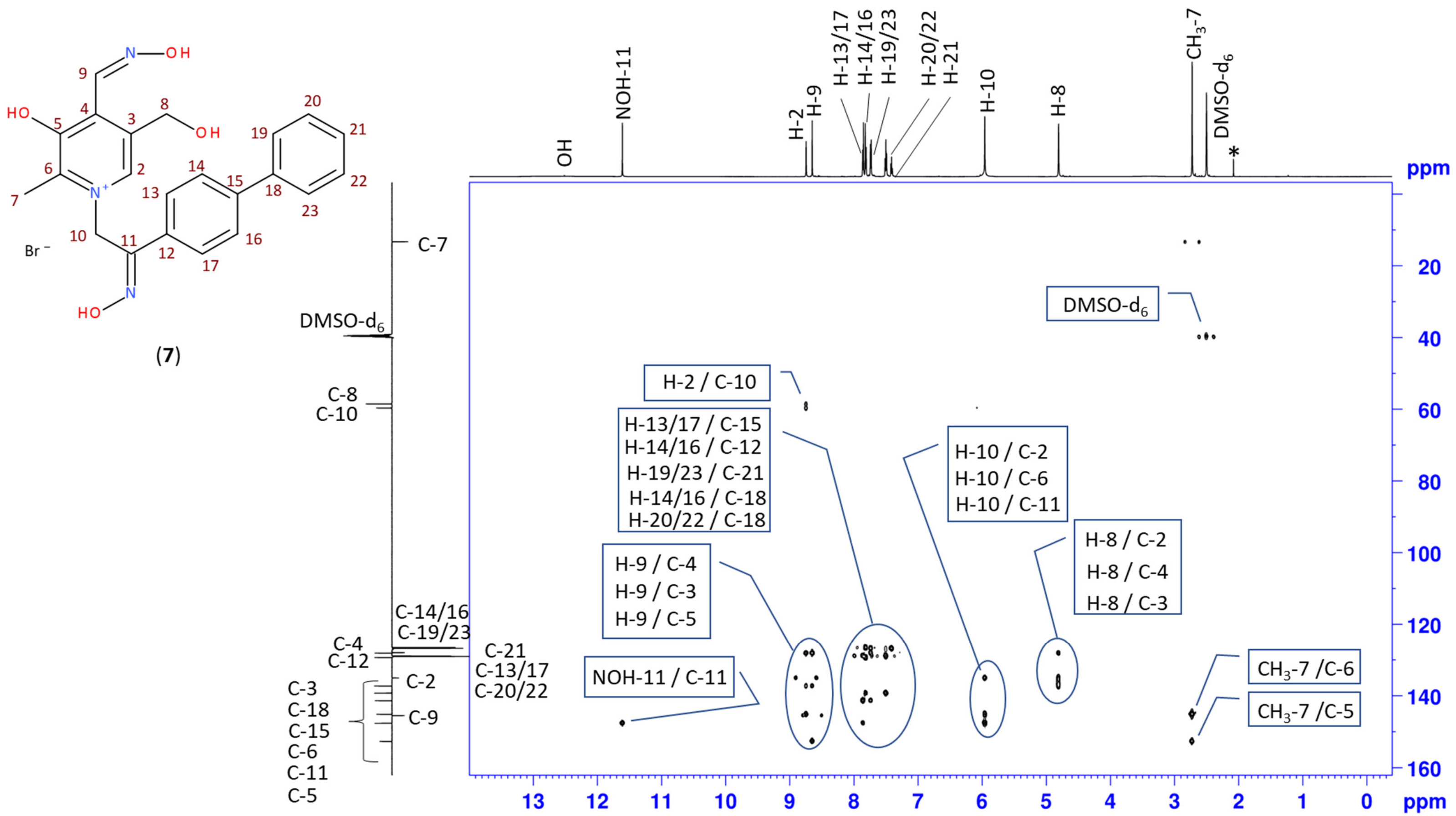

Figure S36. Two-dimensional H,C-correlated spectra of compound **7** recorded by HMBC method in DMSO-d_6. The 600 MHz ^{1}H spectrum is shown at the top and 125 MHz 13C NMR spectrum at the left-hand edge.

MS spectra of prepared compounds (1 – 7)

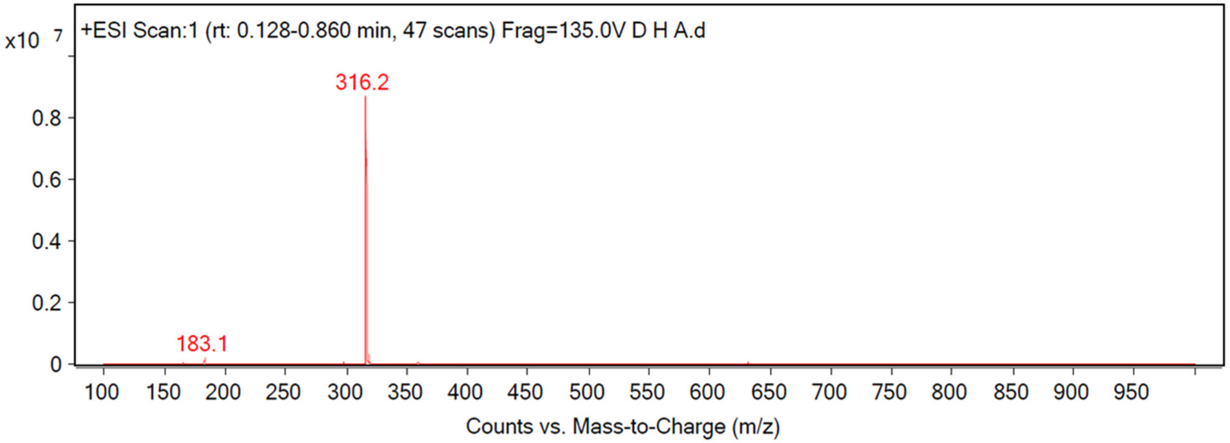

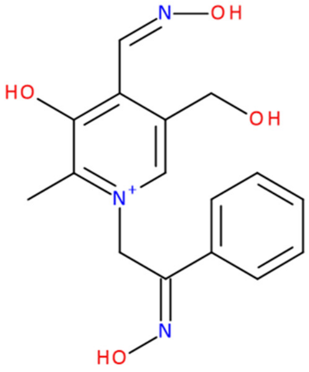

Formula Weight: 316,33182
Exact Mass: 316,129731086
Molecular Formula: $C_{16}H_{18}N_3O_4$

Figure **S37**. Mass spectrum (ESI$^+$) of compound **1**.

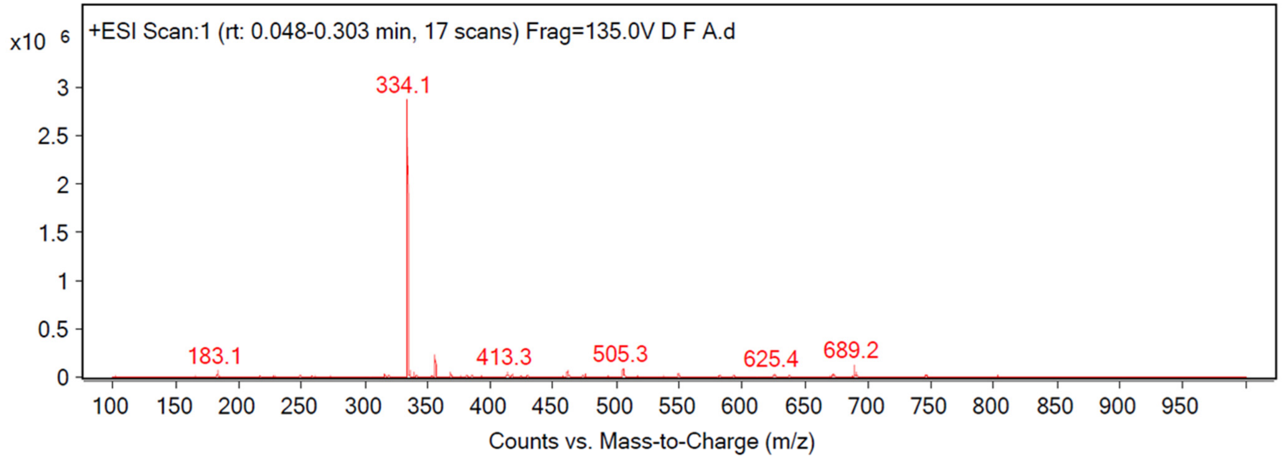

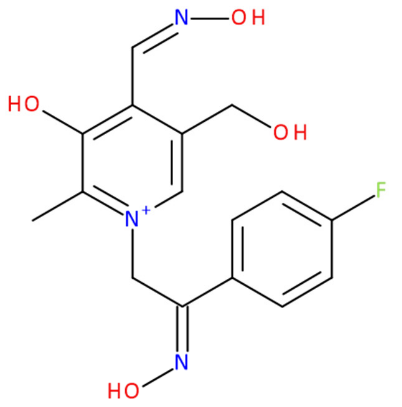

Formula Weight: 334,32228
Exact Mass: 334,120309254
Molecular Formula: $C_{16}H_{17}FN_3O_4$

Figure **S38**. Mass spectrum (ESI$^+$) of compound **2**.

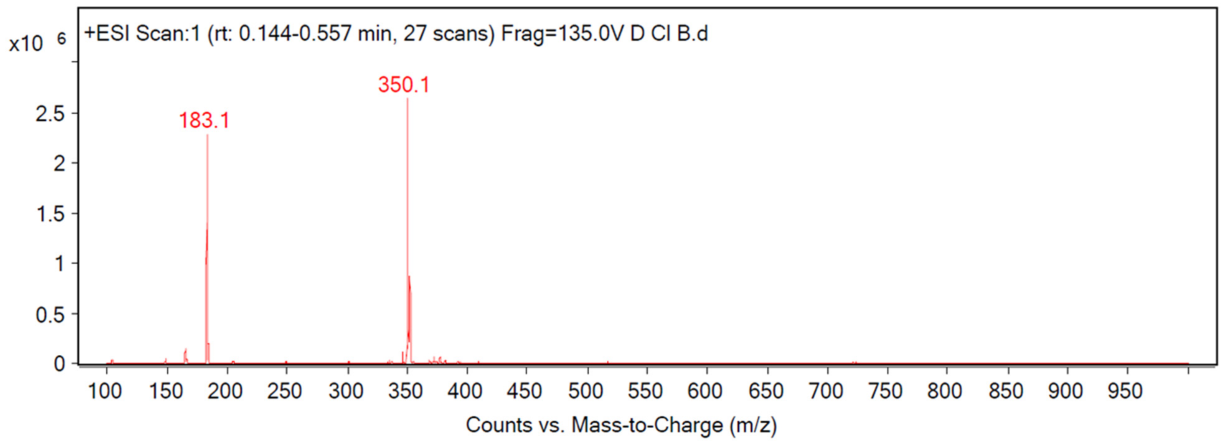

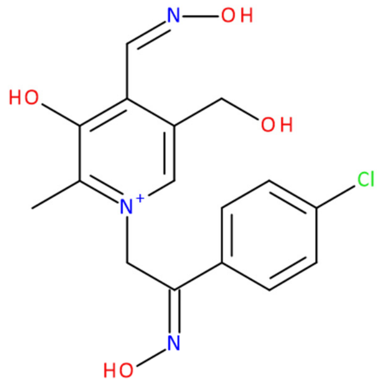

Formula Weight: 350,77687
Exact Mass: 350,090758764
Molecular Formula: $C_{16}H_{17}ClN_3O_4$

Figure S39. Mass spectrum (ESI$^+$) of compound **3**.

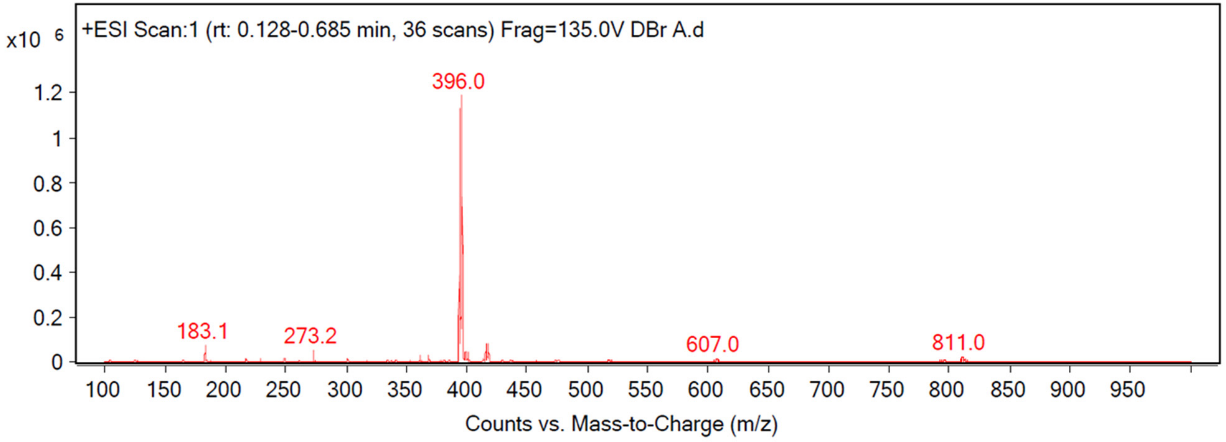

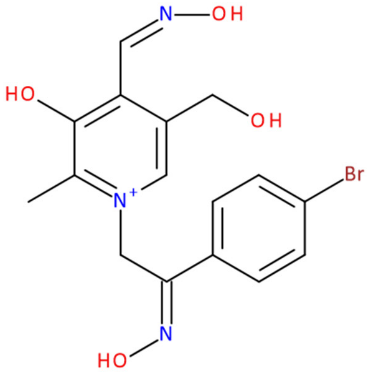

Formula Weight: 395,22787
Exact Mass: 394,040243654
Molecular Formula: $C_{16}H_{17}BrN_3O_4$

Figure S40. Mass spectrum (ESI^+) of compound **4**.

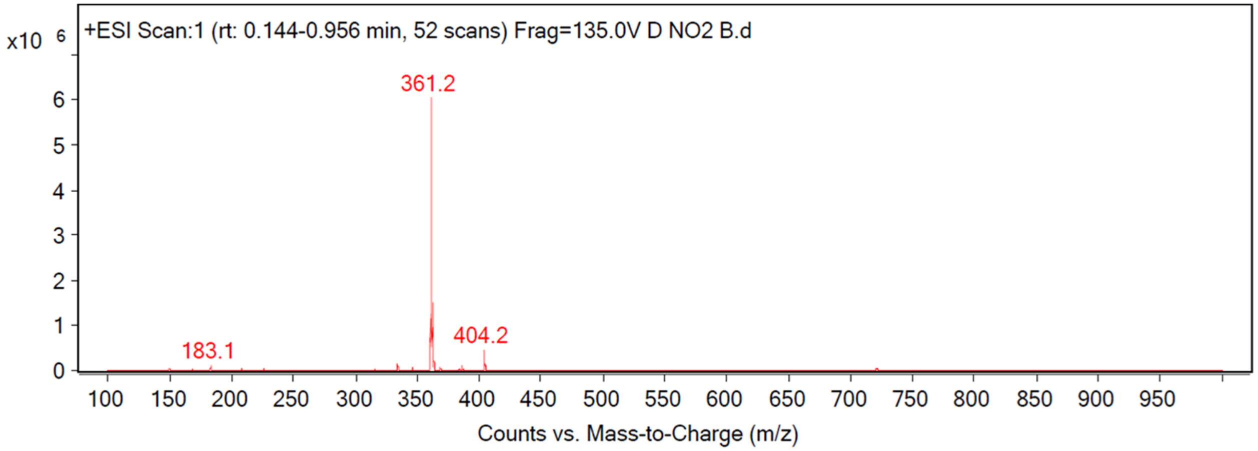

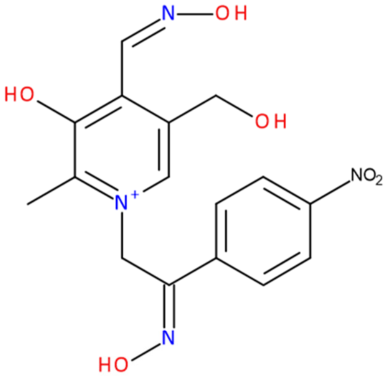

Formula Weight: 361,32938
Exact Mass: 361,114809304
Molecular Formula: $C_{16}H_{17}N_4O_6$

Figure S41. Mass spectrum (ESI^+) of compound **5**.

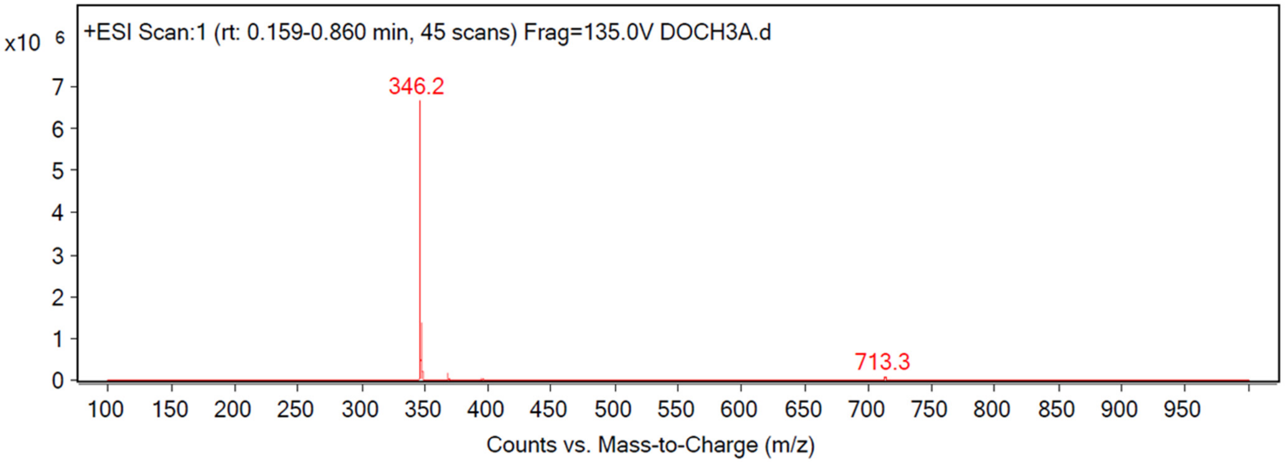

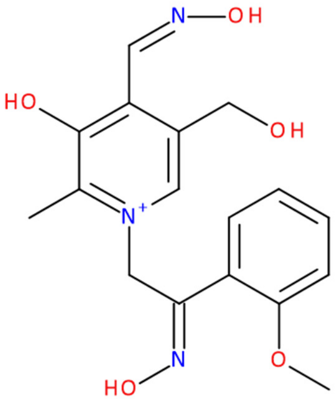

Figure S42. Mass spectrum (ESI$^+$) of compound **6**.

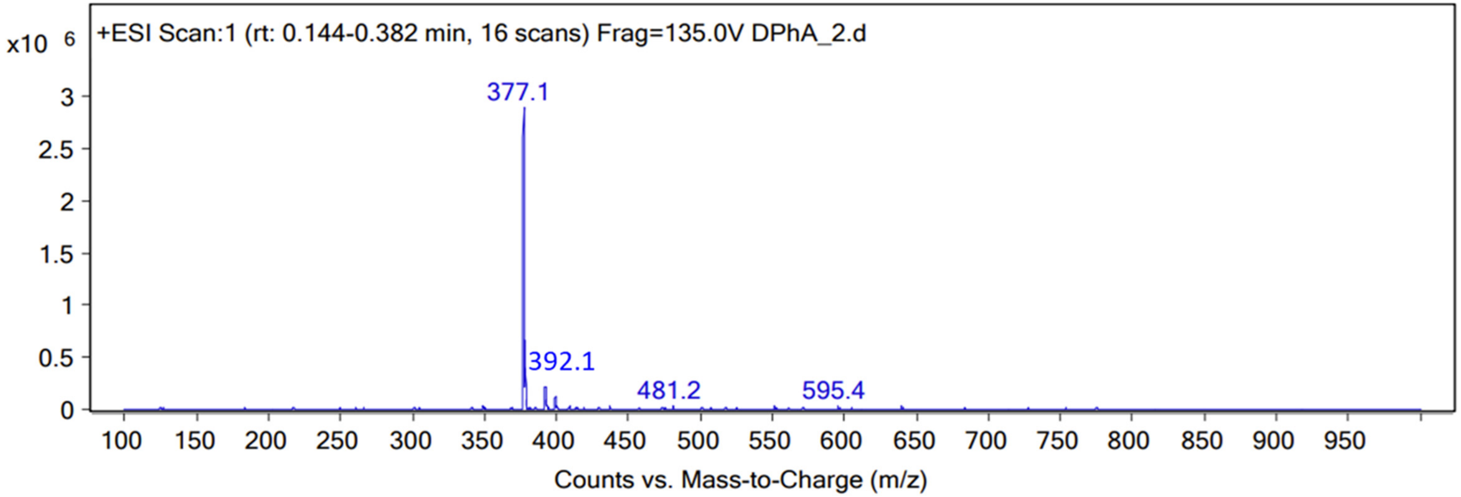

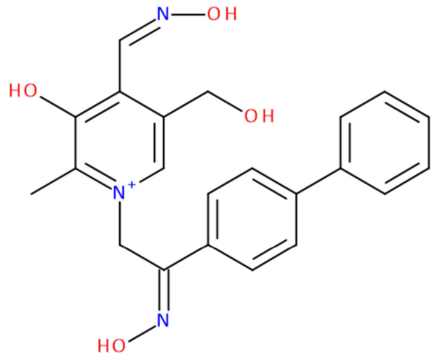

Formula Weight: 392,42778
Exact Mass: 392,161031214
Molecular Formula: $C_{22}H_{22}N_3O_4$

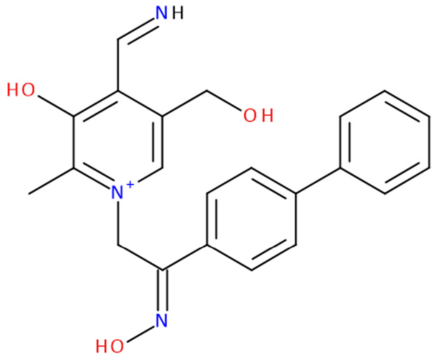

Formula Weight: 376,42838
Exact Mass: 376,166116594
Molecular Formula: $C_{22}H_{22}N_3O_3$

Figure S43 Mass spectrum (ESI$^+$) of compound **7**.

Interactions between compound 1 and AChE, and compound 7 and AChE / BChE

Table S1. Interactions between compound **1** and AChE.

Amino acid	Non-bonding interactions
HOH869	N12, hydrogen bond-water hydrogen bond; conventional hydrogen bond
HOH869	H26, hydrogen bond-water hydrogen bond; conventional hydrogen bond
HOH809	H30, hydrogen bond-water hydrogen bond; carbon hydrogen bond
Gly122	O23, hydrogen bond-conventional hydrogen bond
Tyr124	N12, hydrogen bond-conventional hydrogen bond
Asp74	H26, hydrogen bond-conventional hydrogen bond
Tyr124	H32, hydrogen bond-conventional hydrogen bond
Gly121	O23, hydrogen bond-carbon hydrogen bond
Asp74	H25, hydrogen bond-carbon hydrogen bond
Tyr341	B8, hydrophobic-π-π stacked

Table S2. Interactions between compound **7** and AChE.

Amino acid	Non-bonding interactions
Gly122	O25, hydrogen bond-conventional hydrogen bond
Ser203	H32, hydrogen bond-conventional hydrogen bond
His447	H38, hydrogen bond-conventional hydrogen bond
Gly121	O13, hydrogen bond-carbon hydrogen bond
Glu202	H31, hydrogen bond-carbon hydrogen bond
His447	H31, hydrogen bond-carbon hydrogen bond
Phe338	H46 , hydrogen bond-π-donor hydrogen bond
Tyr124	B8, other-π-lone pair
Tyr72	B8, hydrophobic-π-π stacked
Tyr337	B7, hydrophobic-π-π T-shaped

Table S3. Interactions between compound **7** and BChE.

Amino acid	Non-bonding interactions
Asp70	N3, electrostatic-attractive charge
HOH736	N16, hydrogen bond-water hydrogen bond; conventional hydrogen bond
HOH910	N16, hydrogen bond-water hydrogen bond; conventional hydrogen bond
HOH930	O11, hydrogen bond-water hydrogen bond; conventional hydrogen bond
Gln119	O8, hydrogen bond-conventional hydrogen bond
Thr284	H39, hydrogen bond-conventional hydrogen bond
HOH930	B8, other-π-lone pair
Trp82	B8, hydrophobic-π-π stacked
Trp82	B8, hydrophobic-π-π stacked
Trp82	B8, hydrophobic-π-π T-shaped
